# Supplementary figures and images for: A cluster of Ankyrin and Ankyrin-TPR repeat genes is associated with panicle branching diversity in rice
Source: PLoS Genet. 2021 Jun 7;17(6):e1009594. doi: 10.1371/journal.pgen.1009594 (PMC8211194; doi:10.1371/journal.pgen.1009594)

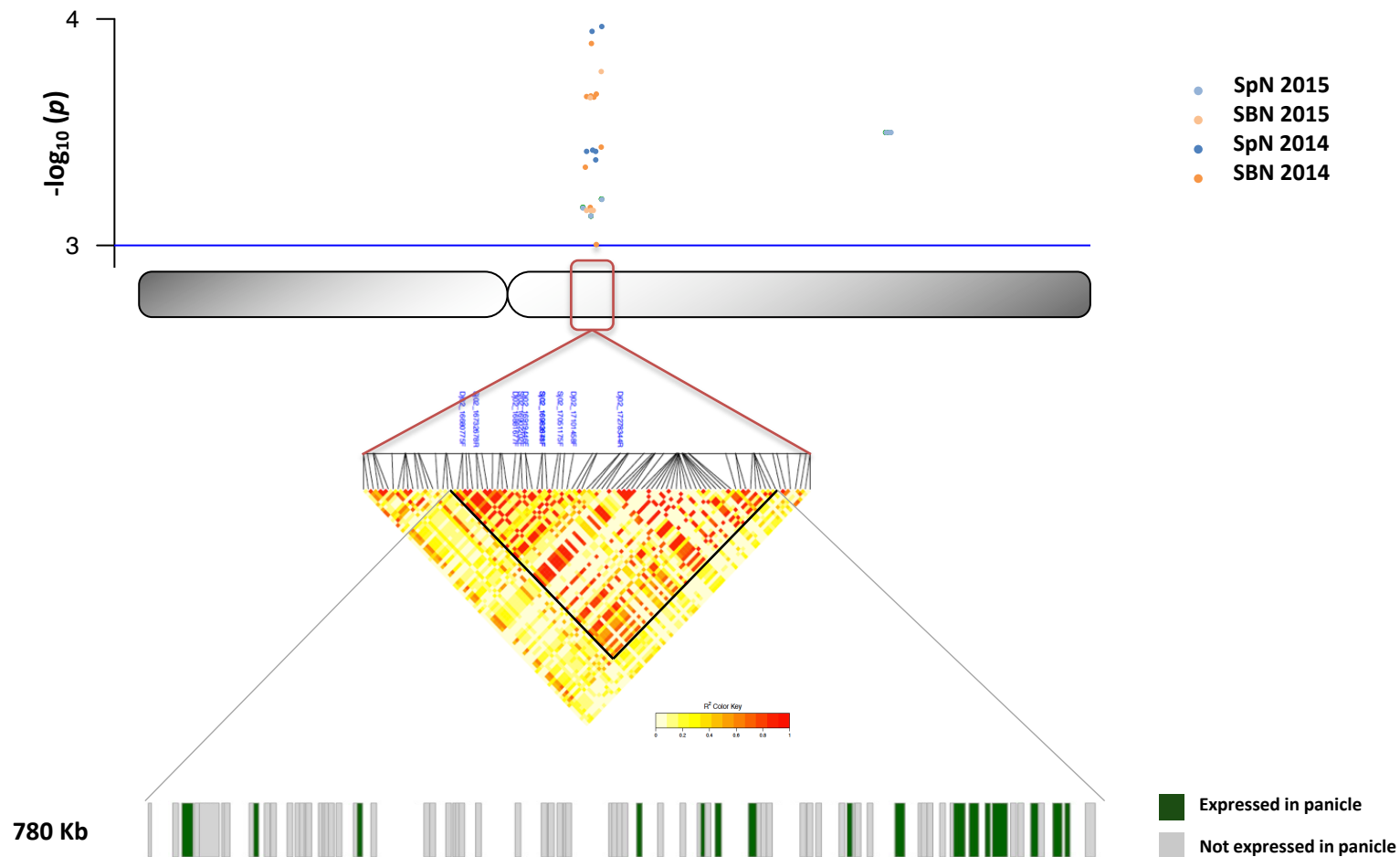

Supplement: S1 Fig — Manhattan-plot and Linkage Disequilibrium (LD) heatmap of chromosome 2 showing significant SNPs (p-value threshold 10−3) in the QTL_9 region for the characters spikelet number (SpN) and secondary branch number (SBN) per panicle for the two field trials performed in 2014 and 2015 according to [26]. Red and bold back lines on the LD heat maps delimit the LD block for the GWAS peak. The significant SNPs are labelled in blue in the LD heatmap. The lower panel shows the annotated genes according to the O. sativa ssp. japonica cv. Nipponbare MSU7.0 reference genome within the 783 Kbp region corresponding to QTL_9, indicating genes that are expressed (green) or not expressed (grey) in the developing panicle according the publicly available databases and RNA-seq dataset. (PDF) [file pgen.1009594.s001.pdf]

**A**

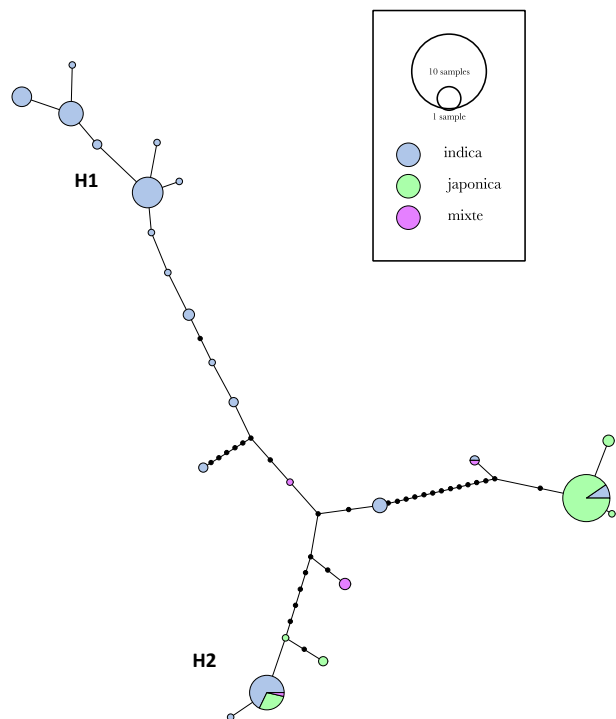

**B**

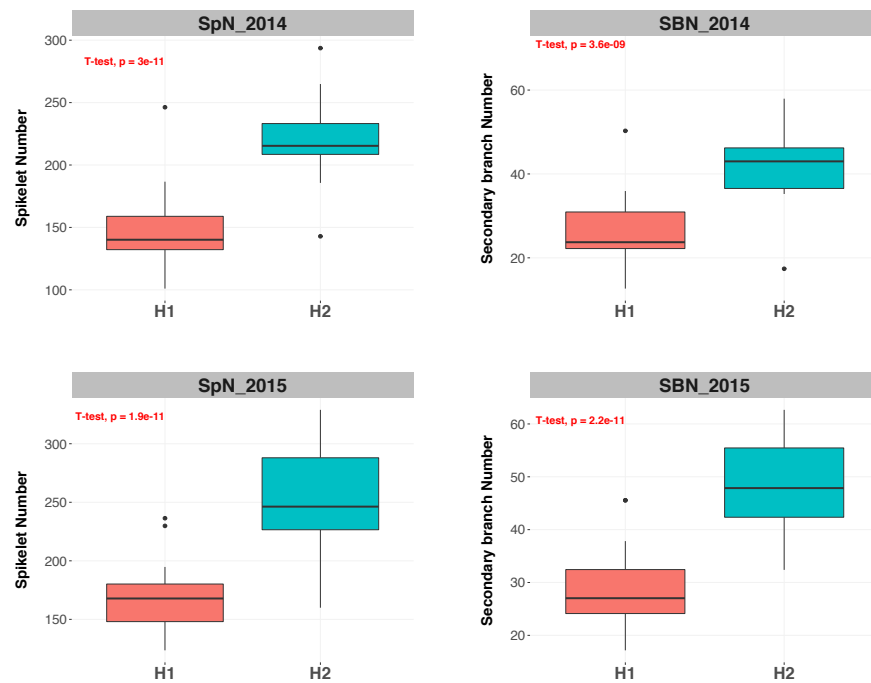

Supplement: S2 Fig — (A) Relationship dendrogram of the different haplotypes based on the analysis of the polymorphic sites from QTL_9 region used for the GWAS analysis in the Vietnamese landrace collection [26]. The proportion of indica, japonica and admixture accessions for each haplotype is indicated. The two main haplotypes, H1 and H2, are indicated. (B) Box-plots of the characters spikelet number (SpN) and secondary branch number (SBN) per panicle evaluated in 2014 and 2015 in the accessions from haplotypes H1 and H2. Statistical significance (i.e. t test p values) between the two haplotypes for the two panicle morphological traits is indicated in each case. (PDF) [file pgen.1009594.s002.pdf]

2015\_pheatmap\_euclid\_wardD2

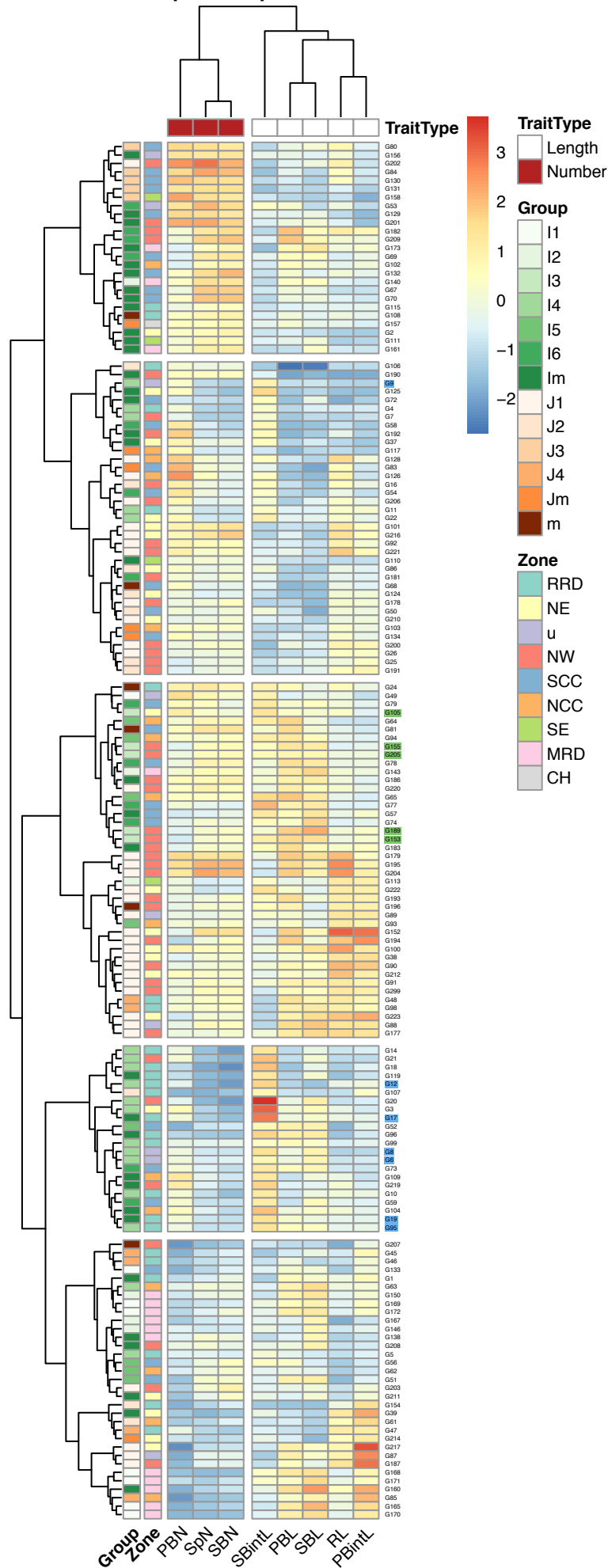

Supplement: S3 Fig — Euclidiean_WardD2 heatmap based on the phenotypic values obtained in 2015. Group: genetic group of the accessions according to [68] for indica (I1 to I6), japonica (J1 to J4) and admixture (m, Im, Jm); Zone: region from Vietnam where the accession was originating (MRD = Mekong River Delta; SE = Southeast; CH = Central Highlands; SCC = South Central Coast; NCC = North Central Coast; RRD = Red River Delta; NW = Northwest; NE = Northeast; u = unknown); Trait types: red for number related traits (PBN, primary branch number; SBN, secondary branch number; SpN; spikelet number), white for length-related traits (RL, rachis length; PBL, primary branch average length; PBintL, average primary branch internode length; SBL, secondary branch average length; SBintL, average secondary branch internode length). The accessions from haplotypes H1 and H2 are highlighted in blue and green respectively. (PDF) [file pgen.1009594.s003.pdf]

**A**

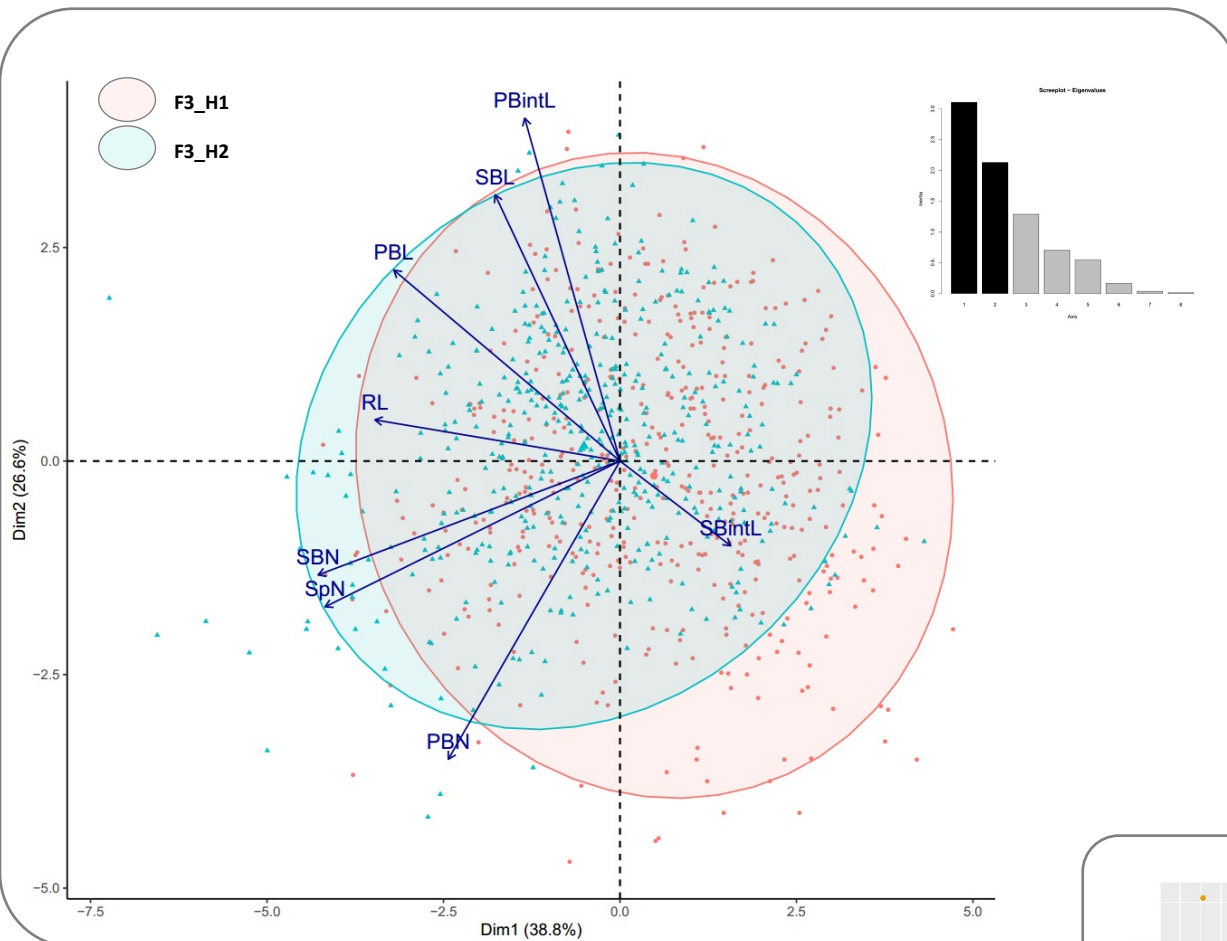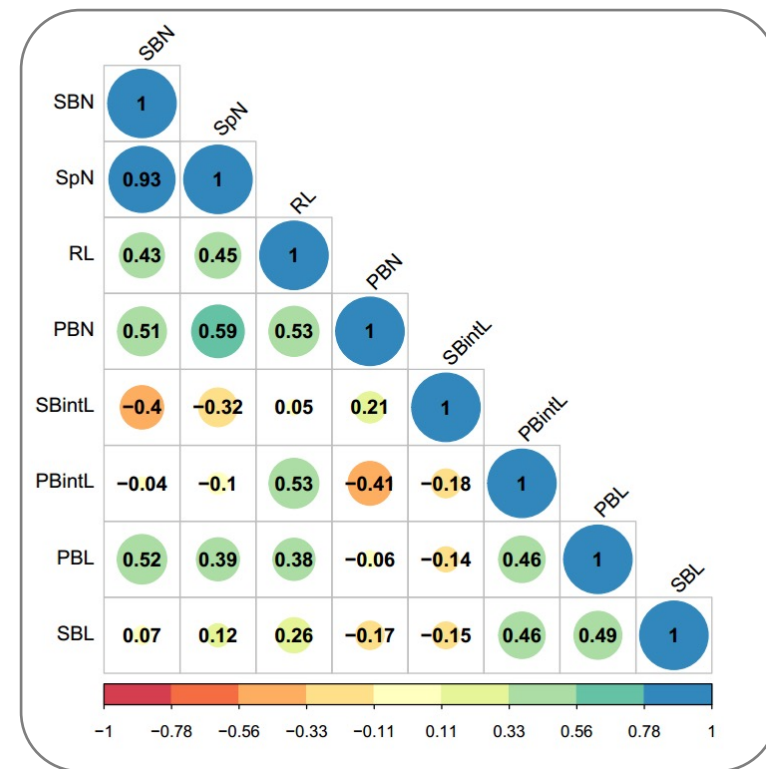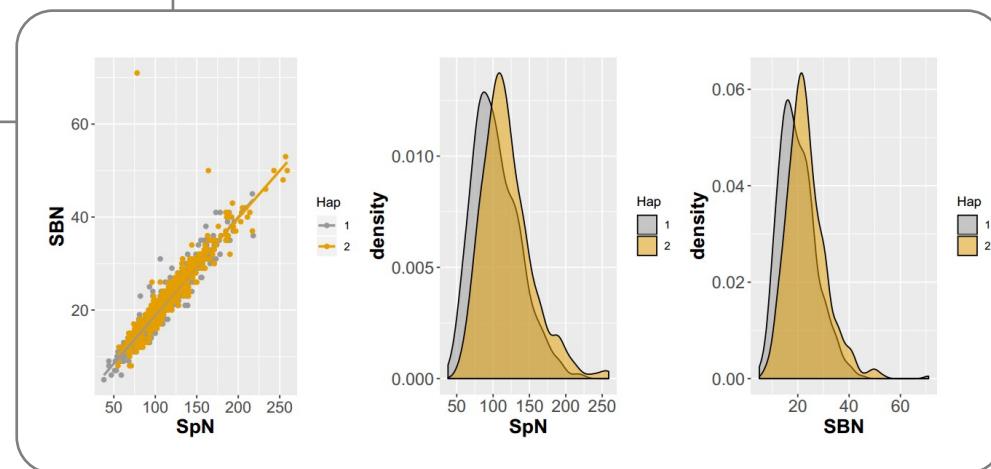

**B**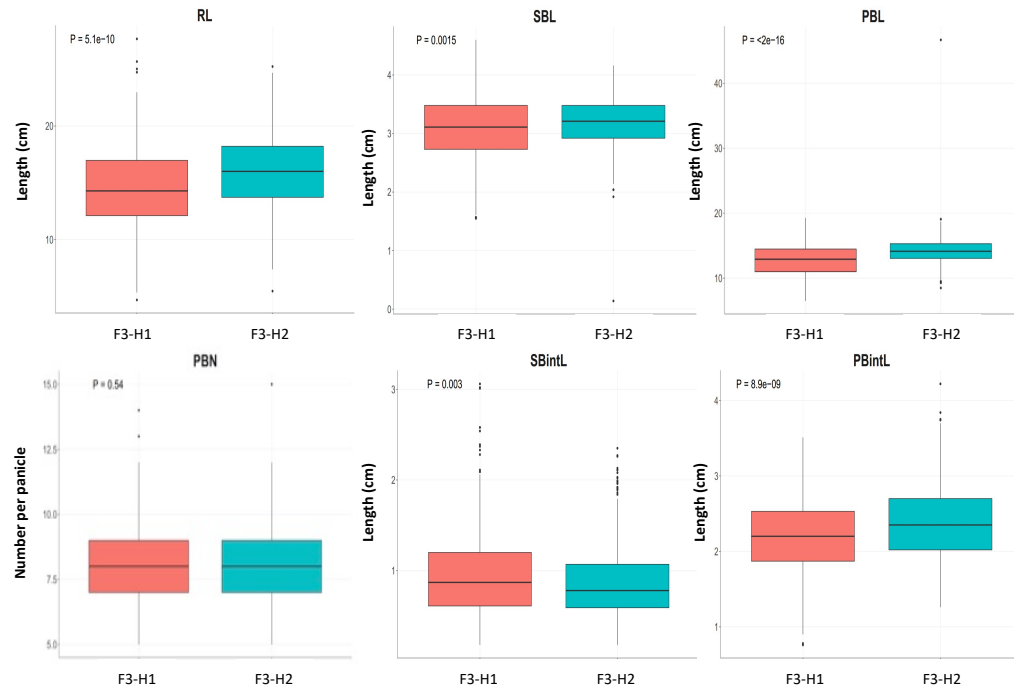**C**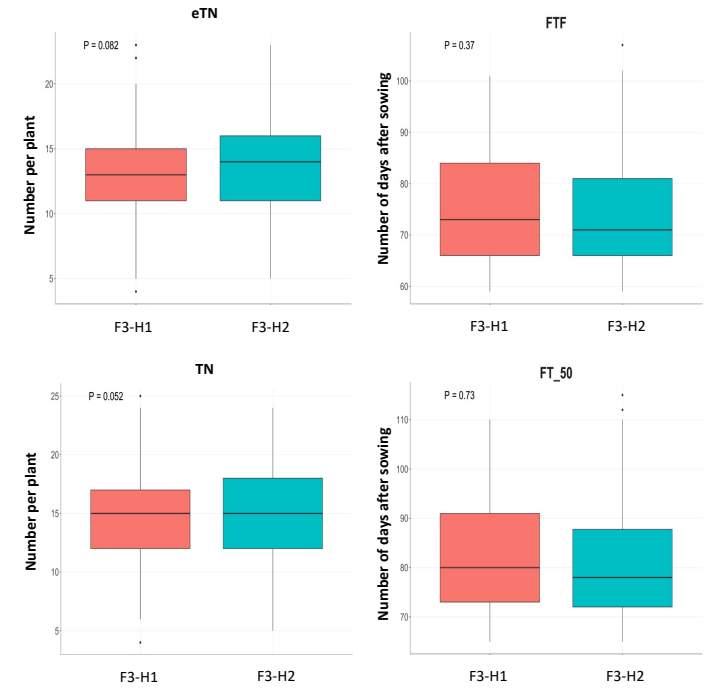

Supplement: S4 Fig — (A) Panicle morphological trait analysis in F3 plants homozygous for the two haplotypes in QTL_9 region. Left panel: Principal Component Analysis (PCA) analysis for the two first axis. Right upper panel: correlation plots of the different panicle morphological traits. Right lower panel: correlation between the SpN and the SBN traits in the two F3 subpopulations (i.e. for haplotype H1 and H2). (B) Box-plots of the panicle morphological trait values in the F3 lines from the G6xG189 bi-parental population with H1 haplotype (F3_H1) or H2 haplotype (F3_H2) in the QTL_9 region: rachis length (RL), average secondary branch length (SBL), average primary branch length (PBL), primary branch number (PBN), secondary branch internode average length (SBintL), average primary branch internode length (PBintL). (C) Box-plots for tiller number (TN), efficient tiller number (eTN), flowering date of the first panicle (FTF), flowering date for 50% of the plants (FT_50). Statistical significance (i.e. t test p values) between the two haplotypes for the two panicle morphological traits is indicated. (PDF) [file pgen.1009594.s004.pdf]

**A**

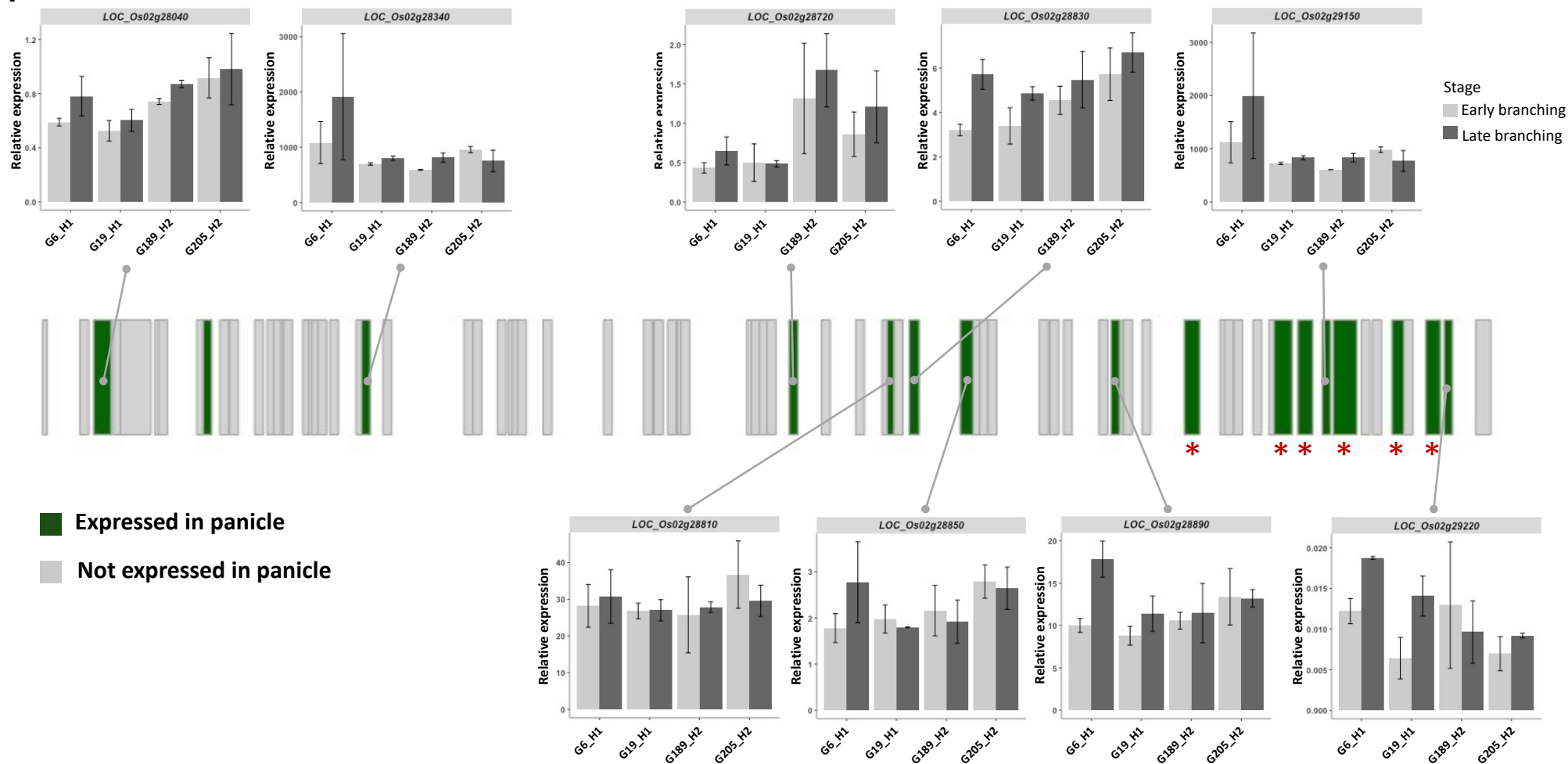

**B**

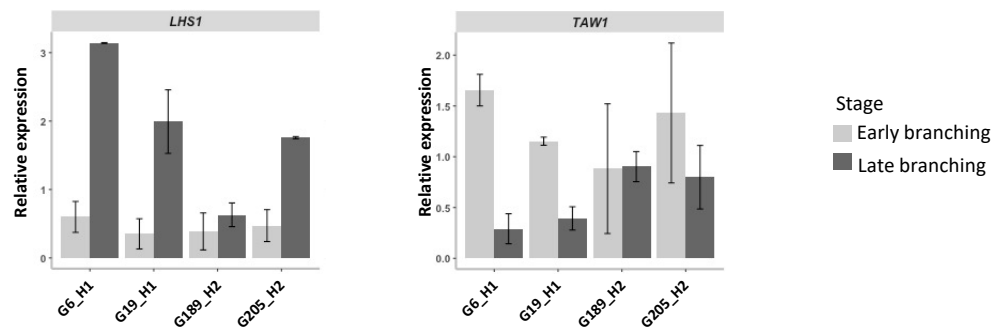

Supplement: S5 Fig — (A) schematic view of the 780 Kbp QTL_9 region showing annotated genes according to the O. sativa ssp. japonica cv. Nipponbare MSU7.0 reference genome. Genes expressed in the developing panicle are indicated in green. The ANK and ANK-TPR genes are indicated by red asterisks. Histograms illustrate the expression profiling data by qRT-PCR of the panicle-expressed genes which do not belong to ANK or ANK-TPR families in two accessions from H1 haplotypes (G6 and G19) and two accessions from H2 haplotypes (G189 and G205). Two panicle developmental stages were considered: “early branching” and “late branching”. (B) Quality control of the panicle sampling. Histograms of expression profiling by qRT-PCR analysis of LHS1 and TAW1 genes known to be expressed in late and early stages of panicle development respectively. (PDF) [file pgen.1009594.s005.pdf]

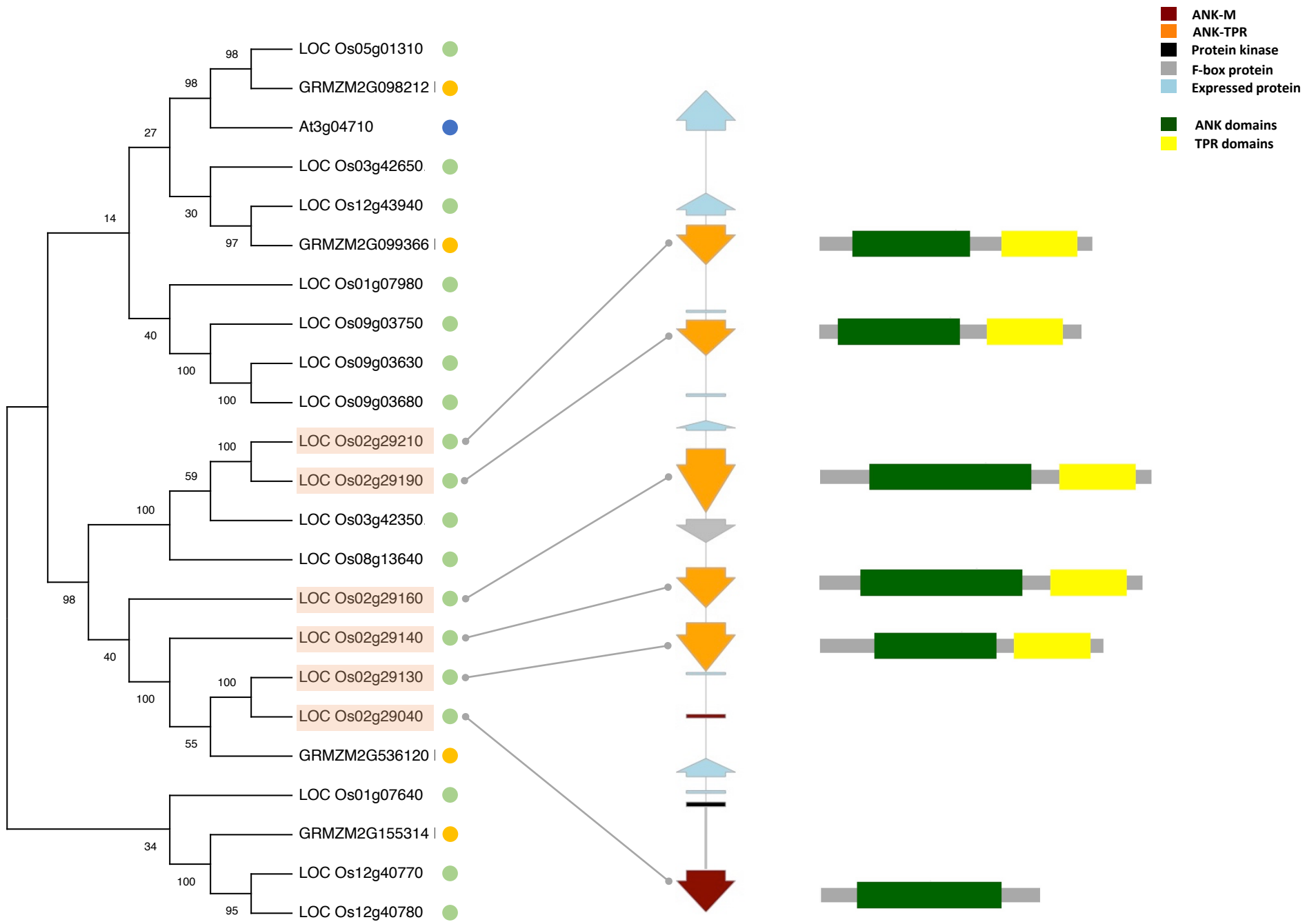

Supplement: S6 Fig — Phylogenetic tree using the Maximum Likelihood method and JTT matrix-based model in conjunction with amino acid sequence alignment of the ANK domain of the ANK-TPR proteins from O. sativa (green dots), Z. mays (yellow dots) and A. thaliana (blue dot). Bootstrap values (1000 tests) are shown next to the branches. Alignment and phylogenetic tree were carried out using MEGA X software [69]. A schematic view of the organization of the ANK gene cluster in QTL_9 based on the O. sativa MSU7.0 reference genome is shown on the right of the tree, the grey lines connecting the corresponding genes between the cladogram and the cluster. The orientation of genes is indicated by arrows. The structure of the 6 ANK and ANK-TPR proteins from the cluster is indicated. (PDF) [file pgen.1009594.s006.pdf]

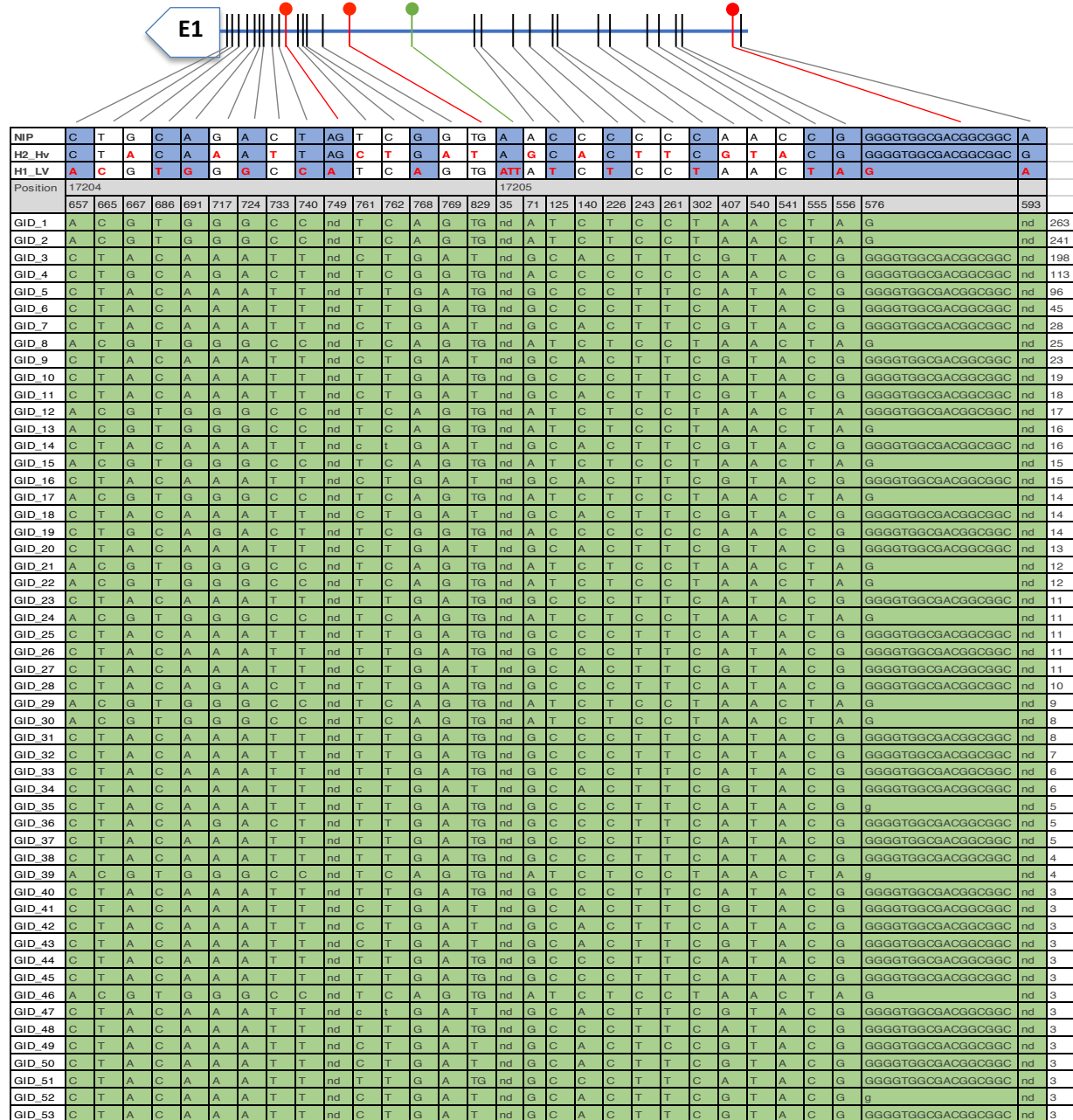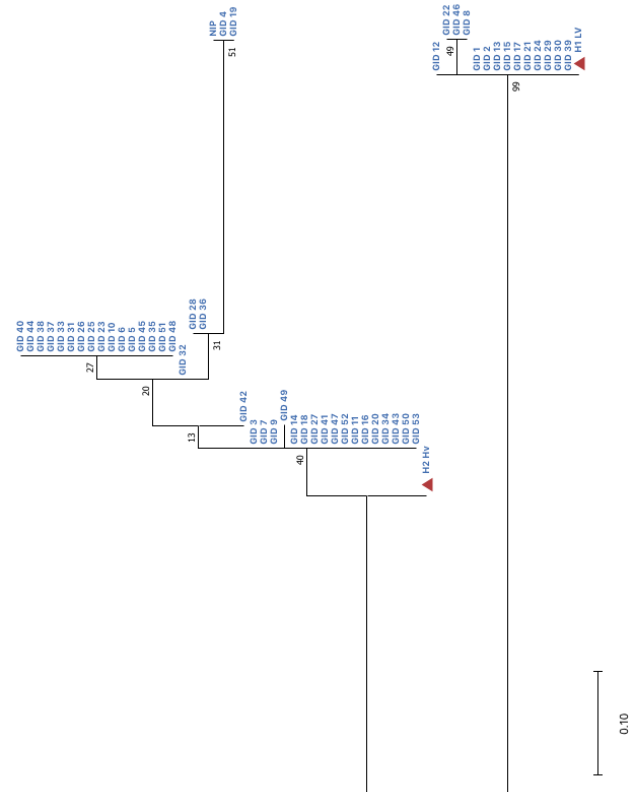

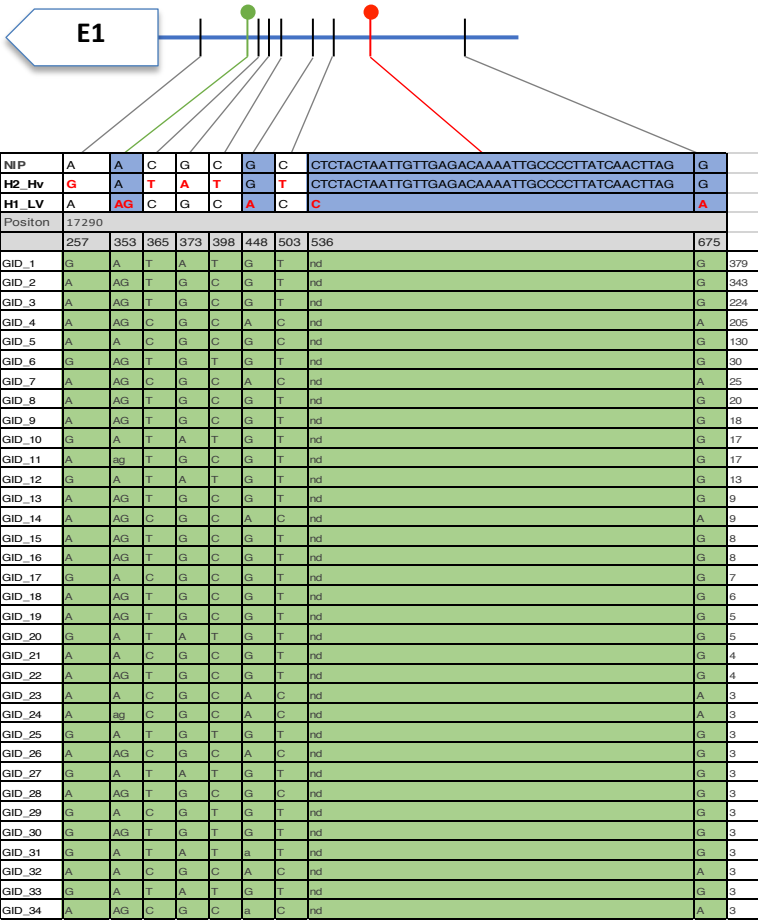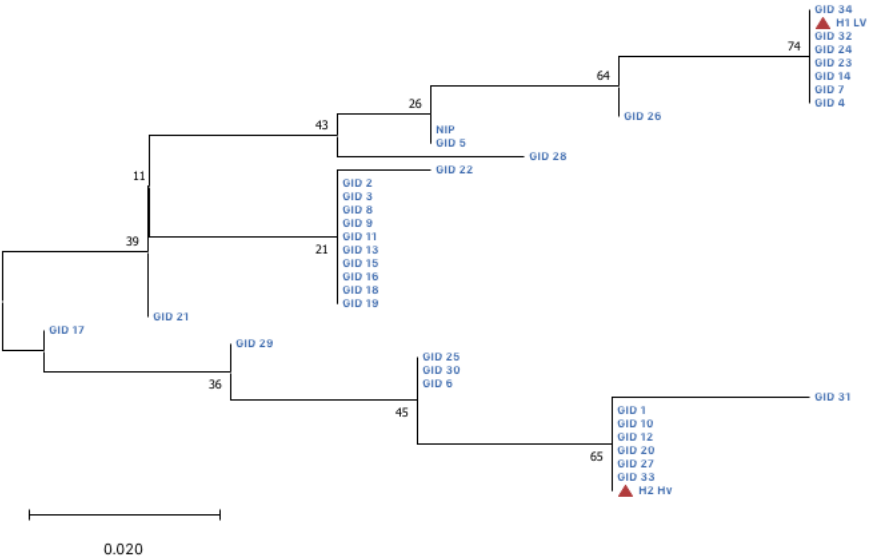

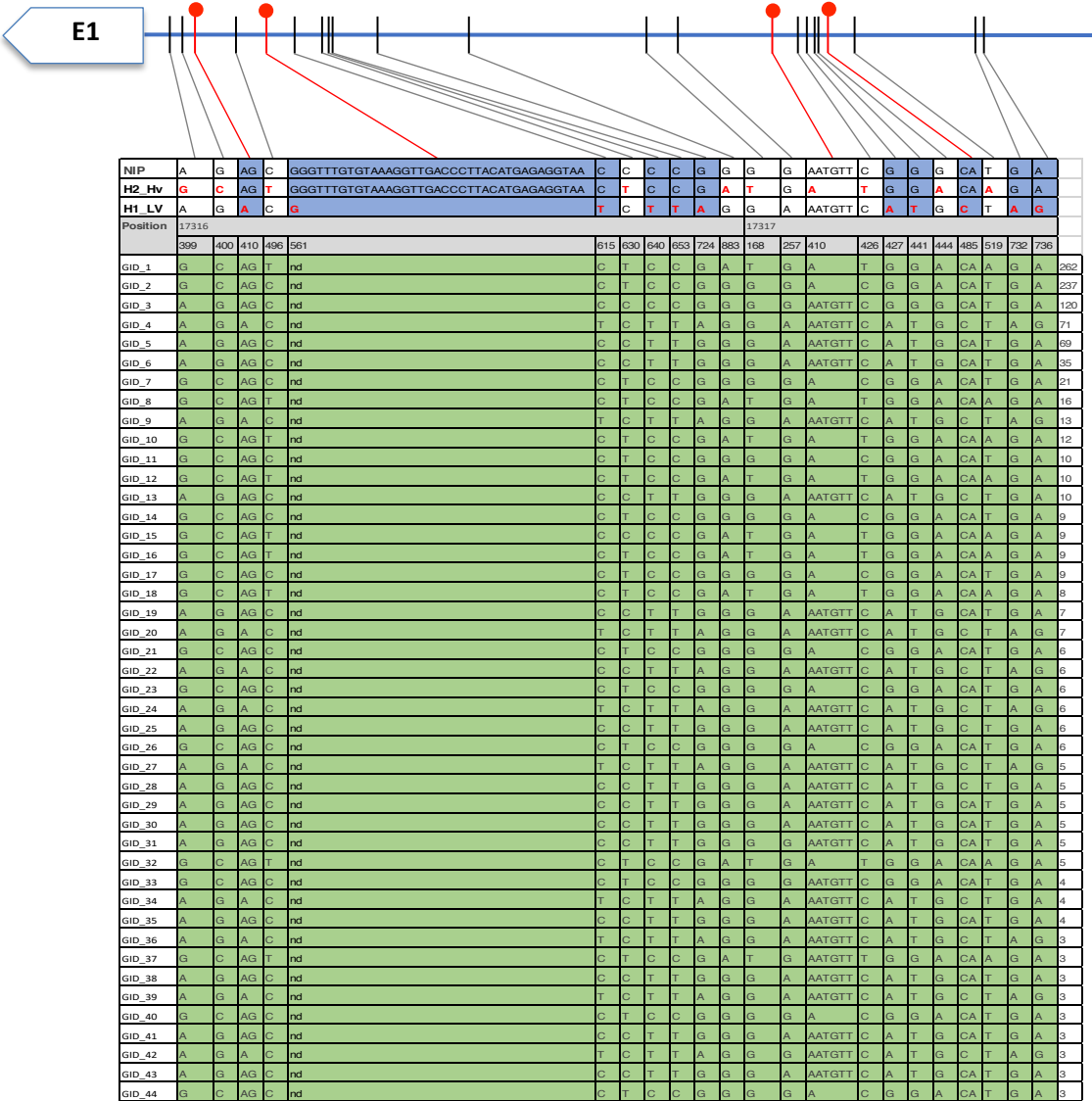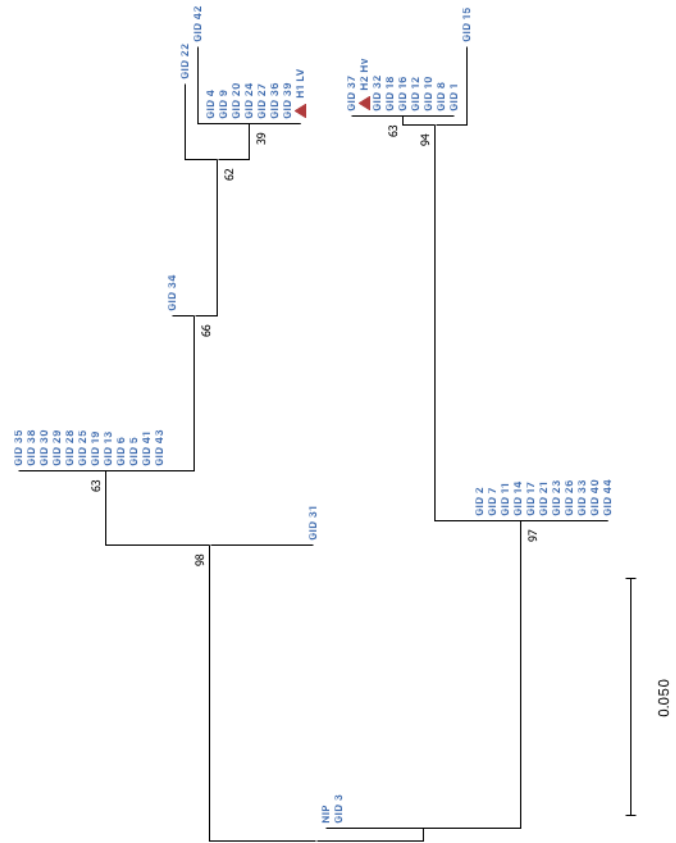

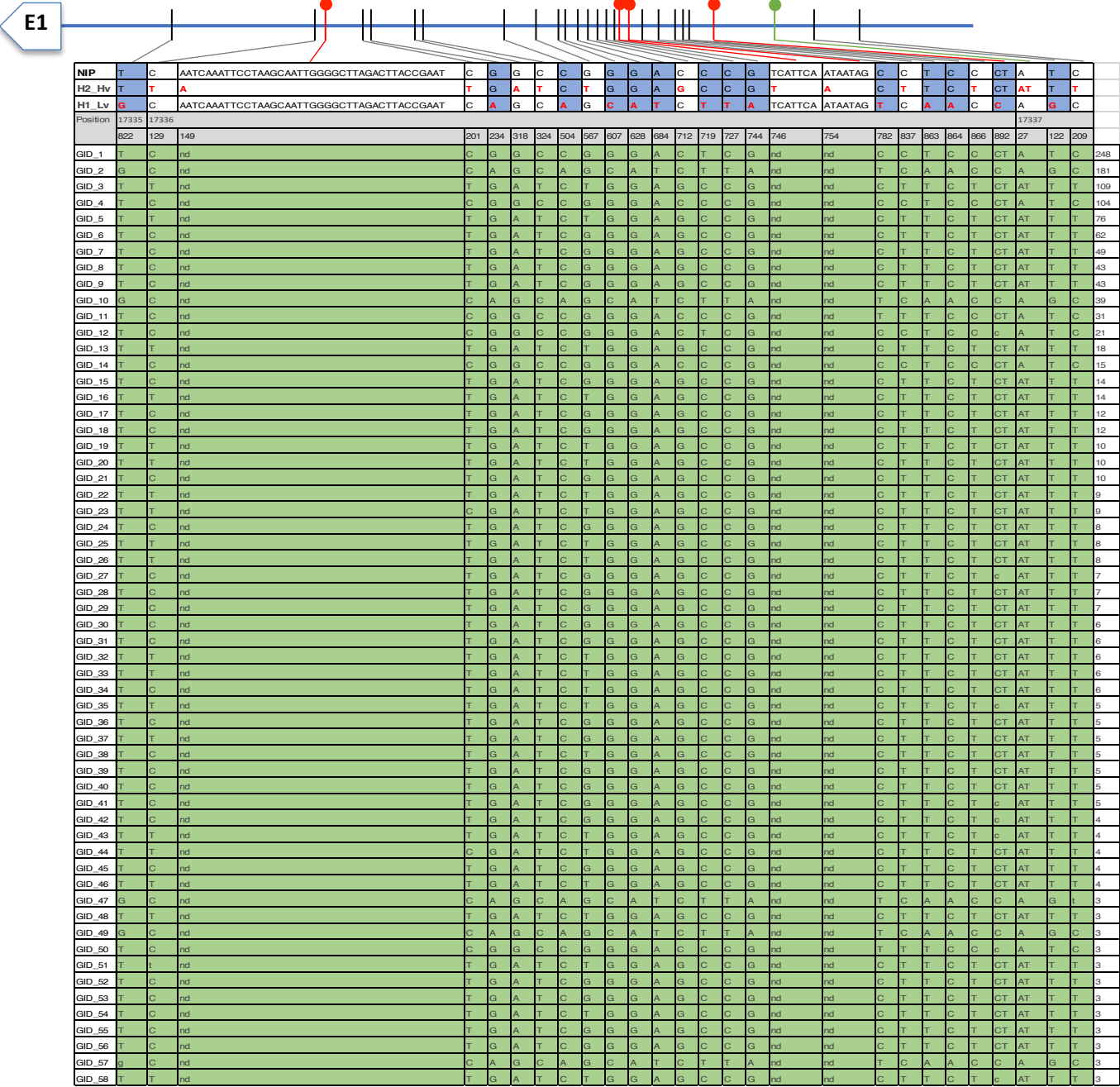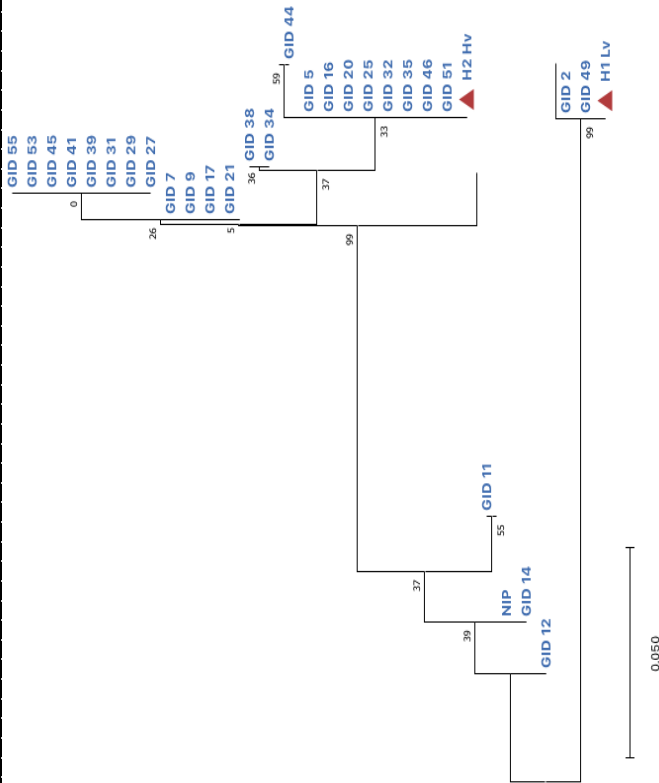

Supplement: S11 Fig — On the left, promoter regions and the first exon (E1) of the 4 ANK and ANK-TPR genes showing differential expression between the haplotypes H1 and H2. SNPs are indicated by vertical black lines. INDELs are indicated by dotted lines: red for a deletion in H1 and green for a deletion in H2 compared to the O. sativa ssp. japonica cv. Nipponbare MSU7.0 reference genome. The sequence and positions of polymorphic sites for the cultivar Nipponbare (Nip) and the H1 and H2 haplotypes (H1_Lv, H2_Hv) are indicated below in comparison with the different haplotypes (GID) from the indica subpopulation according the data available in the MBKbase website facilities (http://www.mbkbase.org/rice). The number of cultivars for each haplotype is indicated on the right of the table. On the right, relationship tree between the different haplotypes using common SNPs in the promoter regions of the ANK and ANK-TPR genes in conjunction with the Neighbor-joining method. (PDF) [file pgen.1009594.s011.pdf]

A

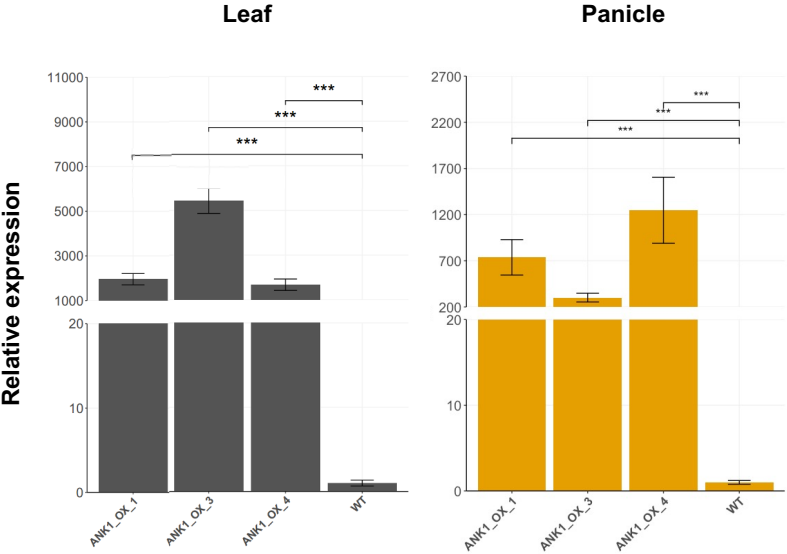

B

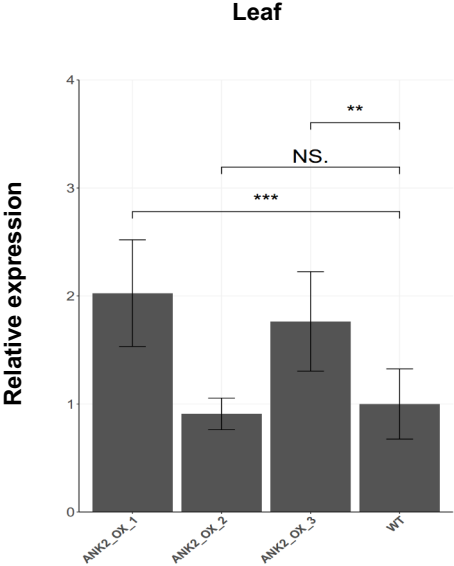

Supplement: S14 Fig — Expression profiling of LOC_Os02g29040 (A) and LOC_Os02g29210 (B) in the corresponding overexpressing transgenic lines in comparison with wild-type Kitaake cultivar in leaf and panicle tissues as indicated. Statistical significance (t test p-values) between the lines and the wild-type is indicated as follows: ns if the test is non-significant, ** if p-values <0.01, *** if <0.001. (PDF) [file pgen.1009594.s014.pdf]

**A**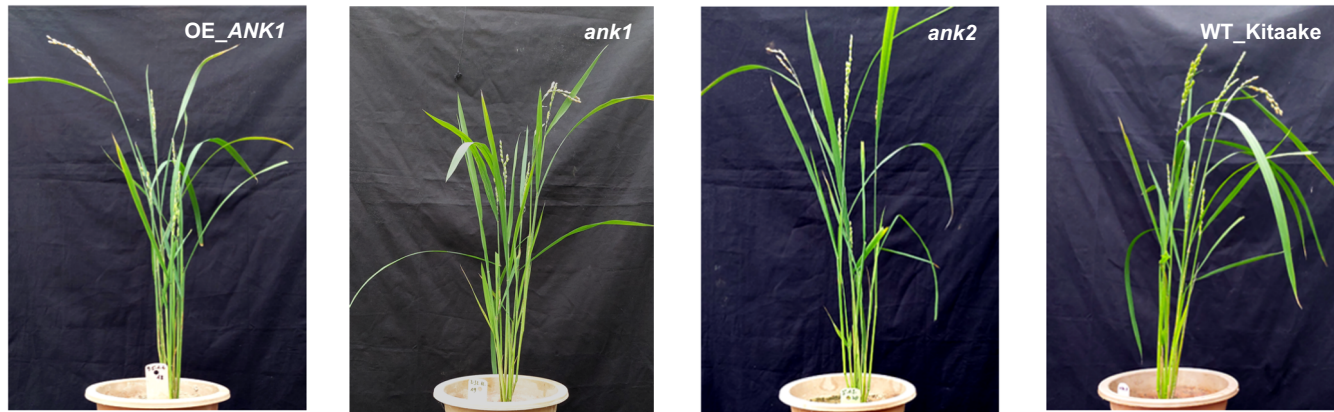**B**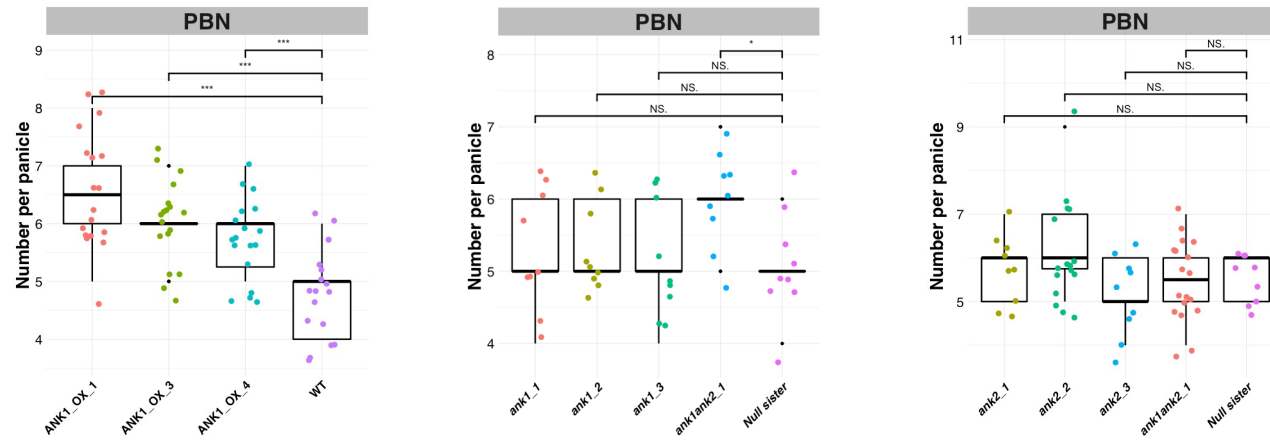**C**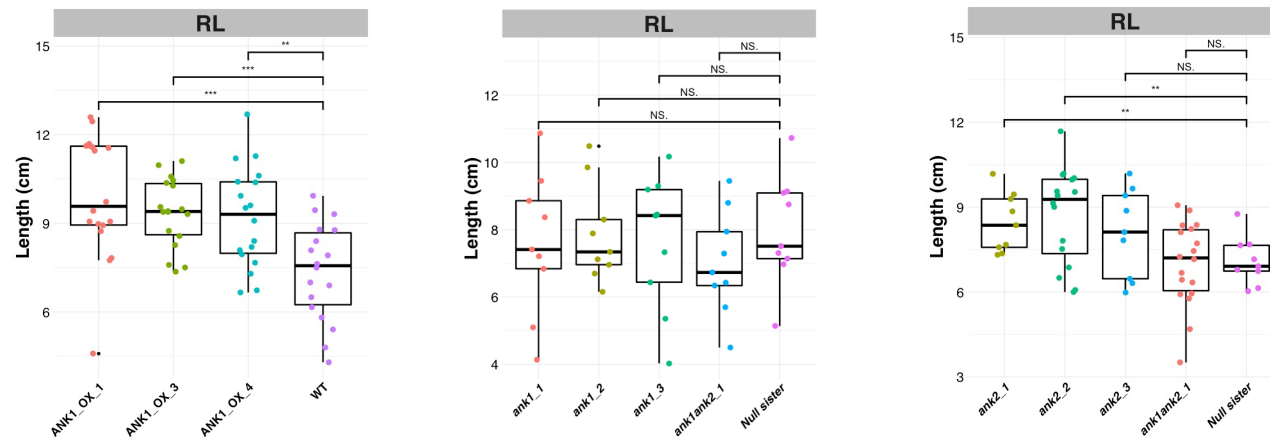

Supplement: S16 Fig — (A) Plant architecture at reproductive stages for an ANK1_OE line (i.e. LOC_Os02g29040 overexpressing line), an ank1 line and an ank2 line (CRISPR-Cas9-derived lines for the LOC_Os02g29040 and LOC_Os02g29210 genes respectively) in comparison with Kitaake wild-type. (B) Box-plots with individual data points showing primary branch number (PBN) per panicle in the different ANK1_OE lines, in the ank1 and ank2 single mutant lines and in the double mutant (ank1ank2_1), in comparison with a “sister” line or the wild-type Kitaake cultivar (WT). (C) Box-plots with individual data points showing rachis length (RL) per panicle in the different ANK1_OE lines, in the ank1 and ank2 single mutant lines and in the double mutant (ank1ank2_1), in comparison with a “sister” line or the wild-type Kitaake cultivar (WT). Statistical significance (i.e. t test p values) between the two lines or parents for the two panicle morphological traits is indicated as follows: NS if the test is non-significant, * if p-values <0.05, ** if <0.01, *** if <0.001. (PDF) [file pgen.1009594.s016.pdf]

**LOC\_Os02g29040**

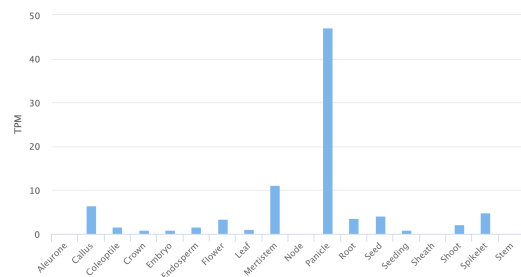

**LOC\_Os02g29130**

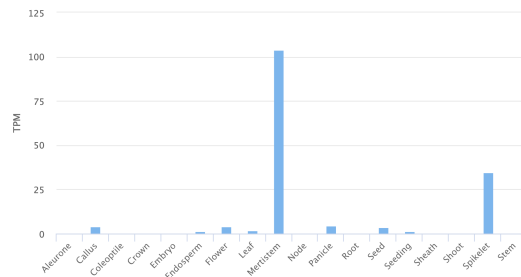

**LOC\_Os02g29140**

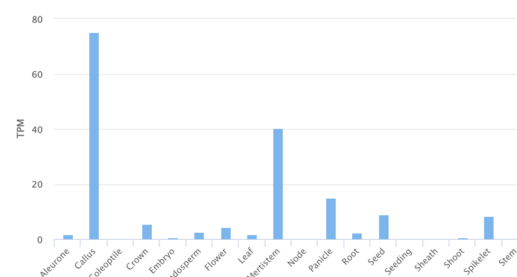

**LOC\_Os02g29160**

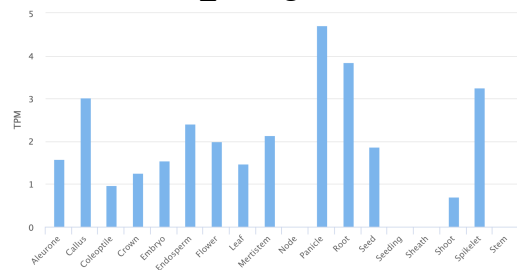

**LOC\_Os02g29190**

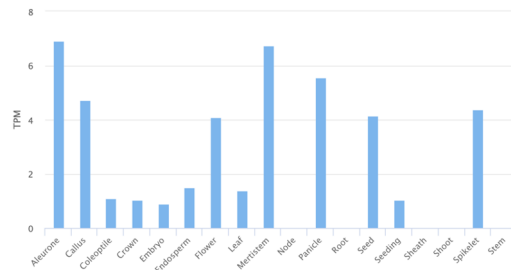

**LOC\_Os02g29210**

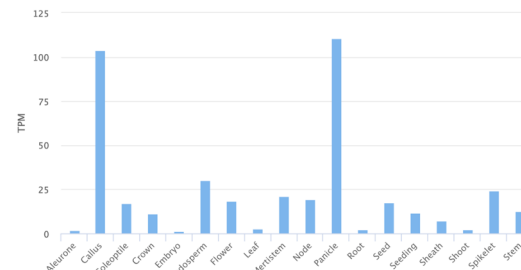

Supplement: S17 Fig — Expression profiles of the ANK genes in various tissues or organs according to data available in IC4R website (http://www.ic4r.org). TPM: transcripts per million. (PDF) [file pgen.1009594.s017.pdf]

**LOC\_Os02g29040**

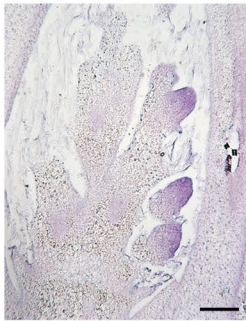

**LOC\_Os02g29160**

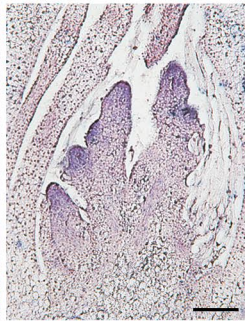

**LOC\_Os02g29210**

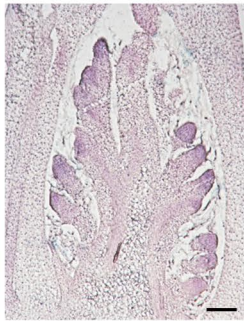

**LOC\_Os02g29210**

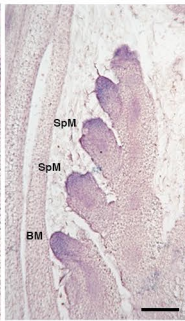

**H4**

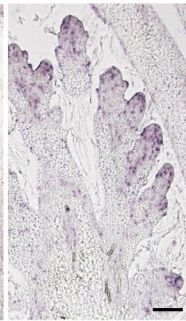

Supplement: S18 Fig — The histone H4 transcripts (H4) were used as a positive control of the specificity of in situ hybridization conditions. SpM: spikelet meristem; BM: branch meristem. Scale: 100 μm. (PDF) [file pgen.1009594.s018.pdf]
