# Supplementary material for: A cluster of Ankyrin and Ankyrin-TPR repeat genes is associated with panicle branching diversity in rice
Source: PLoS Genet. 2021 Jun 7;17(6):e1009594. doi: 10.1371/journal.pgen.1009594 (PMC8211194; doi:10.1371/journal.pgen.1009594)
Supplement: S7 Fig — The structure of the two genes is indicated as defined in MSU7.0 O. sativa reference genome. The sequence of the 3’UTR region of LOC_Os02g29040 is indicated to illustrate the presence of a TPR domain coding sequence downstream the STOP codon. The sequence of the 5’ part of the LOC_Os02g29210 gene is indicated to illustrate the alternative annotation (i.e. without second exon from MSU7.0 reference genome) of this gene in the Nipponbare accession used in the lab. (PDF) [file pgen.1009594.s007.pdf]

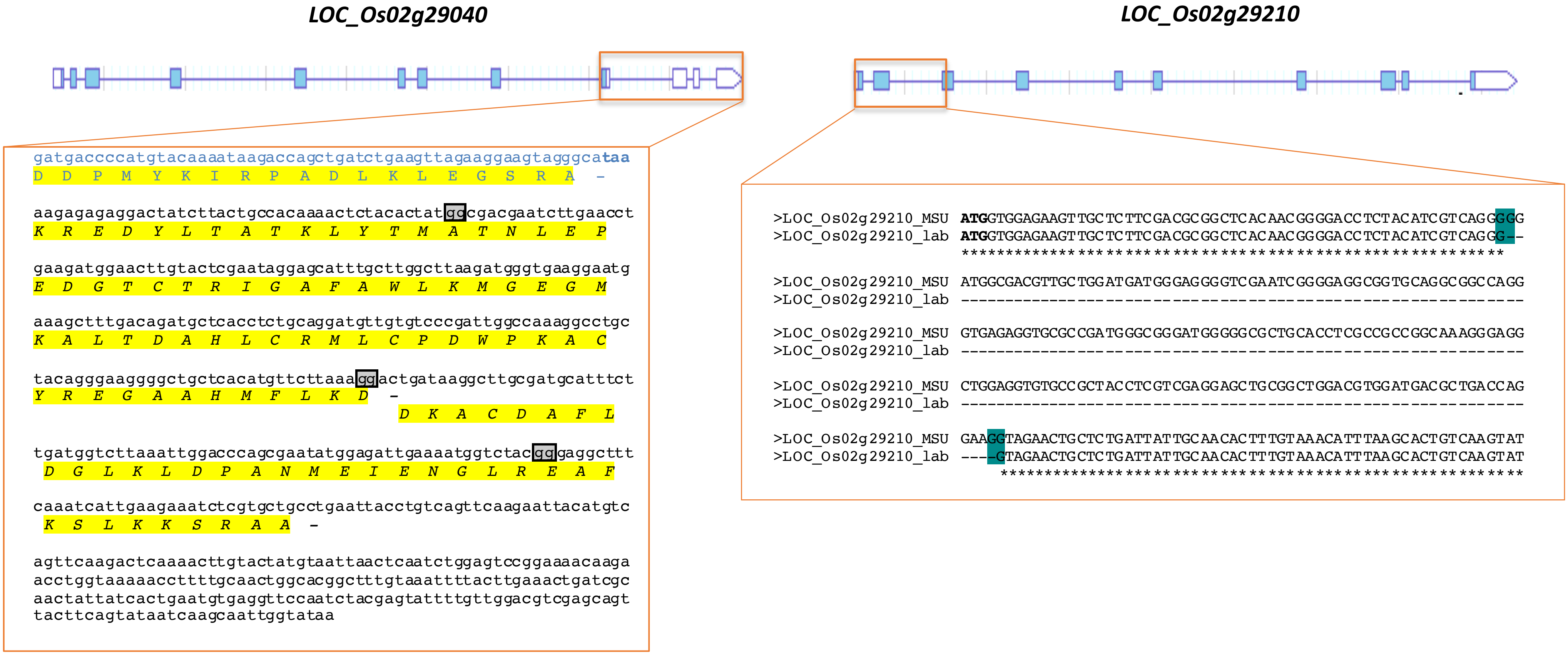
LOC_Os02g29040
gatgaccccatgtacaaaataagaccagctgatctgaagttagaaggaagtagggcataa
D D P M Y K I R P A D L K L E G S R A -
aagagagaggactatcttactgccacaaaactctacactatggcgacgaatcttgaacct
K R E D Y L T A T K L Y T M A T N L E P
gaagatggaacttgtactcgaataggagcatttgcttggcttaagatgggtgaaggaatg
E D G T C T R I G A F A W L K M G E G M
aaagctttgacagatgctcacctctgcaggatgttgtgtcccgattggccaaaggcctgc
K A L T D A H L C R M L C P D W P K A C
tacagggaaggggctgctcacatgttcttaaaggactgataaggcttgcgatgcatttct
Y R E G A A H M F L K D -
D K A C D A F L
tgatggtcttaaattggacccagcgaatatggagattgaaaatggtctacgggaggcttt
D G L K L D P A N M E I E N G L R E A F
caaatcattgaagaaatctcgtgctgcctgaattacctgtcagttcaagaattacatgtc
K S L K K S R A A -
agttcaagactcaaaacttgtactatgtaattaactcaatctggagtccggaaaacaaga
acctggtaaaaaccttttgcaactggcacggctttgtaaattttacttgaaactgatcgc
aactattatcactgaatgtgaggttccaatctacgagtattttgttggacgtcgagcagt
tacttcagtataatcaagcaattggtataa
LOC_Os02g29210
>LOC_Os02g29210_MSU ATGGTGGAGAAGTTGCTCTTCGACGCGGCTCACAACGGGGACCTCTACATCGTCAGGGGG
>LOC_Os02g29210_lab ATGGTGGAGAAGTTGCTCTTCGACGCGGCTCACAACGGGGACCTCTACATCGTCAGGG--
>LOC_Os02g29210_MSU ATGGCGACGTTGCTGGATGATGGGAGGGGTCGAATCGGGGAGGCGGTGCAGGCGGCCAGG
>LOC_Os02g29210_lab --
>LOC_Os02g29210_MSU GTGAGAGGTGCGCCGATGGGCGGGATGGGGGCGCTGCACCTCGCCGCCGGCAAAGGGAGG
>LOC_Os02g29210_lab --
>LOC_Os02g29210_MSU CTGGAGGTGTGCCGCTACCTCGTCGAGGAGCTGCGGCTGGACGTGGATGACGCTGACCAG
>LOC_Os02g29210_lab --
>LOC_Os02g29210_MSU GAAGGTAGAACTGCTCTGATTATTGCAACACTTTGTAAACATTTAAGCACTGTCAAGTAT
>LOC_Os02g29210_lab ----GTAGAACTGCTCTGATTATTGCAACACTTTGTAAACATTTAAGCACTGTCAAGTAT
