# Supplementary material for: A cluster of Ankyrin and Ankyrin-TPR repeat genes is associated with panicle branching diversity in rice
Source: PLoS Genet. 2021 Jun 7;17(6):e1009594. doi: 10.1371/journal.pgen.1009594 (PMC8211194; doi:10.1371/journal.pgen.1009594)
Supplement: S9 Fig — Alignment of the sequences from the O. sativa ssp. japonica cv. Nipponbare MSU7.0 reference genome and deduced sequences from the Vietnamese indica accessions from the H1 and H2 haplotypes. The ANK and TPR domains are highlighted in green and yellow respectively. A space was introduced between the consecutive TPR domains in the alignment. Modified amino acid in H1 or H2 accessions compared to Nipponbare are highlighted in light green. The positions of non-synonymous polymorphic sites are indicated in grey for amino acid changes within the same aliphatic group and in orange for amino acid changes between different aliphatic groups. The non-synonymous substitutions at highly conserved sites in ANK domain are boxed in red. The region missing from the LOC_Os02g29210 encoded protein sequence deduced from the in lab Nipponbare accession (compared with the published Nipponbare reference) is indicated in italics. (PDF) [file pgen.1009594.s009.pdf]

```
LOC_Os02g29040_NIP_H2          MAPPHACSPGAT-QRLLLHAAADGDLRLFKRIASRLDGGEGRLKEAVEAVKD------RGAGALHQAARNGRTAMCVYLV[illegible]E[illegible]QVDINAANESGATPLIYAVLG
LOC_Os02g29040_H1              MAPPHACSPGAT-QRLLLHAGADGELRLFKRIASRLDGGEGRLKEAVEAVKD------RGAGALHQAARNGRTAMCVYLV[illegible]E[illegible]QVDINAANESGATPLIYAVLG
LOC_Os02g29130_NIP_H1_H2       MAPHFVLGPGVP-QQLLIQSAAAGDLPAFKKFARMLDGGKGRLKEAVEAVKN------RGAGALHQAARYGRTAMCAYMV[illegible]E[illegible]QVDIDAAD------------
LOC_Os02g29140_NIP             MAPPLPSDPRAP-TRRLLQAAADGDLAAFKRIAGKLDGGKGRLRETVEGVRD------RGAGALHVSSGRGMLAVCSYLV[illegible]E[illegible]QVDVDAADDSGDTPLAYAVRG
LOC_Os02g29140_H2              MAPPLPSDPRAP-TRRLLQAAADGDLAAFKRIAGKLDGGKGRLRETVEGVRD------RGAGALHVSSGRGMLAVCSYLV[illegible]E[illegible]QVDVDAADDSGDTPLAYAVRG
LOC_Os02g29140_H1              MAPPLPSDPRAP-TRRLLQAAADGDLAAFKRIAGKLDGGKGRLRETVEGVRD------RGAGALHVSSGRGMLAVCSYLV[illegible]E[illegible]QVDVDAADDSGDTPLAYAVRG
LOC_Os02g29160_NIP   MASLSRVPPPARGSRRRPPPSSPVPLSPRQEMLLEAASDGDLGFLKRVVRSLDGGRGRPAEAVEAVRE------CGAGALHLAAGAGKLAVCRYLV[illegible]E[illegible]RVDANAIYDQGETPLAYAVNG
LOC_Os02g29160_H2    MASLSRVPPPARGSRRRPPPSSPVPLSPRQEMLLEAASDGDLGFLKRVVRSLDGGRGRPAEAVEAVRE------CGAGALHLAAGAGKLAVCRYLV[illegible]D[illegible]RVDANAIYDQGETPLAYAVNG
LOC_Os02g29160_H1    MASLSRVPPPAHGSRRRPPPSSPVPLSPRQEMLLEAASDGDLGFLKRVVRSLDGGRGRPAEAVEAVRE------GGAGALHLAAGAGKLAVCRYLV[illegible]E[illegible]RVDANAIYDQGETPLAYAVNG
LOC_Os02g29190_NIP                                                MAMLLVEGRGRLGEAVQAARLRGTGPLDGMGALHIAASKGRLEVCRYLV[illegible]E[illegible]RLDVDDTDQEGRTPLIIAIVF
LOC_Os02g29190_H1                                                 MAMLLVEGRGRLGEAVQAARLRGTGPLDGMGALHIAASKGRLEVCRYLV[illegible]E[illegible]RLDVDDTDQEGRTPLIIAIVF
LOC_Os02g29190_H2                                                 MAMLLVEGRGRLGEAVQAARLRGTGPLDGMGALHIAASKGRLEVCRYLV[illegible]E[illegible]RLDVDDTDQEGRTPLIIAIVF
LOC_Os02g29210_NIP                          MVEKLLFDAAHNGDLYIVRGMATLLDDGRGRIGEAVQAARVRG-APMGGMGALHLAAGKGRLEVCRYLV[illegible]E[illegible]RLDVDDADQEGRTALIIATLC
LOC_Os02g29210_H1                           MVEKLLFDAAHNGDLYIVRGMATLLDDGRGRIGEAVQAARVRG-APMGGMGALHLAAGKGRLEVCRYLV[illegible]E[illegible]RLDVDDADQEGRNALIIATLC
LOC_Os02g29210_H2                           MVEKLLFDAAHNGDLYIVRGMATLLDDGRGRIGEAVQAARVRG-APMGGMGALHLAAGKGRLEVCRYLV[illegible]E[illegible]RLDVDDADQEGRTALIIATLC
                                                                ..  *  *.**  *:*:..:        * **** ::  *    :* *:**:*::* :

LOC_Os02g29040_NIP_H2      GIVYTVSYLLDHGANPDKPNEQGRAPLHLAVEQGNCEILKVLLVKGADVDSSSDCGTPLHIAAVKS[illegible]DGCMKILLDHHADCNKVFSTFYTPLIAALMVRSLKCVKLLIKAGADIKGVGTF
LOC_Os02g29040_H1          GIVYTVSYLLDHGANPDKPNEQGRAPLHLAFEQGNCEILKVLLVKGADVDSSSDCGTPLHIAAVKS[illegible]DGCMKILLDHHADCNKVFSTFYTPLIAALMVRSLKCVKLLIKAGADIKGVGTF
LOC_Os02g29130_NIP_H1_H2   -------------------------------ELGHCEIVKVLLVKGANVDSSSDHGTPLHVAASKS[illegible]DGCMKILLDHHADCNKTFSTVCTPLIAAMMGRSLKCCKLLIEAGADVKGVGTF
LOC_Os02g29140_NIP         RSIDGVKYLLDHGSNPDKPDNKGYTPLHVAAAIKGECEIAKILLSRGAHVDSFSSHGTPLHLSAFCQ[illegible]DGVMKILLDHHADFNKLLKPVFTPLIMALNAGSLKCVVLLLKAGADVKGVGTV
LOC_Os02g29140_H2          RSIDGVKYLLDHGSNPDKPDNKGYTPLHVAAAIKGECEIAKILLSRGAHVDSFSSHGTPLHLSAFCQ[illegible]DGVMKILLDHHADFNKLLKPVFTPLIMALNAGSLKCVVLLLKAGADVKGVGTV
LOC_Os02g29140_H1          RSIDGVKYLLDHGSNPDKPDNKGYTPLHVAAAIKGECEIAKILLSRGAHVDSFSSHGTPLHLSAFCQ[illegible]DGVMKILLDHHADFNKLLKPVFTPLIMALNAGSLKCVVLLLKAGADVKGVGTV
LOC_Os02g29160_NIP         ANVATVRYLLDHGAHPEKADNKGFTPLHFAAAEEGYCNVVELLLAKGAQVDSMSVRGTPLHLAATNG[illegible]HRVVKILLDHNADCNKIVSAVYTPLLVAIYGSSLKCVKLLIKAGADVNGVGNI
LOC_Os02g29160_H2          ANVATVRYLLDHGAHPEKADNKGFTPLHFAAAEEGYCNVVELLLAKGAQVDSMSVRGTPLHLAATNG[illegible]HRVVKILLDHNADCNKIVSAVYTPLLVAIYGSSLKCVKLLIKAGADVNGVGNI
LOC_Os02g29160_H1          ANVATVRYLLDHGAHPEKADNKGFTPLHFAAAEEGYCNVVELLLAKGAQVDSMSVRGTPLHLAATNG[illegible]HRVVKILLDHNADCNKIVSAVYTPLLVAIYGSSLKCVKLLIKAGADVNGVGNI
LOC_Os02g29190_NIP         NHVSTVEYLLDRGADANKASHNGLTPIHFAICLGECGMVQLLLAKGACVDPVAYCGTPLHVAATEG[illegible]DGAMKILLDHNADFNKMVDG-LTPLDTAMDSGELKCINLLIKVGAVVSED---
LOC_Os02g29190_H1          NHVSTVEYLLDRGADANKASHNGLTPIHFAICLGECGMVQLLLAKGACVDPVAYCGTPLHVAATEE[illegible]DGAMKILLDHNADFNKMVDG-LTPLDTAMDSGELKCINLLIKVGAVVSED---
LOC_Os02g29190_H2          NHVSTVEYLLDRGADANKASHNGLTPIHFAICLGECGMVQLLLAKGACVDPVAYCGTPLHVAATEG[illegible]DGAMKILLDHNADFNKMVDG-LTPLDTAMDSGELKCINLLIKVGAVVSED---
LOC_Os02g29210_NIP         KHLSTVKYLLDHGADVNKASHDGRTPLHYATHLGDCGTVQLLLAKGACVDTVANRGTPLHVAASKG[illegible]DGAMKILLDHNADFNKMVDGHLTPLATAITAGELKCVNLLIEAGAVVSGD---
LOC_Os02g29210_H1          KHLSTVKYLLDHGADVNKASHDGRTPLHYATHLGDCGTVQLLLAKGACVDTVANRGTPLHVAASKG[illegible]DGAMKILLDHNADFNKMVDGHLTPLATAITAGELKCVNLLIEAGAVVSGD---
LOC_Os02g29210_H2          KHLSTVKYLLDHGADVNKASHDGRTPLHYATHLGDCGTVQLLLAKGACVDTVANRGTPLHVAASKG[illegible]DGAMKILLDHNADFNKMVDGHLTPLATAITAGELKCVNLLIEAGAVVSGD---
                                                          * *   ::** :** **. :  *****::*    :.  :******:** **  ..   ***  *:    .***   **::.** :.

LOC_Os02g29040_NIP_H2      TPLIAAATEGLTDFYKCLLEA-GADPNVPD------------ENRRKDVEILLPVTSRIPSVYDWSVDGIITYVN--KNVQD-DPMYKIRPADLKLEGSRA
LOC_Os02g29040_H1          TPLIAAATEGLTDFYKCLLEA-GADPNVPD------------ENRRKDVEILLPVTSRIPSVYDWSVDGIITYVN--KNVQD-DPMYKIRPADLKLEGSRA
LOC_Os02g29130_NIP_H1_H2   TPLIVAATEGLTDFYKCLLEG-GADPDVPDKFGFLPIEIAARQNRRKDVEILLPVTSRIPSVHDWSVDGMITYVN--KQVEV-DPFFKIRPADLKLEGNRAYMRKDYLTAAKLYNMAIEH
LOC_Os02g29140_NIP         TPLITAANNGQTDFYKCLLEA-GADPNVPDEFGHLPIELAAYNNRRKDVEILLPVTSRIPSVCDWSVDGVISYVKSCPSVED-DPMYKMSPADMKLAASEAYRRQDYITAMKLYTRLTDI
LOC_Os02g29140_H2          TPLITAANNGQTDFYKCLLEA-GADPNVPDEFGHLPIELAAYNNRRKDVEILLPVTSRIPSVCDWSVDGVISYVKLCPSVED-DPMYKMSPADMKLAASEAYRRQDYITAMKLYTRLTDI
LOC_Os02g29140_H1          TPLITAANNGQTDFYKCLLEA-GADPNVPDEFGHLPIELTAYNNRRKDVEILLPVTSRIPSVCDWSVDGVISYVKSCPSVED-DPMYKMSPADMKLAASEAYRRQDYITAMKLYTRLTDI
LOC_Os02g29160_NIP         TPLIASV--GSTEIMKCLLEA-GADPNVPDEFGRMPIEFAVRCGTLKDVNILFPLTSPMPTVPDWSVRGIIRHVNTLPGQKDYESGLEKEVAGLKLQGVEALKRQDYLAASDLYTKALCL
LOC_Os02g29160_H2          TPLIASV--GSTEIMKCLLEA-GADPNVPDEFGRMPIEFAVRCGTLKDVNILFPLTSPMPTVPDWSVRGIIRHVNTLPGQKDYESGLEKEVAGLKLQGVEALKRQDYLAASDLYTKALCL
LOC_Os02g29160_H1          TPLIASV--GSTEIMKCLLEA-GADPNVPDEFGRMPIEFAVRCGTLKDVNIVFPLTSPMPTVPDWSVRGIIRHVNTLPGQKDYESGLEKEVAGLKLQGVEALKRQDYLAASDLYTKALCL
LOC_Os02g29190_NIP         -RMLTAENSGSTECFNYLMEETGANCNISD-------------------------------------------------------NGEPVNKRKATDLKSLGNKAVEKKDYLSATGFYSKALYL
LOC_Os02g29190_H1          -RMLTAENSGSTECFNYLMEETGANCNISD-------------------------------------------------------NGEPVNKRKATDLKSLGNKAVEKKDYLSATGFYSKALYL
LOC_Os02g29190_H2          -RMLTAENSGSTECFNYLMEETGANCNISD-------------------------------------------------------NGEPVNKRKATDLKSLGNKAVEKKDYLSATGFYSKALYL
LOC_Os02g29210_NIP         -CISTAAKGGSNECN-YSMEETGANRNISD-------------------------------------------------------NGEPVSKRKATELKSLGNKAVEKKDYLSATGFYSKALDL
LOC_Os02g29210_H1          -CISTAAKGGSNECN-YSMEETGANRNISD-------------------------------------------------------NGEPVSKRKATELKSLGNKAVEKKDYLSATGFYSKALDL
LOC_Os02g29210_H2          -CISTAAKGGSNECN-YSMEETGANRNISD-------------------------------------------------------NGEPVSKRKATELKSLGNKAVEKKDYLSATGFYSKALDL
                            : .:   * .:     :*  **: ::.*                                                         :.  :   : :*  . .*

LOC_Os02g29040_NIP_H2
LOC_Os02g29040_H1
LOC_Os02g29130_NIP_H1_H2   DPED MTLYSNTSVCWLKMGKGMNALETAQVCRILRPDW PKGCYREGTAHMFMKDYEKACNAFLDGFKLDPANIEIENALREALKSLKASRAA
LOC_Os02g29140_NIP         CPHD ATLFSNRSLCWLKMGAGVNALQDAQICRLMHSDW SKACYLEGAAQMLLKDFEKACDAFFDGLKLDPASDEIAEALRKSFESLKISHAAKVGIGLPSSQKKIIR
LOC_Os02g29140_H2          CPHD ATLFSNRSLCWLKMGAGVNALQDAQICRLMHSDW SKACYLEGAAQMLLKDFEKACDAFFDGLKLDPASDEIAEALRKSFESLKISHAAKVGIGLPSSQKKIIR
LOC_Os02g29140_H1          CPHD ATPFSNRSLCWLKMGAGVNAFQDAQICRLMHSDW SKACYLEGAAQMLLKDFEKACDAFFDGLKLDPASDEIAEALRKSFESLKISHAAKVGIGLPSSQKKIIR
LOC_Os02g29160_NIP         DFND ATLYSNRSLCFLHMGDGDKAYGDAYTCRMMRPDW PKACYRQGAALMLLKEYQKACDALLDGFKMDPGNSEIENALREAMESLKISDGAKLTT
LOC_Os02g29160_H2          DFND ATLYSNRSLCFLHMGDGDKAYGDAYTCRMMRPDW PKACYRQGAALMLLKEYQKACDAFLDGFKMDPGNSEIENALREAMESLKISDGAKLTT
LOC_Os02g29160_H1          DFND ATLYSNRSLCFLHMGDGDKAYGDAYTCRMMRPDW PKACYRQGAALMLLKEYQKACDALLDGLKMDPGNSEIENALREAMESLKISDGAKLTT
LOC_Os02g29190_NIP         YPDD ATLFSNRSLCWHRMGDGGKALLDAHECRKLRSDW PKAYYRLGAALMLLKDYESACEALYNGFKLDPGNSEIEDAFRYPFCLFWSHYGNMAYDDSFG
LOC_Os02g29190_H1          YPDD ATLFSNRSLCWHRMGDGGKALLDAHECRKLRSDW PKAYYRLGAALMLLKDYESACEALYNGFKLDPGNSEIEDAFRYPFCLFWSHYGNMAYDDSFG
LOC_Os02g29190_H2          YPDD ATLFSNRSLCWHRMGDGGKALLDAHECRKLRSDW PKAYYQLGAALMLLKDYESACEALYNGFKLDPGNSEIEDAFRYPFCLFWSHYGNMAYDDSFG
LOC_Os02g29210_NIP         YPDD ATLFSNRSLCWHHMGNGGKALLDAYECRKLRPDW PKAYYRQGAALMLLKDYESACETLYDGLKLDPGNSEMEDALREALASLKASASTEAR
LOC_Os02g29210_H1          YPDD ATLFSNRSLCWHHMGNGGKALLDAYECRKLRPDW PKAYYRQGAALMLLKDYESACETLYNGLKLDPGNSEMEDALREALASLKASASTEAR
LOC_Os02g29210_H2          YPDD ATLFSNRSLCWHHMGNGGKALLDAYECRKLRPDW PKAYYRQGAALMLLKDYESACETLYDGLKLDPGNSEMEDALREALASLKASANTEAR
```
