# Supplementary material for: A cluster of Ankyrin and Ankyrin-TPR repeat genes is associated with panicle branching diversity in rice
Source: PLoS Genet. 2021 Jun 7;17(6):e1009594. doi: 10.1371/journal.pgen.1009594 (PMC8211194; doi:10.1371/journal.pgen.1009594)
Supplement: S10 Fig — Positions of polymorphic genes are indicated on the schematic view of the gene structure. The sequence and position of polymorphic sites for the cultivar Nipponbare (Nip) and the H1 and H2 haplotypes (H1_Lv, H2_Hv) are indicated below in comparison with the different haplotypes (GID) from the indica subpopulation according the data available in the MBKbase website facilities (http://www.mbkbase.org/rice). The number of cultivars for each haplotype is indicated on the right of the table. (PDF) [file pgen.1009594.s010.pdf]

LOC_Os02g29040

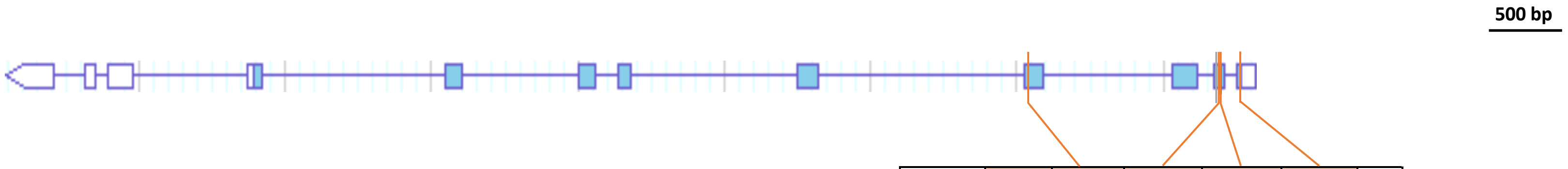

H1_Lv	nd	A	T	C	T	6
	nd	Ttt / F	gaA / E	gGc / G	ccA / P	
H2_Hv	nd	C	G	G	C	7
	nd	Gtt / V	gaC / D	gCc / A	ccG / P	
Position	17200	17203	17204			
	320	60	382	395	521	
NIP	**T**	**C**	**G**	**G**	**C**	1
		Gtt / V	gaC / D	gCc / A	ccG / P	
GID_1	C	C	G	G	C	556
GID_2	C	A	T	C	T	519
GID_3	T	C	G	G	C	99
GID_4	C	C	G	G	C	29

LOC_Os02g29140

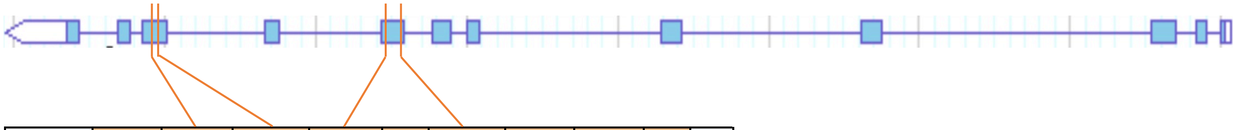

H1_Lv	nd	A	G	G	nd	T	nd	nd	nd	6
	nd	ttT / F	cCa / P	tCg / S	nd	Act / T	nd	nd	nd	
H2_Hv	nd	C	A	A	nd	C	nd	nd	nd	7
	nd	ttG / L	cTa / L	tTg / L	nd	Gct / A	nd	nd	nd	
Position	17258			17260			17261	17265		
	739	929	984	450	515	559	26	614	868	
NIP	**A**	**C**	**A**	**G**	**G**	**C**	**C**	**A**	**T**	1
		ttG / L	cTa / L	tCg / S		Gct / A				
GID_1	G	A	G	G	G	T	C	C	C	330
GID_2	G	C	A	A	G	C	C	C	C	140
GID_3	G	C	A	G	A	C	T	C	C	128
GID_4	A	C	A	G	G	C	C	A	T	114
GID_5	G	A	G	G	G	T	C	C	C	81
GID_6	G	C	A	A	G	C	C	C	C	48
GID_7	G	C	A	G	A	C	T	C	C	30
GID_8	G	C	A	A	G	C	C	C	C	23
GID_9	G	C	A	G	G	C	C	C	C	20
GID_10	G	A	G	G	G	T	C	C	C	13
GID_11	G	A	G	G	G	T	C	C	C	8
GID_12	G	C	A	A	G	C	C	C	C	5
GID_13	G	C	A	A	G	C	C	C	C	4
GID_14	G	C	A	A	G	C	C	C	C	3
GID_15	G	C	A	A	G	C	C	C	C	3

500 bp

LOC_Os02g29160

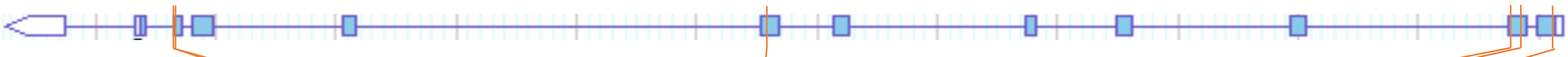

H1_Lv	G	G	nd	nd	nd	C	nd	nd	nd	C	C	nd	nd	T	6
	Ctc / L	Ctc / L	nd	nd	nd	Gta / V	nd	nd	nd	gaG / E	Ggc / G	nd	nd	cAc / H	
H2_Hv	A	A	nd	nd	nd	G	nd	nd	nd	G	A	nd	nd	C	7
	Ttc / F	Ttc / F	nd	nd	nd	Cta / L	nd	nd	nd	gaC / D	Tgc / C	nd	nd	cGc / R	
Position	17278			17280		17283		17287	17288	17289		17290			
	694	706	822	98	123	621	660	987	11	772	843	18	39	97	
NIP	**A**	**G**	**A**	**G**	**A**	**G**	**A**	**A**	**G**	**C**	**A**	**G**	**C**	**C**	1
	Ttc / F	Ctc / L				Cta / L				gaG / E	Tgc / C			cGc / R	
GID_1	A	G	A	G	A	G	A	A	G	C	A	G	C	C	1647
GID_2	A	G	A	A	A	G	A	G	G	C	A	G	A	C	63
GID_3	G	G	A	A	A	C	A	G	G	C	C	C	C	T	24
GID_4	A	G	A	G	c	G	A	A	G	C	A	G	C	C	21
GID_5	A	A	A	A	A	G	A	G	G	G	A	G	A	C	21
GID_6	A	G	A	A	A	G	G	G	A	C	A	G	A	C	15
GID_7	A	G	c	A	A	G	A	G	G	C	A	G	A	C	9
GID_8	A	A	A	A	A	G	A	G	G	C	A	G	A	C	7
GID_9	A	G	A	A	A	G	G	G	A	C	A	G	A	C	6

500 bp

LOC_Os02g29190

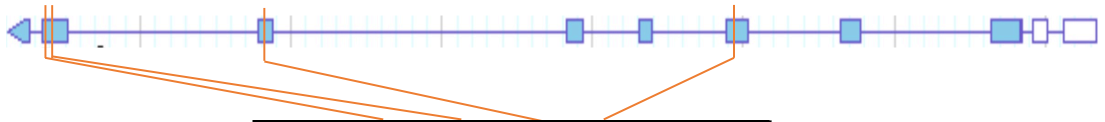

H1_Lv	C	C	C	T	nd	7
	cGg/ R	cgG/ R	gaG/ E	gAg / E	nd	
H2_Hv	T	T	T	C	nd	6
	cAg / Q	cgA/ R	gaA/ E	gGg / G	nd	
Position	17309		17310	17313	17314	
	380	406	820	930	672	
NIP	C	C	C	C	G	1
	cGg/ R	cgG/ R	gaG/ E	gGg / G		
GID_1	T	T	T	C	G	411
GID_2	T	T	C	C	G	409
GID_3	T	T	C	C	G	251
GID_4	C	C	C	T	G	219
GID_5	C	C	C	C	G	118
GID_6	T	T	C	C	T	34
GID_7	C	C	C	T	G	13
GID_8	T	T	T	C	G	3
GID_9	T	T	t	C	G	3
GID_10	T	T	C	C	G	3
GID_11	T	T	T	C	G	3

LOC_Os02g29210

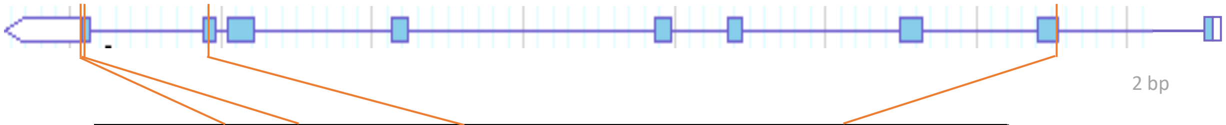

H1_Lv	T	C	nd	nd	T	nd	nd	nd	nd	nd	T	nd	7
	gaA / E	aGc / S	nd	nd	Aat / N	nd	nd	nd	nd	nd	cAt / N	nd	
H2_Hv	C	T	nd	nd	C	nd	nd	nd	nd	nd	G	nd	6
	gaG / E	aAc / N	nd	nd	Gat / D	nd	nd	nd	nd	nd	aCt / T	nd	
Position	17328					17331	17333	17334				17335	
	82	89	99	110	926	979	575	422	456	481	532	549	
NIP	**C**	**C**	**C**	**G**	**C**	**C**	**G**	**A**	**G**	**A**	**G**	**G**	1
	gaG / E	aGc / S			Gat / D						aCt / T		
GID_1	C	T	T	T	C	C	A	A	G	A	G	G	321
GID_2	C	C	T	T	C	C	A	A	G	A	G	G	264
GID_3	T	C	T	T	T	C	A	A	G	A	T	G	194
GID_4	C	C	T	T	C	T	A	C	T	G	G	G	184
GID_5	C	C	C	G	C	C	G	A	G	A	G	G	97
GID_6	C	C	T	T	C	T	A	C	T	G	G	A	15
GID_7	C	C	T	T	C	T	A	C	T	G	G	G	10
GID_8	T	C	T	T	T	C	A	A	G	A	T	G	7
GID_9	T	C	T	T	T	C	A	A	G	A	T	G	4
