# Supplementary material for: A cluster of Ankyrin and Ankyrin-TPR repeat genes is associated with panicle branching diversity in rice
Source: PLoS Genet. 2021 Jun 7;17(6):e1009594. doi: 10.1371/journal.pgen.1009594 (PMC8211194; doi:10.1371/journal.pgen.1009594)
Supplement: S12 Fig — Sequence alignment of the promoter regions of the two haplotypes (H1_Lv, H2_Hv) of the ANK genes with the high-quality full genome sequences from Oryza rufipogon (W1953 accession) and rice cultivars: the two O. sativa ssp. japonica varieties Nipponbare (Nip) and Kitaake (Kita), the two O. sativa ssp. indica varieties R498 and 93–11 and the O. sativa aus variety N22. The positions of putative TFBSs are indicated by blue boxes, along with the names of the corresponding TFs. The positions of MULE and MITES-derived regions in the promoter regions are indicated by colour-filled boxes. Polymorphic sites between H1 and H2 accessions are indicated in orange. Polymorphic sites which are monomorphic for H1 and H2 are indicated in light yellow. (DOCX) [file pgen.1009594.s012.docx]

***LOC_Os02g29040* promoter**

**AP2**

**AP2**

**Indica_H1_Lv** CCGCCGCTTTGGGGGAGGAGAGGGGGGAGGGTGGATCTT----------------CGCCGCCGACGCCGCCCTGCTACTCGCGCTCTCCAGTGTCGACGAAGAAAATGAGGAGAGGAGAGAGATGGGAAAAGGAGGAGAGGAGAGGAGAGAGACCGGAAATAAAGTGAAAAAGAAACTAGTGGGACCCTCTGTAGTTCGT

Indica_R498 CCGCCGCTTTGGGGGAGGAGAGGGGGGAGGGTGGATCT-----------------CGCCGCCGACGCCGCCCTGCTACTCGCGCTCTCCAGTGTCGACGAAGAAAAGGAGGAGAGGAGAGAGATGGGAAAAGGAGGAGAGGAGAGGAGAGAGACCGGAAATAAAGTGAAAAAGAAACTAGTGGGACCCTCTGTAGTTCGT

Indica_9311 CCGCCGCTTTGGGGGAGGAGAGGGGGGAGGGTGGATCT-----------------CGCCGCCGACGCCGCCCTGCTACTCGCGCTCTCCAGTGTCGACGAAGAAAAGGAGGAGAGGAGAGAGATGGGAAAAGGAGGAGAGGAGAGGAGAGAGACCGGAAATAAAGTGAAAAAGAAACTAGTGGGACCCTCTGTAGTTCGT

**Indica_H2_HV** CCGCCGCTTTGGGGGAGGAGAGGGGGGAGGGTGGATCTCGCCGCCGTCGCCACCCCGCCGCCGACGCCGCCCTGCCGCTCGCGCTCTCCATAGTCGACGAAGAAAATGAGGAGAGGAGAGAGATGGGAAAAGGAGGAGAGGAGAGGAGAGAGACCGGAAATAAAGTGAAAAAGAAACTAGTGGGACCCTCTGTAGTTCGT

**Repeat 3**

Japonica_NIP CCGCCGCTTTGGGGGAGGAGAGGGGGGAGGGTGGATCTTGCCGCCGTCGCCACCCCGCCGCCGACGCCGCCCTGCCGCTCGCGCTCTCCAGTGTCGACGAAGAAAATGAGGAGAGGAGAGAGATGGGAAAAGGAGGAGAGGAGAGGAGAGAGACCGGAAATAAAGTGAAAAAGAAACTAGTGGGACCCTCTGTAGTTCGT

Japonica_Kita CCGCCGCTTTGGGGGAGGAGAGGGGGGAGGGTGGATCTTGCCGCCGTCGCCACCCCGCCGCCGACGCCGCCCTGCCGCTCGCGCTCTCCAGTGTCGACGAAGAAAATGAGGAGAGGAGAGAGATGGGAAAAGGAGGAGAGGAGAGGAGAGAGACCGGAAATAAAGTGAAAAAGAAACTAGTGGGACCCTCTGTAGTTCGT

Aus_N22 CCGCCGCTTTGGGGGAGGAGAGGGGGGAGGGTGGATCTCGCCGTCGTCGCCACCCCGCCGCCGACGCCGCCCTGCCGCTCGCGCTCTCCATAGTCGACGAAGAAAAGGAGGAGAGGAGAGAGATGGGAAAAGGAGGAGAGGAGAGGAGAGAGACCGGAAATAAAGTGAAAAAGAAACTAGTGGGACCCTCTGTAGTTCGT

*O. rufipogon* CCGCCGCTTTGGGGGAGGAGAGGGGGGAGGGTGGATCTTGCCGCCGTCGCCACCCCGCCGCCGACGCCGCCCTGCCGCTCGCGCTCTCCAGTGTCGACGAAGAAAATGAGGAGAGGAGAGAGATGGGAAAAGGAGGAGAGGAGAGGAGAGAGACCGGAAATAAAGTGAAAAAGAAACTAGTGGGACCCTCTGTAGTTCGT

************************************** ******************** ************* ************** *********************************************************************************************

**Indica_H1_Lv** AGGTAGGTAACTTGCACTGTGGAGTATGTGGCTAATGGGGTTCTTCATCTAGCCGGCAAACGAATCAGAAAAATCGCGTGGCGCACTGCTATTGCCCTAATGGAGAGTTTAGATGGAGAGGCTGTTGGAACAAGGAATGTGTTTGGCTTGACAAATCAATTGCAGAGCTGGCAATATAGGTTTTTGGAGAGTCCGATTTG

Indica_R498 AGGTAGGTAACTTGCACTGTGGAGTATGTGGCTAATGGGGTTCTTCATCTAGCCGGCAAACGAATCAGAAAAATCGCGTGGCGCACTGCTATTGCCCTAATGGAGAGTTTAGATGGAGAGGCTGTTGGAACAAGGAATGTGTTTGGCTTGACAAATCAATTGCAGAGCTGGCAATATAGGTTTTTGGAGAGTCCGATTTG

Indica_9311 AGGTAGGTAACTTGCACTGTGGAGTATGTGGCTAATGGGGTTCTTCATCTAGCCGGCAAACGAATCAGAAAAATCGCGTGGCGCACTGCTATTGCCCTAATGGAGAGTTTAGATGGAGAGGCTGTTGGAACAAGGAATGTGTTTGGCTTGACAAATCAATTGCAGAGCTGGCAATATAGGTTTTTGGAGAGTCCGATTTG

**Indica_H2_HV** AGGTAGGTAACTTGCACTGTGGAGCATGTGGCTAATGGGGTTCTTCATCTAGCCGGCAAACGAATCAGAAAAATCGCGTGGCGCACTGCTATTGCCCTAATGGAGAGTTTAGATGGAGAGGCTGTTGGAGCAAGGAATGTGTTTGGCTTGACAAATCAATTGCAGAGCTGACAATATAGGTTTTTGGAAAGTCCGATTTG

Japonica_NIP AGGTAGGTAACTTGCACTGTGGAGTATGTGGCTAATGGGGTTCTTCATCTAGCCGGCAAACGAATCAGAAAAATCGCGTGGCGCACTGCTATTGCCCTAATGGAGAGTTTAGATGGAGAGGCTGTTGGAGCAAGGAATGTGTTTGGCTTGACAAATCAATTGCAGAGCTGGCAATATAGGTTTTTGGAGAGTCCGATTTG

**Repeat 2**

Japonica_Kita AGGTAGGTAACTTGCACTGTGGAGTATGTGGCTAATGGGGTTCTTCATCTAGCCGGCAAACGAATCAGAAAAATCGCGTGGCGCACTGCTATTGCCCTAATGGAGAGTTTAGATGGAGAGGCTGTTGGAGCAAGGAATGTGTTTGGCTTGACAAATCAATTGCAGAGCTGGCAATATAGGTTTTTGGAGAGTCCGATTTG

Aus_N22 AGGTAGGTAACTTGCACTGTGGAGTATGTGGCTAATGGGGTTCTTCATCTAGCCGGCAAACGAATCAGAAAAATCGCGTGGCGCACTGCTATTGCCCTAATGGAGAGTTTAGATGGAGAGGCTGTTGGAGCAAGGAATGTGTTTGGCTTGACAAATCA------GAGCTGACAATATAGGTTTTTGGAAAGTCCGATTTG

*O. rufipogon* AGGTAGGTAACTTGCACTGTGGAGTATGTGGCTAATGGGGTTCTTCATCTAGCCGGCAAACGAATCAGAAAAATCGCGTGGCGCACTGCTATTGCCCTAATGGAGAGTTTAGATGGAGAGGCTGTTGGAGCAAGGAATGTGTTTGGCTTGACAAATCAATTGCAGAGCTGGCAATATAGGTTTTTGGAGAGTCCGATTTG

************************ ******************************************************************************************************** **************************** ****** ***************** ***********

**Indica_H1_Lv** AAGAGACTGTTGGAGACACTCTTACGAATCGATGGAAGAAGAGGAGGCTAAGGCCCGGTTGTATTCTTCAACTCCAAACTTCAGTTTTCTCGTTTTTCATTAGCACATTTTTTAAACTATTAAATAATGCCTTCTTTTAAAAGAATTATTATCACATTTGTCAAACTGTTAAATGATGTATTCTTTTTTAAAAAAAAATC

Indica_R498 AAGAGACTGTTGGAGATACTCTTACGAATCGATGGAAGAAGAGGAGGCTAAGGCCCGGTTGTATTCTTCAACTCCAAACTTCAGTTTTCTCGTTTTTCATTAGCACATTTTTTAAACTATTAAATAATGCCTTCTTTTAAAAGAATTATTATCACATTTGTCAAACTGTTAAATGATGTATTCTTTTTTAAAAAAAAATC

Indica_9311 AAGAGACTGTTGGAGATACTCTTACGAATCGATGGAAGAAGAGGAGGCTAAGGCCCGGTTGTATTCTTCAACTCCAAACTTCAGTTTTCTCGTTTTTCATTAGCACATTTTTTAAACTATTAAATAATGCCTTCTTTTAAAAGAATTATTATCACATTTGTCAAACTGTTAAATGATGTATTCTTTTTTAAAAAAAAATC

**Indica_H2_HV** AAGAGGCTGTTGGAGACACTCTTACGAATCGATGGAAGAAGAGGAGGCTAAGGCCCGGTTGTATTCTTCAACTCCAAACTTCAGTTTTCTCTTTTTTCATTAGCACGTTTTTTAAACTATTAAATAATGCCTTCTTTTAAAAGAATTATTATCACATTTGCCAAACTGTTAAATGATGTATTCTTTTTTAAAAAAA--TC

**Repeat 1**

Japonica_NIP AAGAGGCTGTTGGAGACACTCTTACGAATCGATGGAAGAAGAGGAGGCTAAGGCCCGGTTGTATTCTTCAACTCCAAACTTCAGTTTTCTCGTTTTTCATTAGCACGTTTTTTAAACTATTAAATAATGCCTTCTTTTAAAAGAATTATTATCACATTTGTCAAACTGTTAAATGATGTATTCTTTTTTAAAAAAA--TC

Japonica_Kita AAGAGGCTGTTGGAGACACTCTTACGAATCGATGGAAGAAGAGGAGGCTAAGGCCCGGTTGTATTCTTCAACTCCAAACTTCAGTTTTCTCGTTTTTCATTAGCACGTTTTTTAAACTATTAAATAATGCCTTCTTTTAAAAGAATTATTATCACATTTGTCAAACTGTTAAATGATGTATTCTTTTTTAAAAAAA--TC

Aus_N22 AAGAGGCTGTTGGAGATACTCTTACGAATCGATGGAAGAAGAGGAGGCTAAGGCCCGGTTGTATTCTTCAACTCCAAACTTCAGTTTTCTCGTTTTTCATTAGCACGTTTTTTAAACTATTAAATAATGCCTTCTTTTAAAAGAATTATTATCACATTTGCCAAACTGTTAAATGATGTATTCTTTTTTAAAAAAA--TC

*O. rufipogon* AAGAGGCTGTTGGAGATACTCTTACGAATCGATGGAAGAAGAGGAGGCTAAGGCCCGGTTGTATTCTTCAACTCCAAACTTCAGTTTTCTCGTTTTTCATTAGCACGTTTTTTAAACTATTAAATAATGCCTTCTTTTAAAAGAATTATTATCACATTTGTCAAACTGTTAAATGATGTATTCTTTTTTAAAAAAA--TC

***** ********** ************************************************************************** ************** ***************************************************** *********************************** **

**Indica_H1_Lv** TATGTAGTAGTTGTTTTTAAAAAGCAAATAAATCCAATTTTTAAAACTTATAACAATTAAT-------TAA--ATGTTTCGCGTGTAGTACAATTTTTTCTCCAGTTTGAAGAATCGAGCGAAGCCTAAGTGTAATTTTGTCAAAACCGTAAAAGTGACATTTTAACAAAGAAAA-CCCTTTAACCAGATAACGTTTTAG

Indica_R498 TATGTAGTAGTTGTTTTTAAAAAGCAAATAAATCCAATTTTTAAAACTTATAACAATTAATGTTTAATTAATCATGTTTCGCGTGTAGTACAATTTTTTCTCCAGTTTGAAGAATCGAGCGAAGCCTAAGTGTAATTTTGTCAAAACCGTAAAAGTGACATTTTAACAAAGAAAAACCCTTTAACCAGATAACGTTTTAG

Indica_9311 TATGTAGTAGTTGTTTTTAAAAAGCAAATAAATCCAATTTTTAAAACTTATAACAATTAATGTTTAATTAATCATGTTTCGCGTGTAGTACAATTTTTTCTCCAGTTTGAAGAATCGAGCGAAGCCTAAGTGTAATTTTGTCAAAACCGTAAAAGTGACATTTTAACAAAGAAAAACCCTTTAACCAGATAACGTTTTAG

**Indica_H2_HV** TATGTAGTAGTTGTTTTTAAAAAGCAAATAAATCCAATTTTTAAAACTTATAACAATTAAT-------TAA--ATGTTTCGCGTGTAGTACAATTTTTTCTCCAGTTTGAAGAATCGAGCGAAGCCTAAGTGTAATTTTGTCAAAACCGTAAAAGTGACATTTTAACAAAGAAAA-CCCTTTAACCAGATAACGTTTTAG

Japonica_NIP TATGTAGTAGTTGTTTTTAAAAAGCAAATAAATCCAATTTTTAAAACTTATAACAATTAAT-------TAA--ATGTTTCGCGTGTAGTACAATTTTTTCTCCAGTTTGAAGAATCGAGCGAAGCCTAAGTGTAATTTTGTCAAAACCGTAAAAGTGACATTTTAACAAAGAAAA-CCCTTTAACCAGATAACGTTTTAG

Japonica_Kita TATGTAGTAGTTGTTTTTAAAAAGCAAATAAATCCAATTTTTAAAACTTATAACAATTAAT-------TAA--ATGTTTCGCGTGTAGTACAATTTTTTCTCCAGTTTGAAGAATCGAGCGAAGCCTAAGTGTAATTTTGTCAAAACCGTAAAAGTGACATTTTAACAAAGAAAA-CCCTTTAACCAGATAACGTTTTAG

Aus_N22 TATGTAGTAGTTGTTTTTAAAAAGTAAATAAATCCAATTTTTAAAACTTATAACAATTAATATTTAATTAATCATGTTTCGCGTGTAGTACAATTTTTTCTCCAGTTTGAAGAATCGAGCGAAGCCTAAGTGTAATTTTGTCAAAACCGTAAAAGTGACATTTTAACAAAGAAAAACCCTTTAACTAGATAACGTTTTAG

*O. rufipogon* TATGTAGTAGTTGTTTTTAAAAAGCAAATAAATCCAATTTTTAAAACTTATAACAATTAAT-------TAA--ATGTTTCGCGTGTAGTACAATTTTTTCTCCAGTTTGAAGAATCGAGCGAAGCCTAAGTGTAATTTTGTCAAAACCGTAAAAGTGACATTTTAACAAAGAAAA-CCCTTTAACCAGATAACGTTTTAG

************************ ************************************ *** ****************************************************************************************************** ********* **************

**Indica_H1_Lv** CGAAAATAATCCCCATTTTATCGTCCTGTACACTCGTCTGCGTGAGGAGGAGACTCCTCTAAAGTTGTACACTCCTTAGAGGAGACAGAACCC-TGACTTTATGCAGTTTGCAGTTATGCCACTAACATGTGAGCCACAGATCCTCTAACTTCATGTACAGTCTGCAGTTGTGCCACTGAAATGTGTGCGCAGACTGCAC

Indica_R498 CGAAAATAATCCCCATTTTATCGTCCTGTACACTCGTCTGCGTGAGGAGGAGACTCCTCTAAAGTTGTACACTCCTTAGAGGAGACAGAACCC-TGACTTTATGCAGTTTGCAGTTATGCCACTAACATGTGAGCCACAGATCCTCTGACTTCGTGTACAGTCTGCAGTTGTGCCACTGACATGTGTGCGCAGACTGCAC

Indica_9311 CGAAAATAATCCCCATTTTATCGTCCTGTACACTCGTCTGCGTGAGGAGGAGACTCCTCTAAAGTTGTACACTCCTTAGAGGAGACAGAACCC-TGACTTTATGCAGTTTGCAGTTATGCCACTAACATGTGAGCCACAGATCCTCTGACTTCGTGTACAGTCTGCAGTTGTGCCACTGACATGTGTGCGCAGACTGCAC

**Indica_H2_HV** CGAAAATAATCCC-ATTTTATCGTCCTGTACACTCGTCTGCGTGAGGAGGAGACTCCTCTAAAGTTGTACACTCTCTAGAGAGGACAGAACCCCTGACTTTATACAGTTTACAGTTATGTCACTAATATGTGAGCCACAGATCCTCTAACTTTATGTGCAGTCTGCAGTTGTGCCATTAAAATGTGGGCGCAGACTGCAC

Japonica_NIP CGAAAATAATCCCCATTTTATCGTCCTGTACACTCGTCTGCGTGAGGAGGAGACTCCTCTAAAGTTGTACACTCCCTAGAGGAGACAGAACCCCTGACTTTATACAGTTTGCAGTTATGTCACTAACATGTGAGCCACAGATCCTCTAACTTTATGTGCAGTCTGCAGTTGTGCCACTAAAATGTGGGCGCAGACTGCAC

Japonica_Kita CGAAAATAATCCCCATTTTATCGTCCTGTACACTCGTCTGCGTGAGGAGGAGACTCCTCTAAAGTTGTACACTCCCTAGAGGAGACAGAACCCCTGACTTTATACAGTTTGCAGTTATGTCACTAACATGTGAGCCACAGATCCTCTAACTTTATGTGCAGTCTGCAGTTGTGCCACTAAAATGTGGGCGCAGACTGCAC

Aus_N22 CGAAAATAATCCCCATTTTATCGTCCTGTACACTCGTCTGCGTGAGGAGGAGACTCCTCTAAAGTTGTACACTCTCTAGAGAAGACAGAACCCTTGACTTTATACAGTTTACAGTTATGTCACTAATATGTGAGCCACAGATCCTCTGACTTTGTGTGCAGTCTGCAGTTGTGCCATTAACATGTGGGCGCAGACTGCAC

*O. rufipogon* CGAAAATAATCCCCATTTTATCGTCCTGTACACTCGTCTGCGTGAGGAGGAGACTCCTCTAAAGTTGTACACTCCCTAGAGGAGACAGAACCCCTGACTTTATACAGTTTACAGTTATGTCACTAACATGTGAGCCACAGATCCTCTAACTTTATGTGCAGTCTGCAGTTGTGCCACTAAAATGTGGGCGCAGACTGCAC

************* ************************************************************ ***** ********** ********* ****** ******** ****** ******************** **** *** ****************** * * ***** *************

**Indica_H1_Lv** AAAGTGGATCTG**T**

Indica_R498 AAAGTGGATCTG**T**

Indica_9311 AAAGTGGATCTG**T**

**Indica_H2_HV** AAAGTGGATCTG**T**

Japonica_NIP AAAGTGGATCTG**T**

Japonica_Kita AAAGTGGATCTG**T**

Aus_N22 AAAGTGGATCTG**T**

*O. rufipogon* AAAGTGGATCTG**T**

*****************

**NB-YB**

**NB-YB**

**ARF-B3**

***LOC_Os02g29160* promoter**

**Indica_H1_Lv** CATGCAAAGGCCGCCGCGGCGGACGTGCACGATGTTCCCGCGCATGATGTGGAGAGGCGGCGCGCCGGCTGACAGCAGCGGCGTCGCCGCCCCTTCTCTTTGTCTCCTCTCTCACCATGTTCT-----------------------------------------GGAGAGAATCTTTTCGAAAATGAATAAAATGGTGAA

Indica_R498 CATGCGAAGGCCGCCGCGGCGGACGTGCACGATGTTCCCGCGCATGATGTGGAGAGGCGGCGCGCCGGCTGGCAGCAGCGGCGTCGCCGCCCCTTCTCTTTGTCTCCTCTCTCACCGTGTTCT-----------------------------------------GGAGAGAATCTTTTCGAAAATGAATAAAATGGTGAA

Indica_9311 CATGCGAAGGCCGCCGCGGCGGACGTGCACGATGTTCCCGCGCATGATGTGGAGAGGCGGCGCGCCGGCTGGCAGCAGCGGCGTCGCCGCCCCTTCTCTTTGTCTCCTCTCTCACCGTGTTCT-----------------------------------------GGAGAGAATCTTTTCGAAAATGAATAAAATGGTGAA

**Indica_H2_HV** CATGCAAAGGCCGCCGCGGCGGACGCGCACGATGTTCCCGCGCATGATGTGGAGAGGCGGCGCGCCGGCTGACAGCAGCGGCGTCGCCGCCCCTTCTCTTTGTCTCCTCTCTCACCATGTTCTCTAAGTTGATAAGGGGCAATTTTGTCTCAACAATTAGTAGAGGAGAGAATCTTTTCGAAAATGAATAAAATGGTGAA

Japonica_NIP CATGCAAAGGCCGCCGCGGCGGACGCGCACGATGTTCCCGCGCATGATGTGGAGAGGCGGCGCGCCGGCTGACAGCAGCGGCGTCGCCGCCCCTTCTCTTTGTCTCCTCTCTCACCATGTTCTCTAAGTTGATAAGGGGCAATTTTGTCTCAACAATTAGTAGAGGAGAGAATCTTTTCGAAAATGAATAAAATGGTGAA

Japonica_Kita CATGCAAAGGCCGCCGCGGCGGACGCGCACGATGTTCCCGCGCATGATGTGGAGAGGCGGCGCGCCGGCTGACAGCAGCGGCGTCGCCGCCCCTTCTCTTTGTCTCCTCTCTCACCATGTTCTCTAAGTTGATAAGGGGCAATTTTGTCTCAACAATTAGTAGAGGAGAGAATCTTTTCGAAAATGAATAAAATGGTGAA

Aus_N22 CATGCGAAGGCCGCCGCGGCGGACGCGCACGATGTTCCCGCGCATGATGTGGAGAGGCGGCGCGCCGGCTAGCAGCAGCGGCGTCGCCGCCCCTTCTCTTTGTCTCCTCTCTCACCGTGTTCTCTAAGTTGATAAGGGGCAATTTTGTCTCAACAATTAGTAGAGGAGAGAATCTTTTCGAAAATGAATAAAATGGTAAA

**MULE**

*O. rufipogon* CATGCGAAGGCCGCCGCGGCGGACGCGCACGATGTTCCCGCGCATGATGTGGAGAGGCGGCGCGCCGGCTGGCAGCAGCGGCGTCGCCGCCCCTTCTCTTTGTCTCCTCTCTCACCGTGTTCTCTAAGTTGATAAGGGGCAATTTTGTCTCAACAATTAGTAGAGGAGAGAATCTTTTCAAAAATGAATAAAATGGTGAA

***** ******************* ******************************************** ******************************************** ****** *************** ***************** **

**Indica_H1_Lv** AATGGCAAAGTGGCGGTGGACGTACGTTTCATATTGGCATTTTATCGAATCATGTTTTCGGGATGGCATTCTAGCGAAACCGCGTTTTGAACGTGGCGAAATGTCCATTTTCTCATATCCGATGTGACACGCAAAGACTTTGCACCCCTGGATTTAAACACAGCCAAAGTTAACATTTTAAGGGTGGCAAATACTAGTCG

Indica_R498 AATGGCAAAGTGGCGGTGGACGTACGTTTCATATTGGCATTTTATCGAATCATGTTTTCGGGATGGCATTCTAGCGAAACCGCGTTTTGAACGTGGCGAAATGTCCATTTTCTCATATCCGATGTGACACGCAAAGACTTTGCACCCCTGGATTTAAACACAGCCAAAGTTAACATTTTAAGGGTGGCAAATACTAGTCG

Indica_9311 AATGGCAAAGTGGCGGTGGACGTACGTTTCATATTGGCATTTTATCGAATCATGTTTTCGGGATGGCATTCTAGCGAAACCGCGTTTTGAACGTGGCGAAATGTCCATTTTCTCATATCCGATGTGACACGCAAAGACTTTGCACCCCTGGATTTAAACACAGCCAAAGTTAACATTTTAAGGGTGGCAAATACTAGTCG

**Indica_H2_HV** AATGGCAAAGTGGCGGTGGACGTACGTTTCATATTGGCATTTTATCGAATCACGTTTTCGGGATGGCATTCTAGCGAAACCGCGTTTTGAACGTGGCGAAATATCCATTTTCTCATATCCGATGTGATACGCAAAAACTTTGCACCC-TGGATTTAAACACAGCCAAAGTTAACATTTTAAGGGTGGCAAATACTAGTCG

Japonica_NIP AATGGCAAAGTGGCGGTGGACGTACGTTTCATATTGGCATTTTATCGAATCACGTTTTCGGGATGGCATTCTAGCGAAACCGCGTTTTGAACGTGGCGAAATGTCCATTTTCTCATATCCGATGTGACACGCAAAGACTTTGCACCC-TGGATTTAAACACAGCCAAAGTTAACATTTTAAGGGTGGCAAATACTAGTCG

Japonica_Kita AATGGCAAAGTGGCGGTGGACGTACGTTTCATATTGGCATTTTATCGAATCACGTTTTCGGGATGGCATTCTAGCGAAACCGCGTTTTGAACGTGGCGAAATGTCCATTTTCTCATATCCGATGTGACACGCAAAGACTTTGCACCC-TGGATTTAAACACAGCCAAAGTTAACATTTTAAGGGTGGCAAATACTAGTCG

Aus_N22 AATGGCAAAGTGGCGGTGGACGTACGTTTCATATTGGCATTTTATCGAATCACGTTTTCGGGATGGCATTCTAGCGAAACCGCGTTTTGAACGTGGCGAAATGTCTATTTTCTCATATCCGATGTGACACGCAAAAACTTTGCACCCTTGGATTTAAACACAGCCAAAGTTAACATTTTAAGGGTGGCAAATACTAGTCG

*O. rufipogon* AATGGCAAAGTGGCGGTGGACGTACGTTTCATATTGGCATTTTATCGAATCACGTTTTCGGGATGGCATTCTAGCGAAACCGCGTTTTGAACGTGGCGAAATGTCCATTTTCTCATATCCGATGTGACACGCAAAGACTTTGCACCCCTGGATTTAAACACAGCCAAAGTTAACATTTTAAGGGTGGCAAATACTAGTCG

**************************************************** ************************************************* ** ********************* ******* *********** ****************************************************

**Indica_H1_Lv** AAAACCCCGTAGCATAAGGGAGTATTCCTGCTGCTGTAGTTAGGTAGGCAGGCAGTAGCAGTGTCGCACATTCAAATCCAAGTTCCTACTCCCCAATCGAT**C**

Indica_R498 AAAACCCCGTAGCATAAGGGAGTATTCCTGCTGCTGTAGTTAGGTAGGCAGGCAGTAGCAGTGTCGCACATTCAAATCCAAGTTCCTACTCCCCAATCGAT**C**

Indica_9311 AAAACCCCGTAGCATAAGGGAGTATTCCTGCTGCTGTAGTTAGGTAGGCAGGCAGTAGCAGTGTCGCACATTCAAATCCAAGTTCCTACTCCCCAATCGAT**C**

**Indica_H2_HV** AAAACCCCGTAGCATAAGGGAGTATTCCTGCTGCTGTAGTTAGGCAGGCAGGCAGTAGCAGTGTCGCACATTCAAATCCAAGTTCCTACTCCCCAATCGAT**C**

Japonica_NIP AAAACCCCGTAGCATAAGGGAGTATTCCTGCTGCTGTAGTTAGGTAGGCAGGCAGTAGCAGTGTCGCACATTCAAATCCAAGTTCCTACTCCCCAATCGAT**C**

Japonica_Kita AAAACCCCGTAGCATAAGGGAGTATTCCTGCTGCTGTAGTTAGGTAGGCAGGCAGTAGCAGTGTCGCACATTCAAATCCAAGTTCCTACTCCCCAATCGAT**C**

Aus_N22 AAAACCCCGTAGCATAAGGGAGTATTCCTGCTGCTGTAGTTAGGTAGGCAGGCAGTAGCAGTGTCGCACATTCAAATCCAAGTTCCTACTCCCCAATCGAT**C**

*O. rufipogon* AAAACCCCGTAGCATAAGGGAGTATTCCTGCTGCTGTAGTTAGGTAGGCAGGCAGTAGCAGTGTCGCACATTCAAATCCAAGTTCCTACTCCCCAATCGATC

******************************************** *************************************************************

***LOC_Os02g29190* promoter**

**Indica_H1_Lv** GAGGCCGTTTGAGATACTCTAACAGCATATGGAAATAGCGATGACTTTTCACGACATACGGCTAGAATCTTTCTTGTTATTTAATACTCCCTCCGTCCCTAGATATTTGACGCTGTTGACTTTTTTAAACATGTTTGACGGTTCGTCTTATTCAAAAACTTTTGTGAAATATGTAAAATTTTATGTATACATAAAAGTAT

Indica_R498 GAGGCCGTTTGAGATACTCTAACAGCATATGGAAATAGCGATGACTTTTCACGGCATACGGCTAGAATCTTTCTTGTTATTTAATACTCCCTCCGTCCCTAGATATTTGACGCTGTTGACTTTTTTAAACATGTTTGACGGTTCGTCTTATTCAAAAACTTTTGTGAAATATGTAAAATTATATGTATACATAAAAGTAT

**MITE-harbinger**

Indica_9311 GAGGCCGTTTGAGATACTCTAACAGCATATGGAAATAGCGATGACTTTTCACGGCATACGGCTAGAATCTTTCTTGTTATTTAATACTCCCTCCGTCCCTAGATATTTGACGCTGTTGACTTTTTTAAACATGTTTGACGGTTCGTCTTATTCAAAAACTTTTGTGAAATATGTAAAATTATATGTATACATAAAAGTAT

**Indica_H2_HV** GAGGCTGTTCGAGATACTCTAACAGCATATGGAAATAGCGATGACTTTTCACGACATACGGCTAGAATCTTTCTTGTTATTTAATACTCCCTCCGTCCCTAGATATTTGACGCTGTTGACTTTTTTAAACATGTTTGACGGTTCGTCTTATTCAAAAACTTTTGTGAAATATGTAAAATTTTATGTATACATAAAAGTAT

Japonica_NIP GAGGCTGTTCGAGATACTCTAACAGCATATGGAAATAGCGATGACTTTTCACGACATACGGCTAGAATCTTTCTTGTTATTTAATACTCCCTCCGTCCCTAGATATTTGACGCTGTTGACTTTTTTAAACATGTTTGACGGTTCGTCTTATTCAAAAACTTTTGTGAAATATGTAAAATTTTATGTATACATAAAAGTAT

Japonica_Kita GAGGCTGTTCGAGATACTCTAACAGCATATGGAAATAGCGATGACTTTTCACGACATACGGCTAGAATCTTTCTTGTTATTTAATACTCCCTCCGTCCCTAGATATTTGACGCTGTTGACTTTTTTAAACATGTTTGACGGTTCGTCTTATTCAAAAACTTTTGTGAAATATGTAAAATTTTATGTATACATAAAAGTAT

Aus_N22 GAGGCTGTTCGAGATACTCTAACAGCATATGGAAATAGCGATGACTTTTCACGGCATACGGCTAGAATCTTTCTTGTTATTTAATACTCCCTCCGTCCCTAGATATTTGACGCTGTTGACTTTTTTAAACATGTTTGACGGTTCGTCTTATTCAAAAACTTTTGTGAAATATGTAAAATTATATGTATACATAAAAGTAT

*O. rufipogon* GAGGCTGTTCGAGATACTCTAACAGCATATGGAAATAGCGATGACTTTTCACGGCATACGGCTAGAATCTTTCTTGTTATTTAATACTCCCTCCGTCCCTAGATATTTGACGCTGTTGACTTTTTTAAACATGTTTGACGGTTCGTCTTATTCAAAAACTTTTGTGAAATATGTAAAATTATATGTATACATAAAAGTAT

***** *** ******************************************* ****************************************************************************************************************************** *******************

**Indica_H1_Lv** ATTTAACAATGAATCAAATGATAGGAAAAGAATTAATAATTACTTAAATTTTTTT-GAATAAAATGATCGGTCAAACATGTTTAAAAAAATCAACAACGTAAAATATTTAGGGATGAAGGGAGTATAACATTTCTTTTTGTTTTGGATACAGGTGGAAATGAAGCTTAGTATTTTTTT--ATACTTCAGTCCTAAAATGG

Indica_R498 ATTTAACAATGAATCAAATGATAGGAAAAGAATTAATAATTACTTAAATTTTTTT-GAATAAAATGATCGGTCAAACATGTTTAAAAAAATCAACAACGTAAAATATTTAGGGATGAAGGGAGTATAACATTTCTTTTTGTTTTGGATACAGGTGGAAATGAAGCTTAGTATTTTTTT--ATACTTCAGTCCTAAAATGG

Indica_9311 ATTTAACAATGAATCAAATGATAGGAAAAGAATTAATAATTACTTAAATTTTTTT-GAATAAAATGATCGGTCAAACATGTTTAAAAAAATCAACAACGTAAAATATTTAGGGATGAAGGGAGTATAACATTTCTTTTTGTTTTGGATACAGGTGGAAATGAAGCTTAGTATTTTTTT--ATACTTCAGTCCTAAAATGG

**Indica_H2_HV** ATTTAACAATGAATCAAATGATTGGAAAAGAATTAATAATTACTTAAATTTTTTTTGAATAAAATGATCGGTCAAACATGTTTAAAAAAATCAACAATGTCAAATATTTAGGGACGAAGGGAGTAT-----TTCTTTTTGTTTTGGATACAGGTGGAAATGAAGCTTAGTATTTTTTT--ATACTTCAGTCCTAAAATGG

Japonica_NIP ATTTAACAATGAATCAAATGATAGGAAAAGAATTAATAATTACTTAAATTTTTTTTGAATAAAATGATCGGTCAAACATGTTTAAAAAAATCAACAACGTCAAATATTTAGGGACGAAGGGAGTATAACATTTCTTTTTGTTTTGGATACAGGTGGAAATGAAGCTTAGTATTTTTTT--ATACTTCAGTCCTAAAATGG

Japonica_Kita ATTTAACAATGAATCAAATGATAGGAAAAGAATTAATAATTACTTAAATTTTTTTTGAATAAAATGATCGGTCAAACATGTTTAAAAAAATCAACAACGTCAAATATTTAGGGACGAAGGGAGTATAACATTTCTTTTTGTTTTGGATACAGGTGGAAATGAAGCTTAGTATTTTTTT--ATACTTCAGTCCTAAAATGG

Aus_N22 ATTTAACAATGAATCAAATGATAGGAAAAGAATTAATAATTACTTAAATTTTTTTTGAATAAAATGATCGGTCAAACATGTTTAAAAAAATCAACAACGTAAAATATTTAGGGATGAAGGGAGTATAACATTTCTTTTTGTTTTGGATACAGGTGGAAATGAAGCTTAGTATTTTTTTT-ATACTTCAGTCCTAAAATGG

*O. rufipogon* ATTTAACAATGAATCAAATGATAGGAAAAGAATTAATAATTACTTAAATTTTTTT-GAATAAAATGATCGGTCAAACATGTTTAAAAAAATCAACAACGTCAAATATTTAGGGACGAAGGGAGTATAACATTTCTTTTTGTTTTGGATACAGGTGGAAATGAAGCTTAGTATTTTTTTTTATACTTCAGTCCTAAAATGG

********************** ******************************** ***************************************** ** ************* *********** *********************************************** ********************

**Indica_H1_Lv** AAATATAAACAATGGGTGCTATATTTTTACAATCATTTTACATGGAAAAAAAAACCGACGTAGCTTTCTCTCAAGACTTATTTTGATACAACCAACAGAGATTTTTAAACCAACATACTTCCTCAATCCTATGATTCCTAACTAAGGAAGTATCTTTTACTTTTTTTTGTTAGTCCTATAATATGTTGATTAAATTTAAT

Indica_R498 AAATATAAACAATGGGTGCTATATTTTTACAATCATTTTACATGGAAAAAAAA-CCGACGTAGCTTTCTCTCAAGACTTATTTTGATACAACCAACAGAGATTTTTAAACCAACATACTTCCTCAATCCTATGATTCCTAACTAAGGAAGTATCTTTTACTTTTTTTTGTTAGTCCTATAATATGTTGATTAAATTTAAT

Indica_9311 AAATATAAACAATGGGTGCTATATTTTTACAATCATTTTACATGGAAAAAAAA-CCGACGTAGCTTTCTCTCAAGACTTATTTTGATACAACCAACAGAGATTTTTAAACCAACATACTTCCTCAATCCTATGATTCCTAACTAAGGAAGTATCTTTTACTTTTTTTTGTTAGTCCTATAATATGTTGATTAAATTTAAT

**Indica_H2_HV** AAATATAAACAATGGGTGCTATATTTTTACAATCATTTTACATGGAAAAAAAAACCGACGTAGCTTTCTCTCAAGACTTATTTTGACACAACCAACAGAGATTTTTAAACCAACATACTTCCTCAATCCTATGATTCCTAACTAAGGAAGTATCTTTTACTTTTTTTTGTTAGTCATATAATATGTTGATTAAATTTAAT

Japonica_NIP AAATATAAACAATGGGTGCTATATTTTTACAATCATTTTACATGGAAAAAAAAACCGACGTAGCTTTCTCTCAAGACTTATTTTGACACAACCAACAGAGATTTTTAAACCAACATACTTCCTCAATCCTATGATTCCTAACTAAGGAAGTATCTTTTACTTTTTTTTGTTAGTCCTATAATATGTTGATTAAATTTAAT

Japonica_Kita AAATATAAACAATGGGTGCTATATTTTTACAATCATTTTACATGGAAAAAAAAACCGACGTAGCTTTCTCTCAAGACTTATTTTGACACAACCAACAGAGATTTTTAAACCAACATACTTCCTCAATCCTATGATTCCTAACTAAGGAAGTATCTTTTACTTTTTTTTGTTAGTCCTATAATATGTTGATTAAATTTAAT

Aus_N22 AAATATAAACAATGGGTGCTATATTTTTACAATCATTTTACATGGAAAAAAAA-CCGACGTAGCTTTCTCTCAAGACTTATTTTGATACAACCAACAGAGATTTTTAAACCAACATACTTCCTCAATCCTATGATTCCTAACTAAGGAAGTATCTTTTACTTTTTTT-GTTAGTCCTATAATATGTTGATTAAATTTAAT

*O. rufipogon* AAATATAAACAATGGGTGCTATATTTTTACAATCATTTTACATGGAAAAAAAA-CCGACGTAGCTTTCTCTCAAGACTTATTTTGACACAACCAACAGAGATTTTTAAACCAACATACTTCCTCAATCCTATGATTCCTAACTAAGGAAGTATCTTTTACTTTTTTTTGTTAGTCCTATAATATGTTGATTAAATTTAAT

***************************************************** ******************************** ******************************************************************************** ******* ************************

**Indica_H1_Lv** TTCTAGACATCTTATGTTTTTATCCCCACAATAGATTTGTTTTTATTGTCGTGACAAGTTACATTTTTTTAATTTACTTCTATAATATATATAAATTACTTTTAGGCTTTATTGAATTTAGTTTTTATGTCTAAGAAGTAATTTAATGAAATTTAAAAGTAATTTAAATATATTATAAAAGTAACTTGTAATTTTTTTAA

Indica_R498 TTCTAGACATCTTATGTTTTTATCCCCACAATAGATTTGTTTTTATTGTCGTGACAAGTTACATTTTTTTAAGTTACTTCTATAATATATATAAATTACTTTTAGGCTTTATTGAATTTAGTTTTTATGTCTAAGAAGTAATTTAATAAAATTTAAAAGTAATTTAAATATATTATAAAAGTAACTTGTAATTTTTTAAA

Indica_9311 TTCTAGACATCTTATGTTTTTATCCCCACAATAGATTTGTTTTTATTGTCGTGACAAGTTACATTTTTTTAAGTTACTTCTATAATATATATAAATTACTTTTAGGCTTTATTGAATTTAGTTTTTATGTCTAAGAAGTAATTTAATAAAATTTAAAAGTAATTTAAATATATTATAAAAGTAACTTGTAATTTTTTAAA

**Indica_H2_HV** TTCTAGACATCTTATGTTTTTATCCCCACAATAGATTTGTTTTTATTGTCGTGACAAGTTACATTTTTTTAATTTACTTCTATAATATATATAAATTACTTTTAGGCTTTATTGAATTTAGTTTTTATGTCTAAGAAGTAATTTAATGAAATTTAAAAGTAATTTAAATATATTATAAAAGTAACTTGTAATTTTTTTAA

Japonica_NIP TTCTAGACATCTTATGTTTTTATCCCCACAATAGATTTGTTTTTATTGTCGTGACAAGTTACATTTTTTTAATTTACTTCTATAATATATATAAATTACTTTTAGGCTTTATTGAATTTAGTTTTTATGTCTAAGAAGTAATTTAATGAAATTTAAAAGTAATTTAAATATATTATAAAAGTAACTTGTAATTTTTTTAA

**MITE-mariner-like**

Japonica_Kita TTCTAGACATCTTATGTTTTTATCCCCACAATAGATTTGTTTTTATTGTCGTGACAAGTTACATTTTTTTAATTTACTTCTATAATATATATAAATTACTTTTAGGCTTTATTGAATTTAGTTTTTATGTCTAAGAAGTAATTTAATGAAATTTAAAAGTAATTTAAATATATTATAAAAGTAACTTGTAATTTTTTTAA

Aus_N22 TTCTAGACATCTTATGTTTTTATCCCCACAATAGATTTGTTTTTATTGTCGTGACAAGTTACATTTTTTTAAGTTACTTCTATAATATATATAAATTACTTTTAGGCTTTATTGAATTTAGTTTTTATCTCTAAGAAGTAATTTAATAAAATTTAAAAGTAATTTAAATATATTATAAAAGTAACTTGTAATTTTTT-AA

*O. rufipogon* TTCTAGACATCTTATGTTTTTATCCCCACAATAGATTTGTTTTTATTGTCGTGACAAGTTACATTTTTCTAAGTTACTTCTATAATATATATAAATTACTTTTAGGCTTTATTGAATTTAGTTTTTATGTCTAAGAAGTAATTTAATGAAATTTAAAAGTAATTTAAATATATTATAAAAGTAACTTGTAATTTTTTTAA

************************************************************************ ******************************************************* ****************** ************************************************* **

Indica_R498 AAAATATATAATTATATGAGATCAATAAAGATTTAATTGTTACGAACACAATAGTGTAATCGGATCGTAAATCGGATTAGTAGTTTAAGAGAAAATTTTATTTGAAAAATAGGTGATATGAATATATTTCTCTGCTTGCTTCATGGGCTAGTATCCAACAA----ATATTGAGGGGTGAGAGTACTCTTTATTTAGATTT

**Indica_H1_Lv** AAAATAAATAATTATATGAGATCAATAAAGATTTAATTGTTACGAACACAATAGTGTAATCGGATCGTAAATCGGATTAGTAGTTTAAGAGAAAATTTTATTTGAAAAATAGGTGATATGAATATATTTCTCTGCTTGCTTCATGGGCTAGTATCCAACAA----ATATTGAGGGGTGAGAGTACTCTTCATTTAGATTT

Indica_9311 AAAATATATAATTATATGAGATCAATAAAGATTTAATTGTTACGAACACAATAGTGTAATCGGATCGTAAATCGGATTAGTAGTTTAAGAGAAAATTTTATTTGAAAAATAGGTGATATGAATATATTTCTCTGCTTGCTTCATGGGCTAGTATCCAACAA----ATATTGAGGGGTGAGAGTACTCTTTATTTAGATTT

**Indica_H2_HV** AAAATAAATAATTATATGAGATCAATAAAGATTTAATTGTTACGAACACAATAGTGTAATTGGATCGTAAATCGGATTAGTAGTTTAAGAGAAAATTTTATTTGAAAAATAGGTGATATGAATATATTTCTCTGCTTGCTTCATGGGCTAGTATCCAACAA----ATATTGAGGGGTGAGAGTACTCTTCATTTAGATTT

Japonica_NIP AAAATAAATAATTATATGAGATCAATAAAGATTTAATTGTTACGAACACAATAGTGTAATCGGATCGTAAATCGGATTAGTAGTTTAAGAGAAAATTTTATTTGAAAAATAGGTGATATGAATATATTTCTCTGCTTGCTTCATGGGCTAGTATCCAACAA----ATATTGAGGGGTGAGAGTACTCTTCATTTAGATTT

Japonica_Kita AAAATAAATAATTATATGAGATCAATAAAGATTTAATTGTTACGAACACAATAGTGTAATCGGATCGTAAATCGGATTAGTAGTTTAAGAGAAAATTTTATTTGAAAAATAGGTGATATGAATATATTTCTCTGCTTGCTTCATGGGCTAGTATCCAACAA----ATATTGAGGGGTGAGAGTACTCTTCATTTAGATTT

Aus_N22 AAAATATATAATTATATGAGATCAATAAAGATTTAATTGTTACGAACACAATAGTGTAATCGGATCGTAAATCGGATTAGTAGTTTAAGAGAAAATTTTATTTGAAAAATAGGTGATATGAATATATTTCTCTGCTTGCTTCATGGGCTAGTATCCAACAATATAATATTGAGGGGTGAGAGTACTCTTTATTTAGATTT

*O. rufipogon* AAAATAAATAATTATATGAGATCAATAAAGATTTAATTGTTACGAACACAATAGTGTAATCGGATCGTAAATCGGATTAGTAGTTTAAGAGAAAATTTTATTTGAAAAATAGGTGATATGAATATATTTCTCTGCTTGCTTCATGGGCTAGTATCCAACAA----ATATTGAGGGGTGAGAGTACTCTTTATTTAGATTT

****** ***************************************************** **************************************************************************************************** ************************ **********

**WRKY_HD_NAM**

**Indica_H1_Lv** TGCCTTAAAAAAGAAATGCTTTATGTTATCCTGTTTGGACGAGACAATACCTAAGGGCAAGTACTATGATGCTTGATGTATTGCCCCTAAAAATACCACGTAGGATTAGATGATGAGGTGGAAGTAATAAGTAAGGAAAGAGAAGGTGAG------------------------------------CCAAGAAAAATGTG

Indica_R498 TGCCTTAAAAAAGAAATGCTTTATGTTATCCTGTTTGGACGAGACAATACCTAAGGGCAAGTACTATGATGCTTGATGTATTGCCCCTAAAAATACCACGTAGGATTAGATGATGAGGTGGAAGTAATAAGTAAGGAAAGAGAAGGTGAG------------------------------------CCAAGAAAAAGGTG

Indica_9311 TGCCTTAAAAAAGAAATGCTTTATGTTATCCTGTTTGGACGAGACAATACCTAAGGGCAAGTACTATGATGCTTGATGTATTGCCCCTAAAAATACCACGTAGGATTAGATGATGAGGTGGAAGTAATAAGTAAGGAAAGAGAAGGTGAG------------------------------------CCAAGAAAAAGGTG

**Indica_H2_HV** TGCCTTAAAAAAGAAATGCTTTACGTTATCCTGTTTGGACGAGACAATACCTAAGGGCAAGTACTATGATGCTTGATGTATTGCCCCTAAAAATGCCACGTAGGATTGGATGATGAGATGGAAGTAATAAGTGAGGAAAGAGAAGGTGAGTTACCTCTCATGTAAGGGTCAACCTTTACACAAACCCCAAGAAAAATGTG

Japonica_NIP TGCCTTAAAAAAGAAATGCTTTACGTTATCCTGTTTGGACGAGACAATACCTAAGGGCAAGTACTATGATGCTTGATGTATTGCCCCTAAAAATGCCACGTAGGATTGGATGATGAGGTGGAAGTAATAAGTGAGGAAAGAGAAGGTGAGTTACCTCTCATGTAAGGGTCAACCTTTACACAAACCCCAAGAAAAATGTG

**MITE-harbinger**

Japonica_Kita TGCCTTAAAAAAGAAATGCTTTACGTTATCCTGTTTGGACGAGACAATACCTAAGGGCAAGTACTATGATGCTTGATGTATTGCCCCTAAAAATGCCACGTAGGATTGGATGATGAGGTGGAAGTAATAAGTGAGGAAAGAGAAGGTGAGTTACCTCTCATGTAAGGGTCAACCTTTACACAAACCCCAAGAAAAATGTG

Aus_N22 TGCCT------------GCTTTACGTTATCTTGTTTGGACGAGACAATACCTAAGGGCAAGTGCTATGATGCTTGATGTATTGCCCCTAAAAATATCACGTAGGATTAGATGATGAGGTGGAAGTAATAAGTGAGGAAAGAGAAGGTGAGTTACCTTTCATGTAAGGGTCAACCTTTACATAAACCCCAAGAAAAAGGTG

*O. rufipogon* TGCCTTAAAAAAGAAATGCTTTACGTTATCCTGTTTGGACGAGACAATACCTAAGGGCAAGTACTATGATGCTTGATGTATTACCCCTAAAAATGCCACGTAGGATTGGATGATGAGGTGGAAGTAATAAGTGAGAAAAGAGAAGGTGAGTTACCTCTCATGTAAGGGTCAACCTTTACACAAACCAAAAAAAAAAGGTG

***** ****** ****** ******************************* ******************************* *********** ********* ************** ***************** ********** ***

**Indica_H1_Lv** ATGAAAGAAAGAGGGGATTAGATAAACAAATAAAATATAATTATTTGGGTTGGTATGATAGAGGTGGTGTAGATATGGTGTGTTGTATATATTATCTCTTATCTTACATGGATAAAATTAGATGTAGCAACTACCC-TACTATAGAACTTGCCCTAATAGGTATCCCTAATACCTAATTTATGTGTGTCAACAAATGTGT

Indica_R498 ATGAAAGAAAGAGGGGATTGGATAAACAAATAAAATATAATTATTTGGGTTGGTATGATAGAGGTGGTGTAGATATGGTGTGTTGTATATATTATCTCTTATCTTACATGGATAAAATTAGATGTAGCAACTACCC-TACTATAGAACTTGCCCTAATAGGTATCCCTAATACCTAATTTATGTGTGTCAACAAATGTGT

Indica_9311 ATGAAAGAAAGAGGGGATTGGATAAACAAATAAAATATAATTATTTGGGTTGGTATGATAGAGGTGGTGTAGATATGGTGTGTTGTATATATTATCTCTTATCTTACATGGATAAAATTAGATGTAGCAACTACCC-TACTATAGAACTTGCCCTAATAGGTATCCCTAATACCTAATTTATGTGTGTCAACAAATGTGT

**Indica_H2_HV** ATGAAAGAAAGAGGGGATTAGATAAACAAATAAAATATAATTATTTGGGTTAGTATGATAGAGGTGGTGTAGATATGGTGTGTTGTATATATTATCTCTTATCTTACATGGATAAAATTAGATGTAGCAACTACCCCTACTATAGAAGCTGCCCTAATAGGTATCCCTAATACCTAATTTATGTGTGTCAACAAATGTGT

Japonica_NIP ATGAAAGAAAGAGGGGATTAGATAAACAAATAAAATATAATTATTTGGGTTGGTATGATAGAGGTGGTGTAGATATGGTGTGTTGTATATATTATCTCTTATCTTACATGGATAAAATTAGATGTAGCAACTACCCCTACTATAGAACTTGCCCTAATAGGTATCCCTAATACCTAATTTATGTGTGTCAACAAATGTGT

Japonica_Kita ATGAAAGAAAGAGGGGATTAGATAAACAAATAAAATATAATTATTTGGGTTGGTATGATAGAGGTGGTGTAGATATGGTGTGTTGTATATATTATCTCTTATCTTACATGGATAAAATTAGATGTAGCAACTACCCCTACTATAGAACTTGCCCTAATAGGTATCCCTAATACCTAATTTATGTGTGTCAACAAATGTGT

Aus_N22 ATGAAAGAAAGAGGGGATTGGATAAACAAATAAAATATAATTATTTGGGTTGGTATGATAGAGGTGGTGTAGATATGGTGTGTTGTATATATTATCTCTTATCTTACATGGATAAAATTAGATATAGCAACTACCCCTACTATAGAACTTGCCCTAATAGGTATCCCTAATACCTAATTTATGTGTGTCAACAAATGTGT

*O. rufipogon* ATGAAAGAAAGAGGGGATTGGATAAATAAATAAAATATAATTATTTGGGTTGGTATGATAGAGGTGGTGTAGATATGGTGTGTTGTATATATTATCTCTTATCTTATATGGATAAAATTAGATGTAGCAACTACCCCTACTATAGAACTTGCCCTAATAGGTATCCCTAGTACCTAATTTATGCGTGTCAACAAATGTGT

******************* ******************************* *********************************************************************** ************ ********** ***************************************************

**Indica_H1_Lv** ACCCGC**A**

Indica_R498 ACCCGC**A**

Indica_9311 ACCCGC**A**

**Indica_H2_HV** ACCCGC**A**

Japonica_NIP ACCCGC**A**

Japonica_Kita ACCCGC**A**

Aus_N22 ACCCGC**A**

*O. rufipogon* ACCCGC**A**

***********

***Loc_Os02g29210* promoter**

**Indica_H1_Lv** ATTTCGATCACTTTTAAGGGTCATTAAGTGAACTTATTCCCAAGCCTTTTAGTTTGGGTTTCTTTTGGATGGGTAAGCTTTTCTTTTTCCTTCCTAAGCAAAAGGTCCATGTGTTGCAATGGATATTTTTATTGGCTCGGTTTCTATTATAGTAAATTTCTCATTATGCGTATAATACTTAAAAAAA-TATGCTCATGCG

Indica_R498 ATTTCGATCACTTTTGAGGGTCATTAAGTGAACTTATTCCCAAGCCTTTTAGTTTGGGTTTCTTTTGGATGGGTAAGCTTTTCTTTTTCCTTCCTAAGCAAAAGGTCCATGTGTTGCAATGGATATTTTTATTGGCTCGGTTTCTATTATAGTAAATTTCTCATTATGCGTATAATACTTAAAAAAA-TATGCTCATGCG

Indica_9311 ATTTCGATCACTTTTGAGGGTCATTAAGTGAACTTATTCCCAAGCCTTTTAGTTTGGGTTTCTTTTGGATGGGTAAGCTTTTCTTTTTCCTTCCTAAGCAAAAGGTCCATGTGTTGCAATGGATATTTTTATTGGCTCGGTTTCTATTATAGTAAATTTCTCATTATGCGTATAATACTTAAAAAAA-TATGCTCATGCG

**Indica_H2_HV** ATTTCAATCACTTTTAAGGGTCATTAAGTGAACTTATTCCCAAGCCTTTTAGTTTGGGTTTCTTTTGGATGGGTAAGCTTTTCTTTTTCCTTACTAAGCAAAAGGTCCATGTGTTGCAATGGATATTTTTATTGGCTCGGTTTCTATTATAGTAAATTTCTCATTATGCGTATAATACTTAAAAAAAATATGCTCATGCG

Japonica_NIP ATTTCGATCACTTTTAAGGGTCATTAAGTGAACTTATTCCCAAGCCTTTTAGTTTGGGTTTCTTTTGGATGGGTAAGCTTTTCTTTTTCCTTACTAAGCAAAAGGTCCATGTGTTGCAATGGATATTTTTATTGGCTCGGTTTCTATTATAGTAAATTTCTCATTATGCGTATAATACTTAAAAAAA-TATGCTCATGCG

Japonica_Kita ATTTCGATCACTTTTAAGGGTCATTAAGTGAACTTATTCCCAAGCCTTTTAGTTTGGGTTTCTTTTGGATGGGTAAGCTTTTCTTTTTCCTTACTAAGCAAAAGGTCCATGTGTTGCAATGGATATTTTTATTGGCTCGGTTTCTATTATAGTAAATTTCTCATTATGCGTATAATACTTAAAAAAA-TATGCTCATGCG

Aus_N22 ATTTCGATCACTTTTGAGGGTCATTAAGTGAACTTATTCCCAAGCCTTTTAGTTTGGGTTTCTTTTGGATGGGTAAGCTTTTCTTTTTCCTTACTAAGCAAAAGGTCCATGTGTTGCAATGGATATTTTTATTGGCTCGGTTTCTATTATAGTAAATTTCTCATTATGCGTATAATACTTAAAAAAA-TATGCTCATGCG

*O. rufipogon* ATTTCGATCACTTTTGAGGGTCATTAAGTGAACTTATTCCCAAGCCTTTTAGTTTGGGTTTCTTTTGGATGGGTAAGCTTTTCTTTTTCCTTACTAAGCAAAAGGTCCATGTGTTGCAATGGATATTTTTATTGGCTCGGTTTCTATTATAGTAAATTTCTCATTATGCGTATAATACTTAAAAAAA-TATGCTCATGCG

***** ********* **************************************************************************** ********************************************************************************************** ************

**Indica_H1_Lv** GTTATCCCTGGCGTAAGAGTGAATTAGACCTTATTTTAAATTATGCATTTAATACTTTAAAAGAAGGATACTCATGCGGTTGTCCCTCGTGTTTGTTCTGCTTTTGTAATATCGCTGATAAC--GGACCCATATTAAGAGGATAAATAAGGATTGAGAAGAGCAGCGGGCTATAAATTTGTAGCCAGCTGTAGCACGAAC

Indica_R498 GTTATCCCTGGCGTAAGAGTGAATTAGACCTTATTTTAAATTATGCATTTAATACTTTAAAAGAAGGATACTCATGCGGTTGTCCCTCGTGTTTGTTCTGCTTTTGTAATATCGCTGATAAC--GGACCCATATTAAGAGGATAAATAAGGATTGAGAAGAGCAGCGGGCTATAAATTTGTAGCCAGCTGTAGCACGAAC

Indica_9311 GTTATCCCTGGCGTAAGAGTGAATTAGACCTTATTTTAAATTATGCATTTAATACTTTAAAAGAAGGATACTCATGCGGTTGTCCCTCGTGTTTGTTCTGCTTTTGTAATATCGCTGATAAC--GGACCCATATTAAGAGGATAAATAAGGATTGAGAAGAGCAGCGGGCTATAAATTTGTAGCCAGCTGTAGCACGAAC

**Indica_H2_HV** GTTATCCCTGGCGTAAGAGTGAATTAGACCTTATTTTAAATTATGCATTTAATACTTTAAAAGAAGGATACTCATGCGGTTGTCCCTCGTGTTTGTTCTGCTTTTGTAATATCGCTGATAACAGGGACCCATATTAAGAGGATAAATAAGGAGAGAGAAGAGCAGCGGGCTATAAATTTATAGCCAGCTGTAGCACGAAC

Japonica_NIP GTTATCCCTGGCGTAAGAGTGAATTAGACCTTATTTTAAATTATGCATTTAATACTTTAAAAGAAGGATACTCATGCGGTTGTCCCTCGTGTTTGTTCTGCTTTTGTAATATCGCTGATAACA-GGACCCATATTAAGAGGATAAATAAGGAGAGAGAAGAGCAGCGGGCTATAAATTTGTAGCCAGCTGTAGCACGAAC

Japonica_Kita GTTATCCCTGGCGTAAGAGTGAATTAGACCTTATTTTAAATTATGCATTTAATACTTTAAAAGAAGGATACTCATGCGGTTGTCCCTCGTGTTTGTTCTGCTTTTGTAATATCGCTGATAACA-GGACCCATATTAAGAGGATAAATAAGGAGAGAGAAGAGCAGCGGGCTATAAATTTGTAGCCAGCTGTAGCACGAAC

Aus_N22 GTTATCCCTGGCGTAAGAGTGAATTAGACCTTATTTTAAATTATGCATTTAATATTTTAAAAGAAGGATACTCATGCGGTTGTCCCTCGTGTTTGTTCTGCTTTTGTAATATCGCTGATAAC-GGGACCCATATTAAGAGGATAAATAAGGAGAGAGAAGAGCATCGGGCTATAAATTTGTAGCCAGCTGTAGCACGAAC

*O. rufipogon* GTTATCCCTGGCGTAAGAGTGAATTAGACCTTATTTTAAATTATGCATTTAATACTTTAAAAGAAGGATACTCATGCGGTTGTCCCTCGTGTTTGTTCTGCTTTTGTAATATCGCTGATAAC-GGGACCCATATTAAGAGGATAAATAAGGAGAGAGAAGAGCAGCGGCCTATAAATTTATAGTCAGCTGTAGCACGAAC

**B3**

**AT-hook**

**MITE-harbinger-like**

****************************************************** ******************************************************************* **************************** ********** ************** ********************

**Indica_H1_Lv** TCCAAGACACAGTGTGTATGACAGGTGGGGCCAGATATTAATAGTGTAGTATGTAACTATTATATGAATGAGTTATTAGATTGACTATAAATAAATTAAAACTAGTAGTTGGCTATACTATTAAACTTGCTCAAACTGTCGGTGAAGGGACTGAAAAATCTAACTTGGTTCGAGGTTAGATTGATCCCTGATTCCCCACC

Indica_R498 TCCAAGACACAGTGTGTATGACAGGTGGGGCCAGATATTAATAGTGTAGTATGTAACTATTATATGAATGAGTTATTAGATTGACTATAAATGAATTAGAGCTAGTAGTTGGCTATACTATTAAACTTGCTCAAACTGTCGGTGAAGGGACTGAAAAATCTAACTTGGTTCGAGGTTAGATTGATCCCTGATTCCCCACC

Indica_9311 TCCAAGACACAGTGTGTATGACAGGTGGGGCCAGATATTAATAGTGTAGTATGTAACTATTATATGAATGAGTTATTAGATTGACTATAAATGAATTAGAGCTAGTAGTTGGCTATACTATTAAACTTGCTCAAACTGTCGGTGAAGGGACTGAAAAATCTAACTTGGTTCGAGGTTAGATTGATCCCTGATTCCCCACC

**Indica_H2_HV** TCCAAGACACAGTGTGTATGACAGGTGGGGCCAGGTATTAATAGTGTAGTATGTAA------------TAAGCTATTAGATTGACTATAGATAAATTGAAACTACTAGTTGGCTATACTATTAAACTTGCTCTAACTGTCGGTGAAGGGACTGAAAAATCTAACTTGGTTCGAGGTTAGATTGATCCCCGATTCCCCACC

Japonica_NIP TCCAAGACACAGTGTGTATGACAGGTGGGGCCAGGTATTAATAGTGTAGTATGTAACTATTATATGAATGAGCTATTAGATTGACTATAGATAAATTGAAACTAGTAGTTGGCTATACTATTAAACTTGCTCTAACTGTCGGTGAAGGGACTGAAAAATCTAACTTGGTTCGAGGTTAGATTGATCCCCGATTCCCCACC

Japonica_Kita TCCAAGACACAGTGTGTATGACAGGTGGGGCCAGGTATTAATAGTGTAGTATGTAACTATTATATGAATGAGCTATTAGATTGACTATAGATAAATTGAAACTAGTAGTTGGCTATACTATTAAACTTGCTCTAACTGTCGGTGAAGGGACTGAAAAATCTAACTTGGTTCGAGGTTAGATTGATCCCCGATTCCCCACC

Aus_N22 TTCAAGACACAGTGTGTATGACAGATGGGGCCAGGTATTAATAGTGTAGTATGTAACTATTATATGAATGAGCTATTAGATTGACTATAGATGAATTAGAGCTAGTAGTTGGCTATACTATTAAACTTGCTCTAACTGTCGGTGAAGGGACTGAAAAATCTAACTTGGTTCGAGGTTAGATTGATCCCCGATTCCCCACC

*O. rufipogon* TCCAAGACACAGTGTGTATGACAGGTGGGGCCAGGTATTAATAGTGTAGTATGTAACTATTATATGAATGAGCTATTAGATTGACTATAGATGAATTGAAGCTAGTAGTTGGCTATACTATTAAACTTGCTCTAACTGTCGGTGAAGGGACTGAAAAATCTAACTTGGTTCGAGGTTAGATTGATCCCCGATTCCCCACC

* ********************** ********* ********************* * ** **************** ** **** * *** *************************** ******************************************************* ***********

**Indica_H1_Lv** CCGACTTATGTCGTCTTCTCGTCTCCCTTCATCTCTCTCCCTCTCGCCGCTTCCTCCGCGACAGCAAGCCGTCACCGCCACTCCAAGCTGCGTCCCTCCCTCGTCGTCGCACTGTTCTAGTGGAGTTCTAGTCGGCGGTGTGCCTAAGTCCAAGCCTGGTGCCCTAGCAAGCTGAGGCTGGCGACGCCAAGGTGATGGCA

Indica_R498 CCGACTTATGTCGTCTTCTCGTCTCCCTTCATCTCTCTCCCTCTCGCCGCTTCCTCCGCGACAGCAAGCCGTCACCGCCACTCCAAGCTGCGTCCCTCCCTCGTCGTCGCACTGTTCTAGTGGAGTTCTAGTCGGCGGTGTGCCTAAGTCCAAGCCTGGTGCCCTAGCAAGCTGAGGCTGGCGACGCCAAGGTGATGGCA

Indica_9311 CCGACTTATGTCGTCTTCTCGTCTCCCTTCATCTCTCTCCCTCTCGCCGCTTCCTCCGCGACAGCAAGCCGTCACCGCCACTCCAAGCTGCGTCCCTCCCTCGTCGTCGCACTGTTCTAGTGGAGTTCTAGTCGGCGGTGTGCCTAAGTCCAAGCCTGGTGCCCTAGCAAGCTGAGGCTGGCGACGCCAAGGTGATGGCA

**Indica_H2_HV** CCGACTTATCTCGTCTTCTCGTCTCCCTTCATCTCTCTCCCTCTCGCCGATTCCTCCGCGACAGCAAGCCGTCACCGCCACTCCAAGCTGCGTCCCTCCCTCGTCGTCGCACGGTTCTAGTGGAGTTCTAGTCGGCGGTGTGCCTAAGTCCAAGCCTGGTGCCCTAGCAAGCTGAGGCTGGCGACGCCAAGGTGATGGCA

Japonica_NIP CCGACTTATCTCGTCTTCTCGTCTCCCTTCATCTCTCTCCCTCTCGCCGCTTCCTCCGCGACAGCAAGCCGTCACCGCCACTCCAAGCTGCGTCCCTCCCTCGTCGTCGCACGGTTCTAGTGGAGTTCTAGTCGGCGGTGTGCCTAAGTCCAAGCCTGGTGCCCTAGCAAGCTGAGGCTGGCGACGCCAAGGTGATGGCA

Japonica_Kita CCGACTTATCTCGTCTTCTCGTCTCCCTTCATCTCTCTCCCTCTCGCCGCTTCCTCCACGACAGCAAGCCGTCACCGCCACTCCAAGCTGCGTCCCTCCCTCGTCGTCGCACGGTTCTAGTGGAGTTCTAGTCGGCGGTGTGCCTAAGTCCAAGCCTGGTGCCCTAGCAAGCTGAGGCTGGCGACGCCAAGGTGATGGCA

Aus_N22 CCGACTTATCTCGTCTTCTCGTCTCCCTTCATCTCTCTCCCTCTCGCCGCTTCCTCCGCGACAGCAAACCGTCACCGCCACTCCAAGCTGCGTCCCTCCCTCGTCGTCGCACGGTTCTAGTGGAGTTCTAGTCGGCGGTGTGCCTAAGTCCAAGCCTGGTGCCCTAGCAAGCTGAGGCTGGCGACGCCAAGGTGATGGCA

*O. rufipogon* CCGACTTATCTCGTCTTCTCGTCTCCCTTCATCTCTCTCCCTCTCGCCGCTTCCTCCGCGACAGCAAGCCGTCACCGCCACTCCAAGCTGCGTCCCTCCCTCGTCGTCGCACGGTTCTAGTGGAGTTCTAGTCGGCGGTGTGCCTAAGTCCAAGCCTGGTGCCCTAGCAAGCTGAGGCTGGCGACGCCAAGGTGATGGCA

********* *************************************** ******* ********* ******************************************** ***************************************************************************************

**Indica_H1_Lv** GCGGCGGCGGTTATGGCGTGCGGTGAGCTTAGGCCGGCATGGCGCTCCCTGCCGGTTCTTCTCCTCTGTGCAGCAGAAGCCACGGTGGTGGCGAGCCTCGCTCTTGTGCAGCCAACAAAGCCAGGGGCGAGCCCCGTCCGGGAGGTAGAGATGAGCTAATACGGGACTGATGCTATGCGCGGTAGGCTCTGATGATCTGA

Indica_R498 GCGGCGGCGGTTATGGCGTGCGGTGAGCTTAGGCCGGCATGGCGCTCCCCGCCGGTTCTTCTCCTCTGTGCAGCAGAAGCCACGGTGGTGGCGAGCCTCGCTCTTGTGCAGCCAACAAAGCCAGGGGCGAGCCCCGTCCGGGAGGTGGAGATGAGCTAATACGGGACTGATGCTATGCGCGGTAGGCTCTGACGATCTGA

Indica_9311 GCGGCGGCGGTTATGGCGTGCGGTGAGCTTAGGCCGGCATGGCGCTCCCCGCCGGTTCTTCTCCTCTGTGCAGCAGAAGCCACGGTGGTGGCGAGCCTCGCTCTTGTGCAGCCAACAAAGCCAGGGGCGAGCCCCGTCCGGGAGGTGGAGATGAGCTAATACGGGACTGATGCTATGCGCGGTAGGCTCTGACGATCTGA

**Indica_H2_HV** GCGGCGGCGGTTATGGCGTGCGGTGAGCTTAGGCCGGCATGGCGCTCCCTGCCGGTTCTTCTCCTCTGTGCAGCAGAAGCCACGGTGGTGGCAAGCCTTGCTCTTGTGCAGCCAACAAAGCCAGGGGCGAGCCCCGTCCGGGAGGTAGAGATGAGCTAATACGGGACTGATGCTATGCGCGGCAGGCTCTGATGATCTGA

Japonica_NIP GCGGCGGCGGTTATGGCGTGCGGTGAGCTTAGGCCGGCATGGCGCTCCCTGCCGGTTCTTCTCCTCTGTGCAGCAGAAGCCACGGTGGTGGCGAGCCTCGCTCTTGTGCAGCCAACAAAGCCAGGGGCGAGCCCCGTCCGGGAGGTAGAGATGAGCTAATACGGGACTGATGCTATGCGCGGCAGGCTCTGATGATCTGA

Japonica_Kita GCGGCGGCGGTTATGGCGTGCGGTGAGCTTAGGCCGGCATGGCGCTCCCTGCCGGTTCTTCTCCTCTGTGCAGCAGAAGCCACGGTGGTGGCGAGCCTCGCTCTTGTGCAGCCAACAAAGCCAGGGGCGAGCCCCGTCCGGGAGGTAGAGATGAGCTAATACGGGACTGATGCTATGCGCGGCAGGCTCTGATGATCTGA

Aus_N22 GCGGCGGCGGTTATGGCGTGTGGTGAGCTTAGGCCGGCATGGCGCTCCCCGCCGGTTCTTCTCCTCTGTGCAGCAGAAGCCACGGTGGTGGCGAGCCTCGCTCTTGTGCAGCCAACAAAGCCAGGGGCGAGCCCCGTCCGGGAGGTGGAGATGAGCTAATACGGGACTGATGCTATGCGCGGCAGGCTCTGACGATCTGA

*O. rufipogon* GCGGCGGCGGTTATGGCATGCGGTGAGCTTAGGCCGGCATGGCGCTCCCCGCCGGTTCTTCTCCTCTGTGCAGCAGAAGCCACGGTGGTGGCGAGCCTCGCTCTTGTGCAGCCAACAAAGCCAGGGGCGAGCCCCGTCTGGGAGGTGGAGATGAGCTAATACGGGACTGATGCTATGCGCGGTAGGCTCTAACGATCTGA

**NF-YB**

**NF-YB**

**NF-YB**

******************** **************************** ****************************************** ***** *********************************************** *********************************** ********* *******

**HD-zip/WOX**

**Indica_H1_Lv** TGCTGCCAAGACAACGAGTAGGACCCCGATTCGGTAAGTCTAAGCCCCAATTGCTTAGGAATTTGATTGATTTGGTGTTTTTATTCCGGTATCCACTTAATTTTTCTTGGGGGGAGATTCTGAGTCCTTCCAGGAGAGGGTGGAGTCCTTCCACGCGCGCAGGAGGAGTCGGATGCGGATGACGACGAACGAAAATCTTC

Indica_R498 TGCTGCCAAGACAACGAGTAGGACCCCGATTCGGTAAGTCTAAGCCCCAATTGCTTAGGAATTTGATTGATTTGGGGTTTTTATTCCGGTATCCACTTAATTTTTCTTGGGGGGAGATTCTGAGTCCTTCCAGGAGAGGGTGGAGTCCTTCCACGCGCGCAGGAGGAGTCGGATGCGGATGACGACGAACGAAAATCTTC

Indica_9311 TGCTGCCAAGACAACGAGTAGGACCCCGATTCGGTAAGTCTAAGCCCCAATTGCTTAGGAATTTGATTGATTTGGGGTTTTTATTCCGGTATCCACTTAATTTTTCTTGGGGGGAGATTCTGAGTCCTTCCAGGAGAGGGTGGAGTCCTTCCACGCGCGCAGGAGGAGTCGGATGCGGATGACGACGAACGAAAATCTTC

**Indica_H2_HV** TGCTGCCAAGACAACAAGTAGGACCCC----------------------------------------TGATTTGGTGTTTTTATTCCGATATCCACTTAATTTTTCTTGGGGGGAGATTCTGAGTCCTTCCAGGAGAGGGTGGAGTCCTTCCACGCGCGCAGGAGGAGTCGGATGCGGATGACGACGAACGAAAATCTTC

Japonica_NIP TGCTGCCAAGACAACGAGTAGGACCCCGATTCGGTAAGTCTAAGCCCCAATTGCTTAGGAATTTGATTGATTTGGTGTTTTTATTCCGGTATCCACTTAATTTTTCTTGGGGGGAGATTCTGAGTCCTTCCAGGAGAGGGTGGAGTCCTTCCACGCGCGCAGGAGGAGTCGGATGCGGATGACGACGAACGAAAATCTTC

Japonica_Kita TGCTGCCAAGACAACGAGTAGGACCCCGATTCGGTAAGTCTAAGCCCCAATTGCTTAGGAATTTGATTGATTTGGTGTTTTTATTCCGGTATCCACTTAATTTTTCTTGGGGGGAGATTCTGAGTCCTTCCAGGAGAGGGTGGAGTCCTTCCACGCGCGCAGGAGGAGTCGGATGCGGATGACGACGAACGAAAATCTTC

Aus_N22 TGCTGCCAAGACAACGAGTAGGACCCTGATTCGGTAAGTCTAAGCCCCAATTGCTTAGGAATTTGATTGATTTGGGGTTTTTATTCCGGTATCCACTTAATTTTTCTTGGGGGGAGATTCTGAGTCCTTCCAGGAGAGGGTGGAGTCCTTCCACGCGCGCAGGAGGAGTCGGATGCGGATGACGACGAACGAAAATCTTC

*O. rufipogon* TGCTGCCAAGACAACGAGTAGGACCCCGATTCGGTAAGTCTAAGCCCCAATTGCTTAGGAATTTGATTGATTTGGGGTTTTTATTCCGGTATCCACTTAATTTTTCTTGGGGGGAGATTCTGAGTCCTTCCAGGAGAGGGTGGAGTCCTTCCACACGTGCAGGAGGAGTCGGATGCGGATGACGACGAACGAAAATCTTC

*************** ********** ******** ************ ***************************************************************************************************************

**Indica_H1_Lv** CGAAAGCCTTACATGGTGGCCATCGCGAGCGACGGACGGAAAAACCGATGTGACGATGGGAAAAATATCAAATAGTTAAACGTTGTAAGATTTATATATTTAGATTCACCGTGTAAAATACTTTCCTAATACATGATACACATGTTGTTAAATGATATGGGTTTTCAAAGATGTATCGACTTTGGACAAGAACGCCAATT

Indica_R498 CGAAAGCCTTACATGGTGGCCATCGCGAGCGACGGACGGAAAAACCGATGTGACGATGGGAAAAATATCAAATAGTTAAACGTTGTAAGATTTATATATTTAGATTCACCGTGTAAAATACTTTCCTAATACATGATACACATGTTGTTAAATGATATGGGTTTTCAAAGATATATCGACTTTGGACAAGAACGCCAATT

Indica_9311 CGAAAGCCTTACATGGTGGCCATCGCGAGCGACGGACGGAAAAACCGATGTGACGATGGGAAAAATATCAAATAGTTAAACGTTGTAAGATTTATATATTTAGATTCACCGTGTAAAATACTTTCCTAATACATGATACACATGTTGTTAAATGATATGGGTTTTCAAAGATATATCGACTTTGGACAAGAACGCCAATT

**Indica_H2_HV** CGAAAGCCTTACATGGTGGCCATCGCGAGCGACGGACGGAAAAACCGATGTGACGATGGGAAAAATATCAAATAGTTAAACGTTGTAAGATTTATATATTTAGATTCACCGTGTAAAATACTTTCCTAATACATGATACACATGTTGTTAAATGATATGGGTTTTCAAAGATGTATCGACTTTGGACAAGAACGACAATT

Japonica_NIP CGAAAGCCTTACATGGTGGCCATCGCGAGCGACGGACGGAAAAACCGATGTGACGATGGGAAAAATATCAAATAGTTAAACGTTGTAAGATTTATATATTTAGATTCACCGTGTAAAATACTTTCCTAATACATGATACACATGTTGTTAAATGATATGGGTTTTCAAAGATGTATCGACTTTGGACAAGAACGACAATT

Japonica_Kita CGAAAGCCTTACATGGTGGCCATCGCGAGCGACGGACGGAAAAACCGATGTGACGATGGGAAAAATATCAAATAGTTAAACGTTGTAAGATTTATATATTTAGATTCACCGTGTAAAATACTTTCCTAATACATGATACACATGTTGTTAAATGATATGGGTTTTCAAAGATGTATCGACTTTGGACAAGAACGACAATT

Aus_N22 CGAAAGCCTTACATGGTGGCCATCGCGAGCGACGGACGGAAAAACCGATGTGACGATGGGAAAAATATCAAATAGTTAAACGTTGTAAGATTTATATATTTAGATTCACCGTGTAAAATACTTTCCTAATACATGATACACATGTTGTTAAATGATATGGGTTTTCAAAGATATATCGACTTTGGACAAGAACGACAATT

*O. rufipogon* CGAAAGCCTTACATGGTGGCCATCGCGAGCGACGGACGGAAAAACCGATGTGACGATGGGAAAAATATCAAATAGTTAAACGTTGTAAGATTTATATATTTAGATTCACCGTGTAAAATACTTTCCTAATACATGATACACATGTTGTTAAATGATATGGGTTTTCAAAGATATATCGACTTTGGACAAGAACGACAATT

**************************************************************************************************************************************************************************** ********************* *****

**Indica_H1_Lv** AATTTAAAGATTATGTCGTTTAGAAATTCGAAAAACATAGTACGAATTAATCCATTCGTCGAAAACAAACAGGTCGGCCCATCGATGAGTCCGATCCATCATCCATGCGTGAGCAACTGAGCATACGAGTACCCGCAGCAATTTCCAGCTAAGCGAGTGAACAGTGAACTGATGACACACTATACTTCTCCCCGTGACT

Indica_R498 AATTTAAAGATTATGTCGTTTAGAAATTCGAAAAACATAGTACGAATTAATCCATTCGTCGAAAACAAACAGGTCGGCCCATCGATGAGTCCGATCCATCATCCATGCGTGAGCAACTGAG------------------------------------------------------------------------------

Indica_9311 AATTTAAAGATTATGTCGTTTAGAAATTCGAAAAACATAGTACGAATTAATCCATTCGTCGAAAACAAACAGGTCGGCCCATCGATGAGTCCGATCCATCATCCATGCGTGAGCAACTGAGCATACGAGGACCCGCAGCAATTTCCAGCTAAGCGAGTGAACAGTGAACTGATGACACACTATACTTCTCCCCGTGACT

**Indica_H2_HV** AATTTAAAGATTATGTCGTTTAGAAATTCGAAAAACATAGTACGAATTAATCCATTCGTCGAAAACAAACAGGTCGGCCCATCGATGAGTCCGATCCATCATCCATGCGTGAGCAACTGAGCATACGAGTACCCGCAGCAATTTCCAGCTAAGCGAGTGAACAGTGAACTGATGACACACTATACTTCTCCCCGTGACT

Japonica_NIP AATTTAAAGATTATGTCGTTTAGAAATTCGAAAAACATAGTACGAATTAATCCATTCGTCGAAAACAAACAGGTCGGCCCATCGATGAGTCCGATCCATCATCCATGCGTGAGCAACTGAGCATACGAGTACCCGCAGCAATTTCCAGCTAAGCGAGTGAACAGTGAACTGATGACACACTATACTTCTCCCCGTGACT

Japonica_Kita AATTTAAAGATTATGTCGTTTAGAAATTCGAAAAACATAGTACGAATTAATCCATTCGTCGAAAACAAACAGGTCGGCCCATCGATGAGTCCGATCCATCATCCAGGCGTGAGCAACTGAGCATACGAGTACCCGCAGCAATTTCCAGCTAAGCGAGTGAACAGTGAACTGATGACACACTATACTTCTCCCCGTGACT

Aus_N22 AATTTAAAGATTATGTCGTTTAGAAATTCGAAAAACATAGTACGAATTAATCCATTCGTCGAAAACAAACAGGTCGGCCCATCGATGAGTCCGATCCATCATCCATGCGTGAGCAACTGAGCATACGAGTACCCGCAGAAATTTCCAGCTAAGCGAGTGAACAGTGAACTGATGACACACTATACTTCTCCCCGTGACT

*O. rufipogon* AATTTAAAGATTATGTCGTTTAGAAATTCGAAAAACATAGTACGAATTAATCCATTCGTCGAAAACAAACAGGTCGGCCCATCGATGAGTCCGATCCATCATCCATGCGTGAGCAACTGAGCATACGAGTACCCGCAGCAATTTCCAGCTAAGCGAGTGAACAGTGAACTGATGACACACTATACTTCTCCCCGTGACT

********************************************************************************************************* ***************
