# Supplementary material for: A cluster of Ankyrin and Ankyrin-TPR repeat genes is associated with panicle branching diversity in rice
Source: PLoS Genet. 2021 Jun 7;17(6):e1009594. doi: 10.1371/journal.pgen.1009594 (PMC8211194; doi:10.1371/journal.pgen.1009594)
Supplement: S13 Fig — Expression profiles of LOC_Os02g29040 (A), LOC_Os02g29160 (B), LOC_Os02g19190 (C) and LOC_Os2g29210 (D) genes and the co-expressed transcription factor-encoding genes in the laser-dissected panicle meristems according to [27]. M1 = rachis meristem; M2 = primary branch meristem; M3 = elongated primary branch meristem with axillary meristems; M4 = spikelet meristem. The list of TF genes was defined according the list of genes associated with the putative TBDSs (with a similar score ≥ 0.8) based on the search in PlantPAN3.0 website facilities. (PDF) [file pgen.1009594.s013.pdf]

A

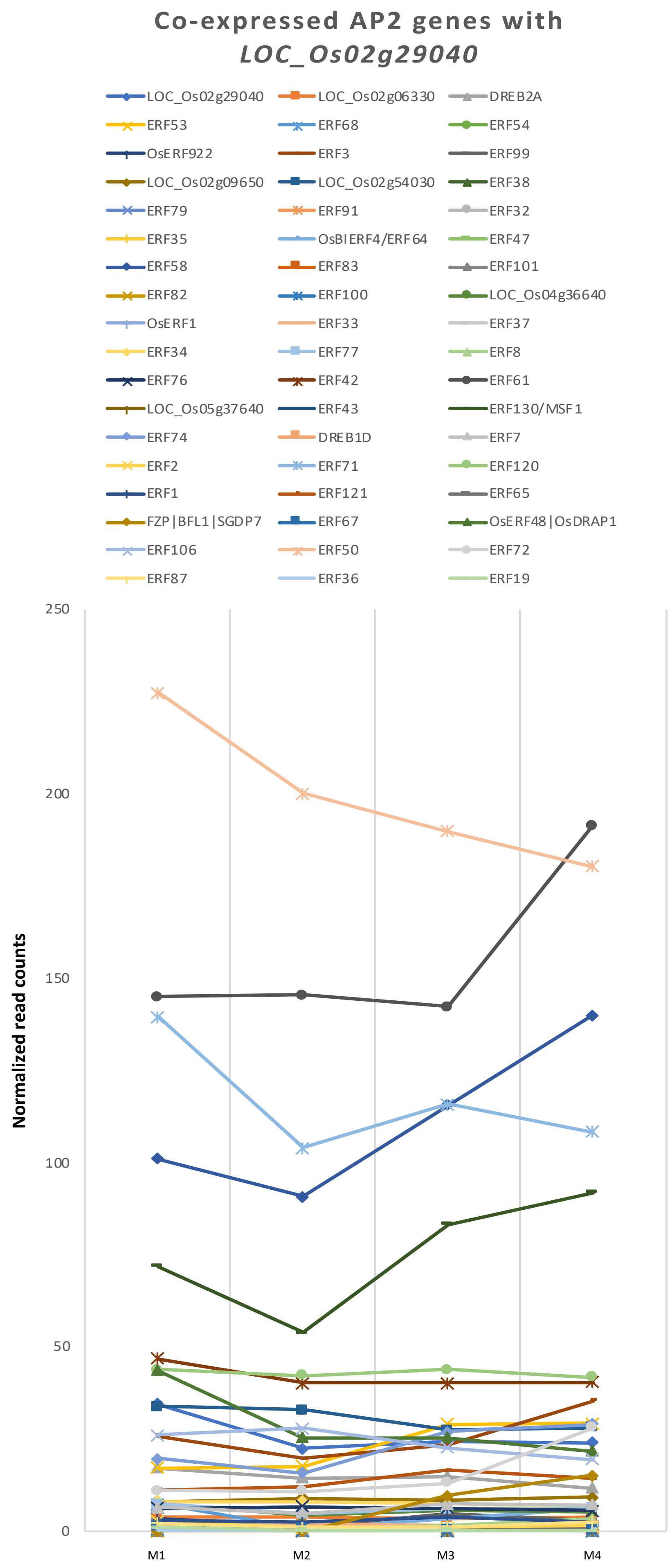
Co-expressed AP2 genes with *LOC_Os02g29040*
LOC_Os02g29040
LOC_Os02g06330
DREB2A
ERF53
ERF68
ERF54
OsERF922
ERF3
ERF99
LOC_Os02g09650
LOC_Os02g54030
ERF38
ERF79
ERF91
ERF32
ERF35
OsBIERF4/ERF64
ERF47
ERF58
ERF83
ERF101
ERF82
ERF100
LOC_Os04g36640
OsERF1
ERF33
ERF37
ERF34
ERF77
ERF8
ERF76
ERF42
ERF61
LOC_Os05g37640
ERF43
ERF130/MSF1
ERF74
DREB1D
ERF7
ERF2
ERF71
ERF120
ERF1
ERF121
ERF65
FZP|BFL1|SGDP7
ERF67
OsERF48|OsDRAP1
ERF106
ERF50
ERF72
ERF87
ERF36
ERF19
Normalized read counts
250
200
150
100
50
0
M1
M2
M3
M4

B

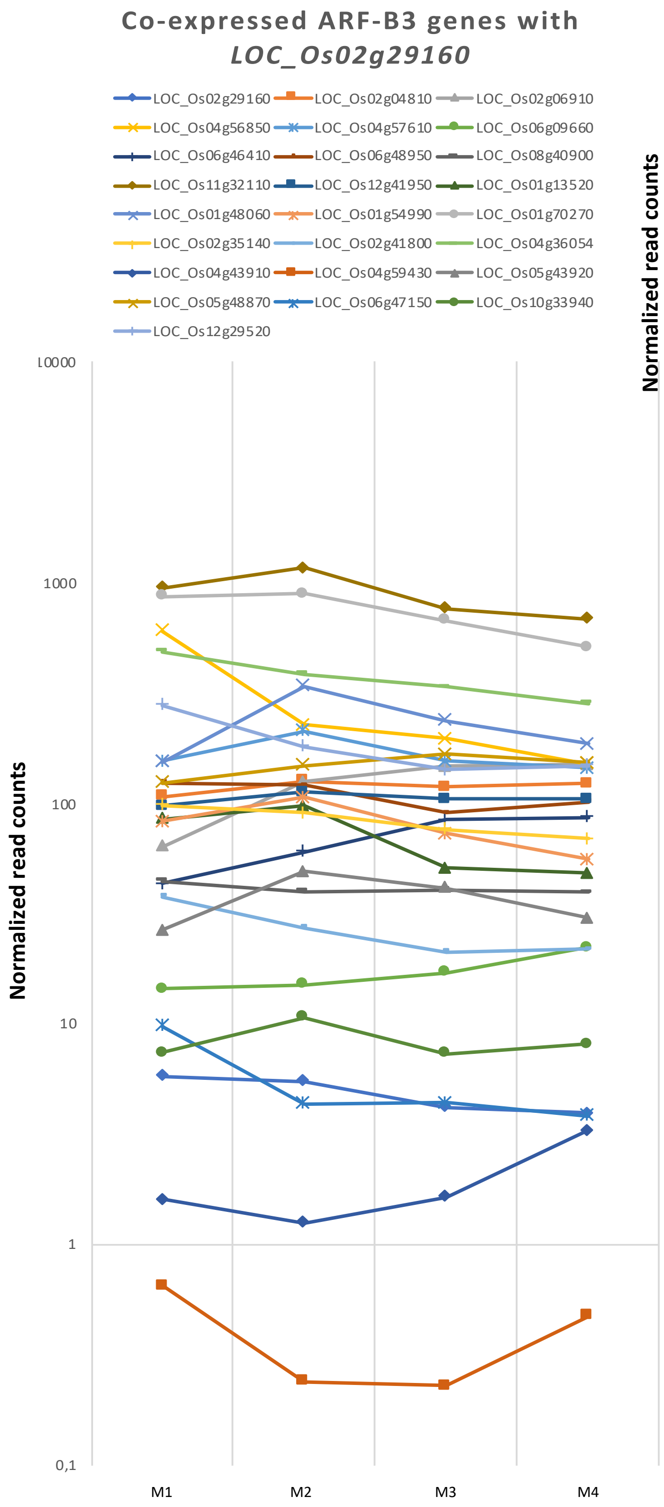
Co-expressed ARF-B3 genes with *LOC_Os02g29160*
LOC_Os02g29160
LOC_Os02g04810
LOC_Os02g06910
LOC_Os04g56850
LOC_Os04g57610
LOC_Os06g09660
LOC_Os06g46410
LOC_Os06g48950
LOC_Os08g40900
LOC_Os11g32110
LOC_Os12g41950
LOC_Os01g13520
LOC_Os01g48060
LOC_Os01g54990
LOC_Os01g70270
LOC_Os02g35140
LOC_Os02g41800
LOC_Os04g36054
LOC_Os04g43910
LOC_Os04g59430
LOC_Os05g43920
LOC_Os05g48870
LOC_Os06g47150
LOC_Os10g33940
LOC_Os12g29520
Normalized read counts
10000
1000
100
10
1
0,1
M1
M2
M3
M4

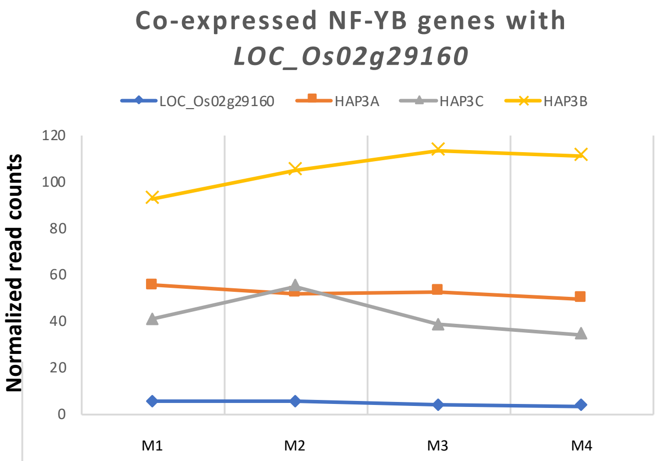
Co-expressed NF-YB genes with *LOC_Os02g29160*
LOC_Os02g29160
HAP3A
HAP3C
HAP3B
Normalized read counts
120
100
80
60
40
20
0
M1
M2
M3
M4

C

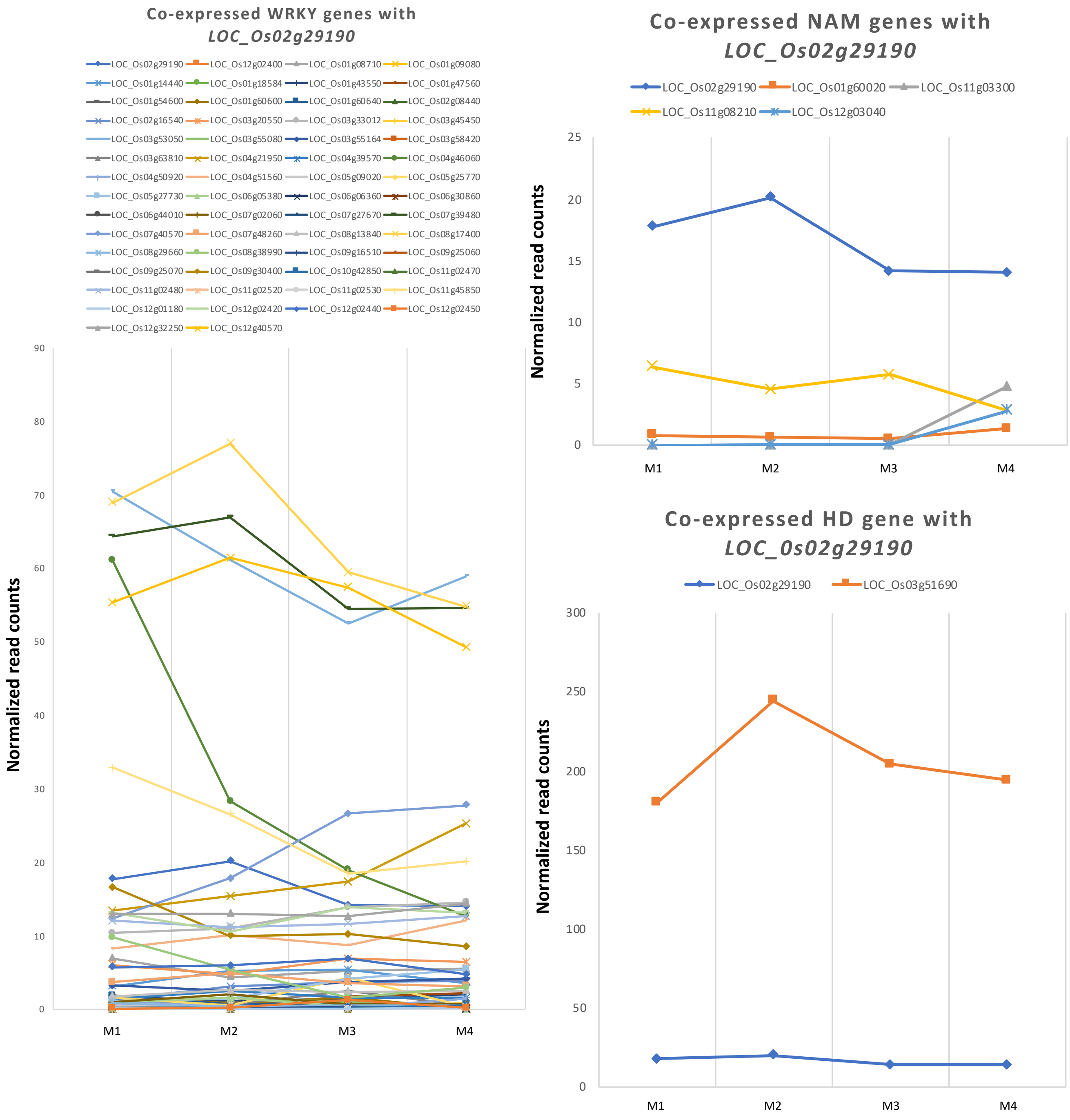
Co-expressed WRKY genes with
LOC_Os02g29190
LOC_Os02g29190
LOC_Os12g02400
LOC_Os01g08710
LOC_Os01g09080
LOC_Os01g14440
LOC_Os01g18584
LOC_Os01g43550
LOC_Os01g47560
LOC_Os01g54600
LOC_Os01g60600
LOC_Os01g60640
LOC_Os02g08440
LOC_Os02g16540
LOC_Os03g20550
LOC_Os03g33012
LOC_Os03g45450
LOC_Os03g53050
LOC_Os03g55080
LOC_Os03g55164
LOC_Os03g58420
LOC_Os03g63810
LOC_Os04g21950
LOC_Os04g39570
LOC_Os04g46060
LOC_Os04g50920
LOC_Os04g51560
LOC_Os05g09020
LOC_Os05g25770
LOC_Os05g27730
LOC_Os06g05380
LOC_Os06g06360
LOC_Os06g30860
LOC_Os06g44010
LOC_Os07g02060
LOC_Os07g27670
LOC_Os07g39480
LOC_Os07g40570
LOC_Os07g48260
LOC_Os08g13840
LOC_Os08g17400
LOC_Os08g29660
LOC_Os08g38990
LOC_Os09g16510
LOC_Os09g25060
LOC_Os09g25070
LOC_Os09g30400
LOC_Os10g42850
LOC_Os11g02470
LOC_Os11g02480
LOC_Os11g02520
LOC_Os11g02530
LOC_Os11g45850
LOC_Os12g01180
LOC_Os12g02420
LOC_Os12g02440
LOC_Os12g02450
LOC_Os12g32250
LOC_Os12g40570
Normalized read counts
0
10
20
30
40
50
60
70
80
90
M1
M2
M3
M4
Co-expressed NAM genes with
LOC_Os02g29190
LOC_Os02g29190
LOC_Os01g60020
LOC_Os11g03300
LOC_Os11g08210
LOC_Os12g03040
Normalized read counts
0
5
10
15
20
25
M1
M2
M3
M4
Co-expressed HD gene with
LOC_0s02g29190
LOC_Os02g29190
LOC_Os03g51690
Normalized read counts
0
50
100
150
200
250
300
M1
M2
M3
M4

D

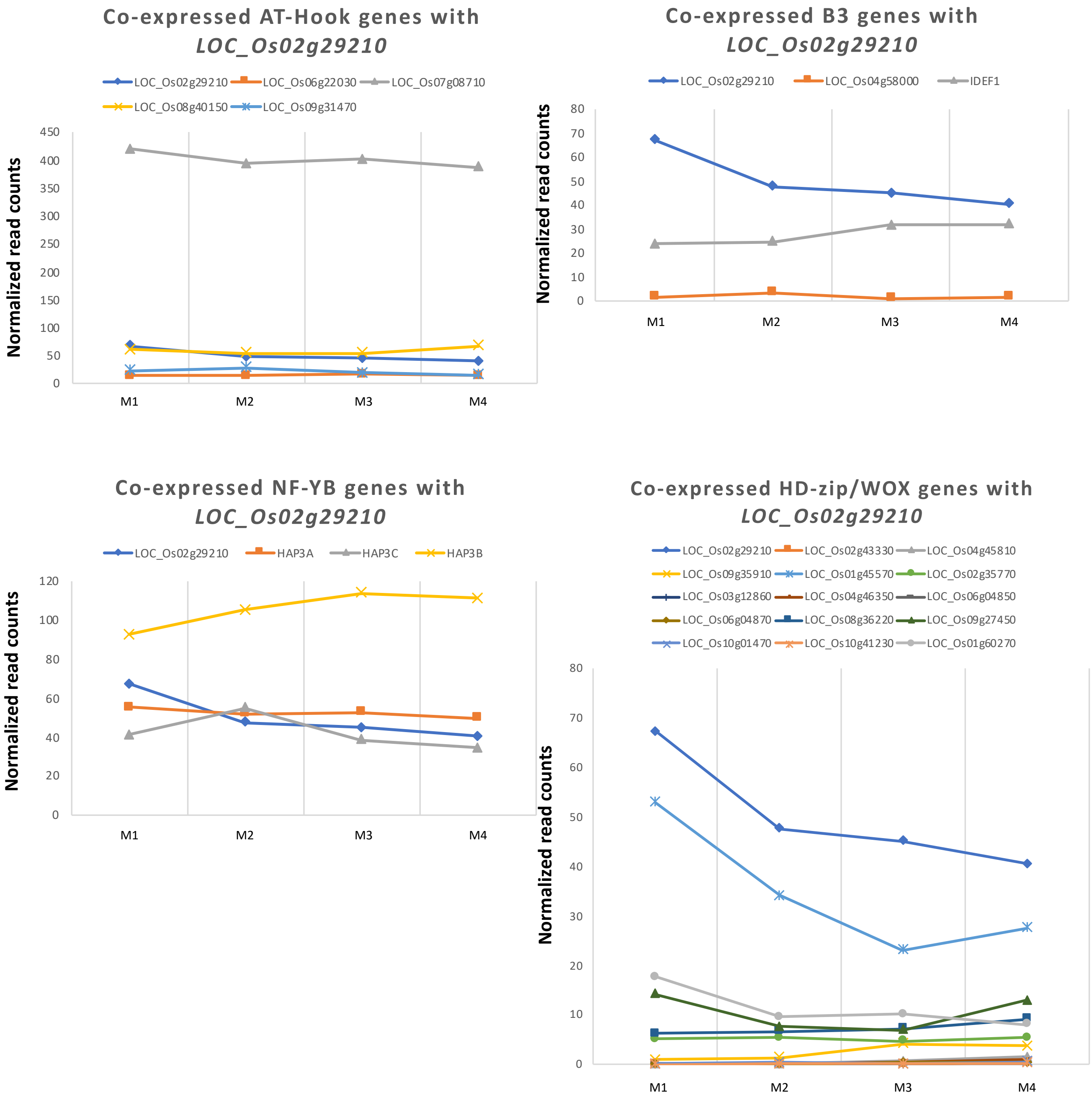

Co-expressed AT-Hook genes with *LOC_Os02g29210*
LOC_Os02g29210
LOC_Os06g22030
LOC_Os07g08710
LOC_Os08g40150
LOC_Os09g31470
Normalized read counts
450
400
350
300
250
200
150
100
50
0
M1
M2
M3
M4
Co-expressed B3 genes with *LOC_Os02g29210*
LOC_Os02g29210
LOC_Os04g58000
IDEF1
Normalized read counts
80
70
60
50
40
30
20
10
0
M1
M2
M3
M4
Co-expressed NF-YB genes with *LOC_Os02g29210*
LOC_Os02g29210
HAP3A
HAP3C
HAP3B
Normalized read counts
120
100
80
60
40
20
0
M1
M2
M3
M4
Co-expressed HD-zip/WOX genes with *LOC_Os02g29210*
LOC_Os02g29210
LOC_Os02g43330
LOC_Os04g45810
LOC_Os09g35910
LOC_Os01g45570
LOC_Os02g35770
LOC_Os03g12860
LOC_Os04g46350
LOC_Os06g04850
LOC_Os06g04870
LOC_Os08g36220
LOC_Os09g27450
LOC_Os10g01470
LOC_Os10g41230
LOC_Os01g60270
Normalized read counts
80
70
60
50
40
30
20
10
0
M1
M2
M3
M4
