# Supplementary material for: A cluster of Ankyrin and Ankyrin-TPR repeat genes is associated with panicle branching diversity in rice
Source: PLoS Genet. 2021 Jun 7;17(6):e1009594. doi: 10.1371/journal.pgen.1009594 (PMC8211194; doi:10.1371/journal.pgen.1009594)

ank1 single mutant *(LOC_Os02g29040)*

A

**Kitaake**	GTGCTACGCCACTGATTTATGCAGTTCTGGGTGGG...//...CGTGCTCCTCTCCATTTAGCAGTTGAACAAGG	
ank1-1	GTGCTACGCCACTGATTTATGCAGTTCTGGGTGGG...//...CGTGCTCCTCTC---TTAGCAGTTGAACAAGG	-3 bp
ank1-2	GTGCTACGCCACTGATTTATGCAGTTCTGGGTGGG...//...CGTGCTCCTCTC-ATTTAGCAGTTGAACAAGG	-1 bp
ank1-3	GTGCTACGCCACTGATTTATC------TGGGTGGG...//...CGTGCTCCTCTC--TTTAGCAGTTGAACAAGG	-8 bp

B

Kitaake
ank1-1
ank1-2
ank1-3

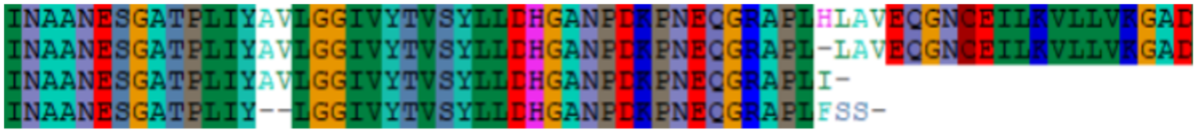

-1 aa
-177 aa
-177 aa

C

Kitaake
ank1-1

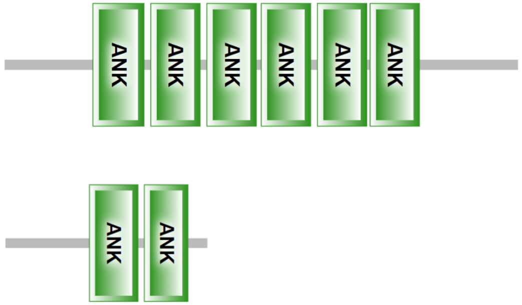

ank1-2
ank1-3

ank2 single mutant *(LOC_Os02g29210)*

A

**Kitaake**	TTGTGTGGACACAGTCGCAAACCGTGGCACACCACTTCATGTTGCTGCTAGTAAAGGGAAAGATGGTGCTATGAAGATTTTATTGGA
ank2-1	TTGTGTGGACACAGTCGCAAA--ATTGGA -60 bp
ank2-2	TTGTGTGGACACAGTCGCAAA---TATTGGA -59 bp
ank2-3	TTGTG--TGGA -78 bp

B

Kitaake	GTVQLLLAKGACVDTVANRGTPLHVAASKGKDGAMKILLDHNADFNKMVDGHL	
ank2-1	GTVQLLLAKGACVDTVAK--------------------LDHNADFNKMVDGHL	-20 aa
ank2-2	GTVQLLLAKGACVDTVANIGPQCRF-	-278 aa
ank2-3	GTVQLLLAKGACVD--------------------------HNADFNKMVDGHL	-26 aa

C

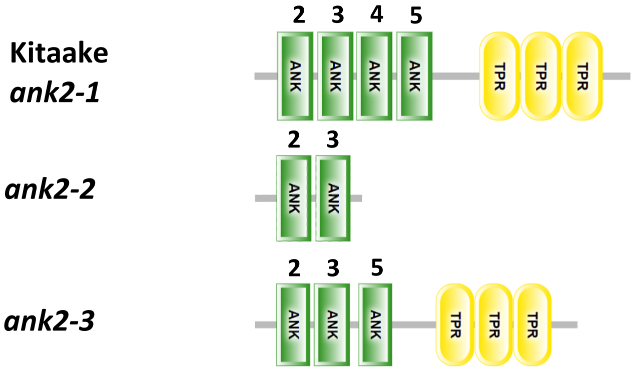

ank1 ank2 double mutant

A

LOC_Os02g29040

**Kitaake**	GTGCTACGCCACTGATTTATGCAGTTCTGGGTGGG...//...CGTGCTCCTCTCCATTTAGCAGTTGAACAAGG	
ank1ank2_1	GTGCTACGCCACTGATTTATGCAGTTCTGGGTGGG...//...CGTGCTCCTCTC----TAGCAGTTGAACAAGG	-4 bp

LOC_Os02g29210

**Kitaake**	TTGTGTGGACACAGTCGCAAACCGTGGCACACCACTTCATGTTGCTGCTAGTAAAGGGAAAGATGGTGCTATGAAGATTTTATTGGA	
ank1ank2_1	TTGTGTGG----------------ACACCACTTCATGTTGCTGCTAGTAAAGGGAAAGATGGTGCTATGAAGATTTTATTGGA	-20 bp

B

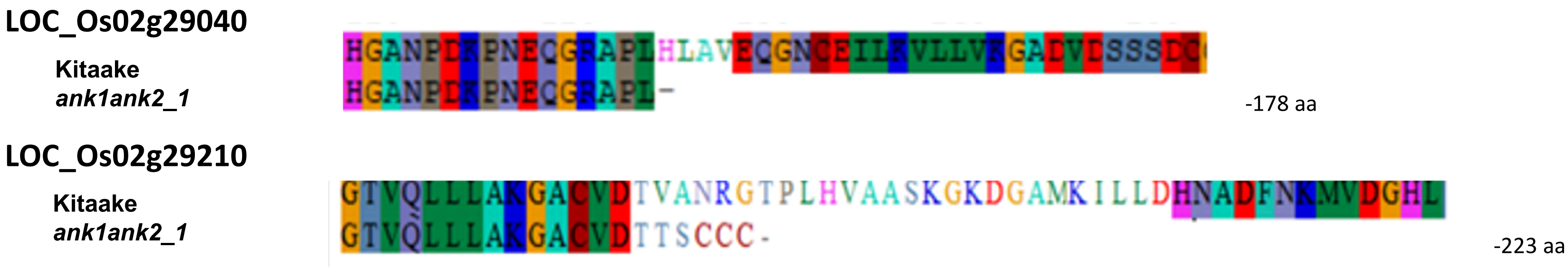

C

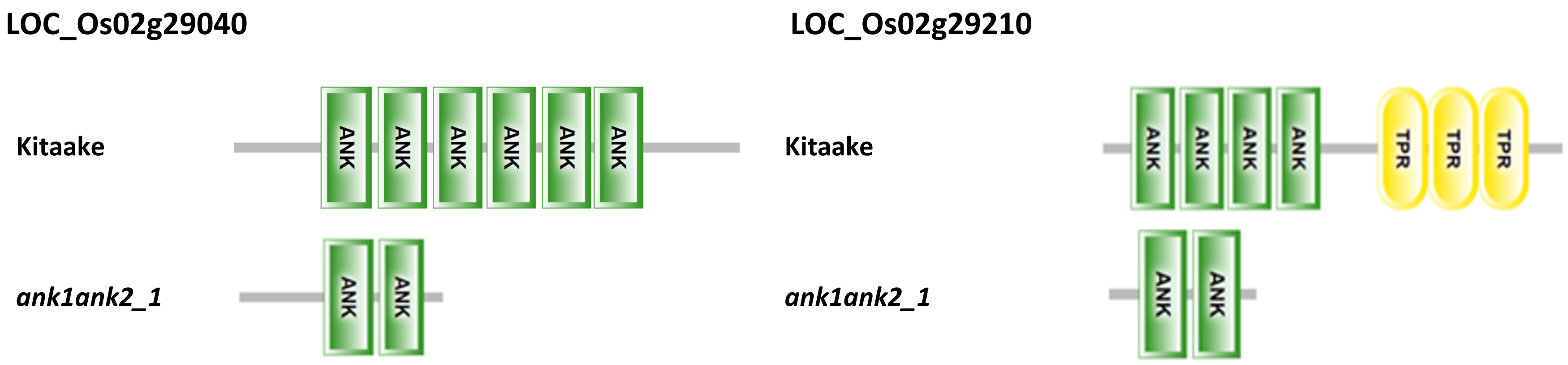

Supplement: S15 Fig — (A) Alignment of the nucleic sequence of wild-type Kitaake with the different CRISPR-Cas9-induced alleles. (B) Alignment of the deduced amino acid sequence of the wild-type Kitaake, with those of the different CRISPR-Cas9-induced alleles. (C) Protein structure of the wild-type Kitaake form and the different CRISPR-Cas9-induced alleles. (PDF) [file pgen.1009594.s015.pdf]
