# Supplementary material for: A cluster of Ankyrin and Ankyrin-TPR repeat genes is associated with panicle branching diversity in rice
Source: PLoS Genet. 2021 Jun 7;17(6):e1009594. doi: 10.1371/journal.pgen.1009594 (PMC8211194; doi:10.1371/journal.pgen.1009594)
Supplement: S1 Table — Zone: Vietnamese region where the accession was originating (MRD = Mekong River Delta; SE = Southeast; CH = Central Highlands; SCC = South Central Coast; NCC = North Central Coast; RRD = Red River Delta; NW = Northwest; NE = Northeast; u = unknown); Group: genetic group of the accessions according to [68] for indica (I1 to I6) and admixture (m, Im, Jm); Ecosystem: ecosystem from where the accession originates (RL = rainfed lowland; UP = upland; u = unknown). (DOCX) [file pgen.1009594.s019.docx]

| **Genotype** | **Name** | **Province** | **Zone** | **Ecosystem** | **Group** | **Haplotype** |
| --- | --- | --- | --- | --- | --- | --- |
| G6 | SOM GIAI HUNG YEN | u | u | u | I4 | H1 |
| G8 | CHON TU 502 HOC VIEN | u | u | u | I4 | H1 |
| G9 | LOC TRANG SOM PLAY CAU | u | u | u | I4 | H1 |
| G12 | TAM CAO VINH PHUC | VINH PHUC | RRD | u | I4 | H1 |
| G17 | NEP GA GAY HAI DUONG | HAI DUONG | RRD | u | Im | H1 |
| G19 | ON | HA NOI | RRD | u | Im | H1 |
| G95 | LUA CHAM | NAM DINH | RRD | RL | I4 | H1 |
| G105 | NEP THAI LAN | HA GIANG AND TUYEN QUANG | NE | u | I3 | H2 |
| G153 | TE NUONG | THANH HOA AND SON LA | NW | UP | I3 | H2 |
| G155 | KHAU PE LANH | SON LA | NW | UP | I3 | H2 |
| G189 | KHAU NAM RINH | DIEN BIEN | NW | UP | I3 | H2 |
| G205 | BLE BLAU CHO | SON LA | NW | UP | I3 | H2 |
