# Supplementary material for: A cluster of Ankyrin and Ankyrin-TPR repeat genes is associated with panicle branching diversity in rice
Source: PLoS Genet. 2021 Jun 7;17(6):e1009594. doi: 10.1371/journal.pgen.1009594 (PMC8211194; doi:10.1371/journal.pgen.1009594)
Supplement: S2 Table — Relevant information relating to the detection of polymorphic sites within genes and their promoter regions and their impact on protein sequences. Also shown is information on the expression of these genes during early stages of panicle development, based on publicly available data set and local RNA seq dataset. Applicable categories are indicated by green cells. (DOCX) [file pgen.1009594.s020.docx]

| **MSU_Locus name** | **coord_ 1** | **coord_ 2** | **RAPdb_Locus** | **MSU annotation** | **Captured** | **SNP**  **INDEL** | **Prot pol?** | **Prom pol?** | **panicle exp** |
| --- | --- | --- | --- | --- | --- | --- | --- | --- | --- |
| LOC_Os02g27990 | 16575001 | 16575201 | none | expressed protein |  |  |  |  |  |
| LOC_Os02g28030 | 16596658 | 16598721 | Os02g0481400 | expressed protein |  |  |  |  |  |
| LOC_Os02g28040 | 16602668 | 16612238 | Os02g0481700 | lipase putative expressed |  |  |  |  |  |
| LOC_Os02g28050 | 16615213 | 16616305 | none | hypothetical protein |  |  |  |  |  |
| LOC_Os02g28074 | 16617378 | 16633950 | Os02g0481900 | XRN 5-3 exonuclease N-terminus family protein expressed |  |  |  |  |  |
| LOC_Os02g28100 | 16638284 | 16638490 | none | expressed protein |  |  |  |  |  |
| LOC_Os02g28110 | 16639666 | 16641280 | Os02g0482400 | ornithine decarboxylase putative expressed |  |  |  |  |  |
| LOC_Os02g28130 | 16660894 | 16661495 | Os02g0483000 | fasciclin-like arabinogalactan protein 8 precursor putative expressed |  |  |  |  |  |
| LOC_Os02g28140 | 16664107 | 16665216 | Os02g0483300 | expressed protein | **no** |  |  |  |  |
| LOC_Os02g28160 | 16673466 | 16674141 | none | expressed protein |  |  |  |  |  |
| LOC_Os02g28170 | 16678144 | 16680057 | Os02g0483500 | transferase family protein putative expressed |  |  |  |  |  |
| LOC_Os02g28200 | 16691918 | 16693524 | Os02g0483800 | transferase family protein putative expressed |  |  |  |  |  |
| LOC_Os02g28210 | 16699311 | 16699598 | Os02g0484000 | expressed protein |  |  |  |  |  |
| LOC_Os02g28220 | 16702131 | 16703949 | Os02g0484200 | transferase family protein putative expressed |  |  |  |  |  |
| LOC_Os02g28230 | 16706938 | 16707243 | none | expressed protein | **no** |  |  |  |  |
| LOC_Os02g28240 | 16707837 | 16709086 | none | transferase family protein putative expressed | **no** |  |  |  |  |
| LOC_Os02g28270 | 16718751 | 16719083 | Os02g0484480 | expressed protein |  |  |  |  |  |
| LOC_Os02g28280 | 16721215 | 16722896 | Os02g0484690 | expressed protein |  |  |  |  |  |
| LOC_Os02g28290 | 16723633 | 16724146 | none | hypothetical protein |  |  |  |  |  |
| LOC_Os02g28300 | 16726375 | 16727995 | none | transferase family protein putative expressed |  |  |  |  |  |
| LOC_Os02g28310 | 16732589 | 16734631 | none | expressed protein |  |  |  |  |  |
| LOC_Os02g28334 | 16747537 | 16748322 | Os02g0484900 | expressed protein |  |  |  |  |  |
| LOC_Os02g28340 | 16750077 | 16751771 | Os02g0485000 | transferase family protein putative expressed |  |  |  |  |  |
| LOC_Os02g28360 | 16762396 | 16762677 | none | hypothetical protein |  |  |  |  |  |
| LOC_Os02g28410 | 16805941 | 16807323 | Os02g0485800 | transferase family protein putative expressed |  |  |  |  |  |
| LOC_Os02g28430 | 16811341 | 16811712 | none | expressed protein |  |  |  |  |  |
| LOC_Os02g28450 | 16824620 | 16825069 | none | expressed protein |  |  |  |  |  |
| LOC_Os02g28460 | 16830245 | 16831209 | none | expressed protein |  |  |  |  |  |
| LOC_Os02g28465 | 16831620 | 16834298 | none | expressed protein |  |  |  |  |  |
| LOC_Os02g28470 | 16835055 | 16836519 | Os02g0486500 | transferase family protein putative expressed |  |  |  |  |  |
| LOC_Os02g28490 | 16848963 | 16850355 | none | expressed protein |  |  |  |  |  |
| LOC_Os02g28530 | 16881441 | 16883694 | none | expressed protein |  |  |  |  |  |
| LOC_Os02g28560 | 16903864 | 16904896 | Os02g0487166 | hypothetical protein |  |  |  |  |  |
| LOC_Os02g28570 | 16907714 | 16912612 | none | expressed protein |  |  |  |  |  |
| LOC_Os02g28580 | 16915529 | 16919296 | Os02g0487300 | expressed protein |  |  |  |  |  |
| LOC_Os02g28590 | 16922389 | 16923061 | none | expressed protein |  |  |  |  |  |
| LOC_Os02g28600 | 16924023 | 16925375 | Os02g0487701 | OsFBX50 - F-box domain containing protein expressed |  |  |  |  |  |
| LOC_Os02g28660 | 16959497 | 16961622 | Os02g0487900 | expressed protein |  |  |  |  |  |
| LOC_Os02g28670 | 16962226 | 16963868 | none | expressed protein |  |  |  |  |  |
| LOC_Os02g28680 | 16966471 | 16968339 | Os02g0488100 | expressed protein |  |  |  |  |  |
| LOC_Os02g28700 | 16971289 | 16971818 | none | expressed protein |  |  |  |  |  |
| LOC_Os02g28720 | 16981477 | 16985654 | Os02g0488600 | spotted leaf 11 putative expressed |  |  |  |  |  |
| LOC_Os02g28730 | 17000884 | 17001453 | Os02g0488900 | expressed protein |  |  |  |  |  |
| LOC_Os02g28760 | 17019663 | 17019926 | none | expressed protein | **no** |  |  |  |  |
| LOC_Os02g28800 | 17033834 | 17034356 | none | hypothetical protein |  |  |  |  |  |
| LOC_Os02g28810 | 17035682 | 17037903 | Os02g0489400 | ribosomal protein putative expressed |  |  |  |  |  |
| LOC_Os02g28820 | 17039977 | 17041355 | *Os02g0489500* | expressed protein |  |  |  |  |  |
| LOC_Os02g28830 | 17046461 | 17052031 | Os02g0489550 | tetratricopeptide repeat domain containing protein expressed |  |  |  |  |  |
| LOC_Os02g28850 | 17074201 | 17082223 | Os02g0489800 | Kinesin motor domain domain containing protein expressed |  |  |  |  |  |
| LOC_Os02g28860 | 17083359 | 17083907 | none | expressed protein |  |  |  |  |  |
| LOC_Os02g28870 | 17085385 | 17088926 | Os02g0490000 | U-box domain-containing protein putative expressed |  |  |  |  |  |
| LOC_Os02g28880 | 17091188 | 17092349 | none | hypothetical protein |  |  |  |  |  |
| LOC_Os02g28900 | 17118767 | 17120699 | *Os02g0490500* | cytokinin-O-glucosyltransferase 2 putative expressed |  |  |  |  |  |
| LOC_Os02g28910 | 17125267 | 17125752 | none | expressed protein |  |  |  |  |  |
| LOC_Os02g28930 | 17132537 | 17132977 | none | expressed protein |  |  |  |  |  |
| LOC_Os02g28970 | 17150510 | 17153815 | Os02g0491300 | expressed protein |  |  |  |  |  |
| LOC_Os02g28980 | 17156430 | 17160895 | Os02g0491400 | peptidyl-prolyl isomerase putative expressed |  |  |  |  |  |
| LOC_Os02g28990 | 17163807 | 17164531 | Os02g0491500 | expressed protein |  |  |  |  |  |
| LOC_Os02g29000 | 17165188 | 17165997 | Os02g0491600 | Cupin domain containing protein expressed |  |  |  |  |  |
| LOC_Os02g29020 | 17175222 | 17175893 | Os02g0491800 | Cupin domain containing protein expressed |  |  |  |  |  |
| LOC_Os02g29040 | 17196088 | 17204631 | Os02g0492000 | ankyrin repeat domain containing protein putative expressed |  |  |  |  |  |
| LOC_Os02g29070 | 17217534 | 17218463 | Os02g0492300 | protein kinase domain containing protein expressed |  |  |  |  |  |
| LOC_Os02g29080 | 17220247 | 17220789 | none | conserved hypothetical protein |  |  |  |  |  |
| LOC_Os02g29090 | 17223614 | 17227328 | Os02g0492500 | expressed protein |  |  |  |  |  |
| LOC_Os02g29110 | 17235539 | 17236295 | Os02g0492600 | ankyrin repeat family protein putative expressed |  |  |  |  |  |
| LOC_Os02g29120 | 17244391 | 17244732 | Os02g0492600 | expressed protein |  |  |  |  |  |
| LOC_Os02g29130 | 17245021 | 17254879 | Os02g0492900 | ankyrin putative expressed |  |  |  |  |  |
| LOC_Os02g29140 | 17257940 | 17266066 | Os02g0493050 | ankyrin putative expressed |  |  |  |  |  |
| LOC_Os02g29150 | 17271183 | 17275865 | Os02g0493100 | OsFBO11 - F-box and other domain containing protein expressed |  |  |  |  |  |
| LOC_Os02g29160 | 17277253 | 17290200 | Os02g0493300 | ankyrin putative expressed |  |  |  |  |  |
| LOC_Os02g29170 | 17293924 | 17295905 | none | expressed protein |  |  |  |  |  |
| LOC_Os02g29180 | 17300829 | 17301347 | none | expressed protein | **no** |  |  |  |  |
| LOC_Os02g29190 | 17309120 | 17316341 | Os02g0494000 | ankyrin putative expressed |  |  |  |  |  |
| LOC_Os02g29200 | 17317871 | 17318311 | none | expressed protein |  |  |  |  |  |
| LOC_Os02g29210 | 17327565 | 17335614 | Os02g0494400 | ankyrin putative expressed |  |  |  |  |  |
| LOC_Os02g29220 | 17337595 | 17342087 | Os02g0494600 | expressed protein |  |  |  |  |  |
| LOC_Os02g29230 | 17354840 | 17362939 | Os02g0494700 | expressed protein |  |  |  |  |  |
