# Supplementary material for: A cluster of Ankyrin and Ankyrin-TPR repeat genes is associated with panicle branching diversity in rice
Source: PLoS Genet. 2021 Jun 7;17(6):e1009594. doi: 10.1371/journal.pgen.1009594 (PMC8211194; doi:10.1371/journal.pgen.1009594)
Supplement: S4 Table — (DOCX) [file pgen.1009594.s022.docx]

| **Name** | **Gene** | **Sequence (5'-3')** | **Purpose** |
| --- | --- | --- | --- |
| RM151_F |  | GGCTGCTCATCAGCTGCATGCG | Genotyping using SSR markers |
| RM151_R |  | TCGGCAGTGGTAGAGTTTGATCTGC |  |
| RM180_F |  | CTACATCGGCTTAGGTGTAGCAACACG | Genotyping using SSR markers |
| RM180_R |  | ACTTGCTCTACTTGTGGTGAGGGACTG |  |
| RM204_F |  | GTGACTGACTTGGTCATAGGG | Genotyping using SSR markers |
| RM204_R |  | GCTAGCCATGCTCTCGTACC |  |
| RM289_F |  | TTCCATGGCACACAAGCC | Genotyping using SSR markers |
| RM289_R |  | CTGTGCACGAACTTCCAAAG |  |
| RM320_F |  | CAACGTGATCGAGGATAGATC | Genotyping using SSR markers |
| RM320_R |  | GGATTTGCTTACCACAGCTC |  |
| RM400_F |  | ACACCAGGCTACCCAAACTC | Genotyping using SSR markers |
| RM400_R |  | CGGAGAGATCTGACATGTGG |  |
| RM410_F |  | GCTCAACGTTTCGTTCCTG | Genotyping using SSR markers |
| RM410_R |  | GAAGATGCGTAAAGTGAACGG |  |
| RM491_F |  | ACATGATGCGTAGCGAGTTG | Genotyping using SSR markers |
| RM491_R |  | CTCTCCCTTCCCAATTCCTC |  |
| RM532_F |  | TCTATAATGTAGCCCCCCCC | Genotyping using SSR markers |
| RM532_R |  | TTTCAGGGGCTTCTACCAAC |  |
| RM535_F |  | ACTACATACACGGCCCTTGC | Genotyping using SSR markers |
| RM535_R |  | CTACGTGGACACCGTCACAC |  |
| RM577_F |  | GCTTTCCCTCTAACCCCTCT | Genotyping using SSR markers |
| RM577_R |  | GGATGTACCGCTGACATGAA |  |
| RM592_F |  | TCTTTGGTATGAGGAACACC | Genotyping using SSR markers |
| RM592_R |  | AGAGATCCGGTTTGTTGTAA |  |
| Actin_F | LOC_Os03g50885 | CATTCCAGCAGATGTGGATTG | qRT-PCR |
| Actin-R |  | TCTTGGCTTAGCATTCTTGG |  |
| 28040_F | LOC_Os02g28040 | GACTGAGGAATGCCTGGAACAAGG | qRT-PCR |
| 28040_R |  | CAAATGTGGCACTATGCTATGGACTACC |  |
| 28340_F | LOC_Os02g28340 | CGACGCCATGGCTTGGTTTTCC | qRT-PCR |
| 28340_R |  | ACATACATGTGTGGATTCATCCCAGG |  |
| SL11_F | LOC_Os02g28720 | GCTGGGTGCGTCGGATATGT | qRT-PCR |
| SL11_R |  | GTGCTTCAGACTTCATGATCAGGTAGAA |  |
| 28810_F | LOC_Os02g28810 | CCAACAGGGACGCACACTTG | qRT-PCR |
| 28810_R |  | GCACCCTTGCCCTTCTTCCT |  |
| 28830_R | LOC_Os02g28830 | GATGCAGCCTGTCGTGCTGT | qRT-PCR |
| 28830_F |  | TGACATCGCGGCAACACCTC |  |
| 28850_F | LOC_Os02g28850 | CATCCCTCGCTGCCTAGTGC | qRT-PCR |
| 28850_R |  | GACTCATCAACTCTCCAACTGCCC |  |
| 28980_F | LOC_Os02g28980 | CGGCTGTAGAGGTCCGTCTG | qRT-PCR |
| 28980_R |  | TCACTCGAACGTGTCTAGCTCACAG |  |
| 29040_F | LOC_Os02g29040 | CTCGTGCTGCCTGAATTACCTGTC | qRT-PCR |
| 29040_R |  | TACAAAGCCGTGCCAGTTGC |  |
| 29130_F2 | LOC_Os02g29130 | CGTCTCGTGCTGCTTGAATTGC | qRT-PCR |
| 29130_R |  | CGGTGTAATTGATACTAGCTGGTGGT |  |
| 29140_F | LOC_Os02g29140 | GCATCTTTCTGCTTTCTGCC | qRT-PCR |
| 29140_R |  | ACACCCTTCACATCAGCACC |  |
| 29150_F2 | LOC_Os02g29150 | GCAGCCTCCGCATCTTGC | qRT-PCR |
| 29150_R2 |  | GCTCCATGAATCCCAGCGACC |  |
| 29160_F | LOC_Os02g29160 | CGAGCAAATGTGTGCCGAACTC | qRT-PCR |
| 29160_R |  | GATCAGCCTTGTGACCTCCACG |  |
| 29190_F5 | LOC_Os02g29190 | GCACTGTACCTTTACCCTGATGATGC | qRT-PCR |
| 29190_R5 |  | GTAACATCAGAGCAGCACCCAGC |  |
| 29210_F | LOC_Os02g29210 | GCTCTGGTCCTCTCCTACACG | qRT-PCR _ *in situ* hybridization |
| 29210_R |  | CCGTGATGTTCAACAGTCACCACC |  |
| 29220_F | LOC_Os02g29220 | ACGGACATGCAACCGGAGAA | qRT-PCR |
| 29220_R |  | CGGAGGGCCTTCAGCAATCT |  |
| ANK1_F1 | LOC_Os02g29040 | CCACGGGTTCCTCGTCTCTTCC | CDS cloning |
| ANK1_R1 |  | GACAGGTAATTCAGGCAGCACGAG |  |
| ANK1_F2 | LOC_Os02g29040 | CATGGCGCCGCCTCACGC | CDS cloning |
| ANK1_R2 |  | TTTATGCCCTACTTCCTTCTAACTTCAGATC |  |
| ANK1_F2_attB1 | LOC_OS02G29040 | GGGGACAAGTTTGTACAAAAAAGCAGGCTCATGGCGCCGCCTCACGC | CDS cloning |
| ANK1_R2_attB2 |  | GGGGACCACTTTGTACAAGAAAGCTGGGTTTTTATGCCCTACTTCCTTCTAACTTCAGATC |  |
| ANK2_F1 | LOC_Os02g29210 | CGACTAACTCGATCTGCTCCTCGC | CDS cloning |
| ANK2_R1 |  | CCGTGATGTTCAACAGTCACCACC |  |
| ANK2_F2 | LOC_Os02g29210 | AATGGTGGAGAAGTTGCTCTTCG | CDS cloning |
| ANK2_R2 |  | TTCACCTAGCCTCTGTGCTTGC |  |
| ANK2_F2-attB1 | LOC_Os02g29210 | GGGGACAAGTTTGTACAAAAAAGCAGGCTAATGGTGGAGAAGTTGCTCTTCG | CDS cloning |
| ANK2_R2-attB2 |  | GGGGACCACTTTGTACAAGAAAGCTGGGTTTTCACCTAGCCTCTGTGCTTGC |  |
| pUBI |  | GGATGATGGCATATGCAGCAG | pC5300 plasmids sequencing |
| pCaMV35S |  | TGACAGATAGCTGGGCAATG |  |
| ANK1-S1 | LOC_Os02g29040 | GTGACCTCCGCCTCTTCA |  |
| ANK1-AS1 |  | TGCCCTACTTCCTTCTAACTTC |  |
| ANK2-S2 | LOC_Os02g29210 | ACAGACGGAAAGATGTGGAGA |  |
| ANK2-AS2 |  | AAATCAGTGGCGTAGCACCT |  |
| HPT-OE-F |  | TTCAGCTTCGATGTAGGAGG | T-DNA copy number in OX lines by RT-qPCR |
| HPT-OE-R |  | AGAAGAAGATGTTGGCGACC |  |
| SPS-F | U33175 | TTGCGCCTGAACGGATAT | T-DNA copy number in OX lines by RT-qPCR |
| SPS-R |  | CGGTTGATCTTTTCGGGATG |  |
| CDS-ANK1-F | LOC_Os02g29040 | CGCCAACGAGTCAGGTGCTA | Gene expression analysis in OX-ANK1 lines_ *in situ* hybridization |
| CDS-ANK1-R |  | TGGCGTCCCACAGTCAGAAG |  |
| CDS-ANK2-F | LOC_Os02g29210 | AACCGTGGCACACCACTTCA | expression analysis in OX-ANK2 lines |
| CDS-ANK2-R |  | GAGCCACCCTTTGCAGCAGT |  |
| pU3-F |  | GACCATGATTACGCCAAGCTTAAGGAATCTTTAAACATACG | Detection of U3:PTG |
| gRNA-R |  | GGACCTGCAGGCATGCACGCGCTAAAAACGGACTAGC |  |
| pUBI-F |  | GCTTGTGCGTTTCGATTTGA | Detection of Cas9 gene |
| Cas9-R |  | CCGCTCGTGCTTCTTATCCT |  |
| HPT-F |  | GCTCCAGTCAATGACCGCTG | Hygromycin phosphotransferase II detection |
| HPT-R |  | CTCGGAGGGCGAAGAATCTC |  |
| ANK1_Ex4_F1 | LOC_Os02g29040 | CACGTTATCAACAAACCTCCTATC | Genotyping and sequencing of ank1 lines |
| ANK1_Ex4_R1 |  | CTGACTATAGAACACAGACTTTCG |  |
| ANK2_Ex4_F2 | LOC_Os02g29210 | CAGTTATCACGGTTATCATGTGC | Genotyping and sequencing of ank2 lines |
| ANK2_Ex4_R2 |  | CGTAATCTTCATGCTTGGAAC |  |
| ANK1a-F | LOC_Os02g29040 | TAGGTCTCCGATTTATGCAGTTCGTTTTAGAGCTAGAA | PTG synthesis_CRISPR-Cas9 |
| ANK1a-R |  | ATGGTCTCAAATCAGTGGCTGCACCAGCCGGGAA |  |
| ANK1b-F |  | TAGGTCTCCCTGCTAAATGGAGGTTTTAGAGCTAGAA |  |
| ANK1b-R |  | ATGGTCTCAGCAGTTGAACATGCACCAGCCGGGAA |  |
| ANK2c-F | LOC_Os02g29210 | TAGGTCTCCACAGTCGCAAACCGGTTTTAGAGCTAGAA | PTG synthesis_CRISPR-Cas9 |
| ANK2c-R |  | ATGGTCTCACTGTGTCCACTGCACCAGCCGGGAA |  |
| ANK2d-F |  | TAGGTCTCCTATGAAGATTTTATGTTTTAGAGCTAGAA |  |
| ANK2d-R |  | ATGGTCTCACATAGCACCATGCACCAGCCGGGAA |  |
| HIS_160-F | LOC_Os02g29160 | ATTTGCTGTGCGATGTGGTA | *in situ* hybridization |
| HIS_160-R |  | AGGCAAAGCGCCTTAGTGTA |  |
| 29190_F10 | LOC_Os02g29190 | GTTCTGGTCCTCCTCCACGT | *in situ* hybridization |
| 29190_R13 |  | AGGGCACAAGATTAACTTCG |  |
