# Supplementary material for: A cluster of Ankyrin and Ankyrin-TPR repeat genes is associated with panicle branching diversity in rice
Source: PLoS Genet. 2021 Jun 7;17(6):e1009594. doi: 10.1371/journal.pgen.1009594 (PMC8211194; doi:10.1371/journal.pgen.1009594)
Supplement: S5 Table — The names of ANK-TPR genes according to [10] are indicated, as well as the features of the gene and its encoded protein (number of introns, protein, protein length, protein molecular weight, PI). The predicted sub-cellular localization is indicated according to [10]. O. sativa genes mentioned in [10] but not maintained in the MSU7.0 version of the O. sativa genome are indicated in grey. The presence of O. rufipogon orthologs is indicated for the corresponding O. sativa genes. O. sativa genes from QTL_9 are highlighted in orange. (DOCX) [file pgen.1009594.s023.docx]

| **Species** | **Locus name** | **Sub-family** | **TRP name** | **Chrom.** | **nb intron** | **prot. length** | **pI** | **MW** | **sub cell loc prediction** | ***O. rufipogon*** | **Remarks** |
| --- | --- | --- | --- | --- | --- | --- | --- | --- | --- | --- | --- |
| ***O. sativa*** | LOC_Os01g07640 | ANK-TPR | OsTPR002 | 1 | 10 | 535 | 6.47 | 57162.31 | none | ORUFI01G04820 |  |
|  | LOC_Os01g07980 | ANK-TPR | OsTPR003 | 1 | 7 | 281 | 8.13 | 31515.34 | none | ORUFI01G05130 |  |
|  | *LOC_Os02g29030* | *ANK-TPR* | *OsTPR041* | *2* | *14* | *1669* | *6.67* | *183974.57* | *none* |  | *Not in MSU7.0* |
|  | LOC_Os02g29040 | ANK |  | 2 | 11 | 303 | 6.59 | 32418.3 | none | ORUFI02G17950 | *O. rufipogon* annotation covers the *LOC_Os02g29030* and *LOC_Os02g29040* genes |
|  | LOC_Os02g29130 | ANK-TPR | OsTPR042 | 2 | 10 | 381 | 6.79 | 41738.38 | plastid? | ORUFI02G18010 | *O. rufipogon* annotation covers the LOC_Os02g29130, LOC_Os02g29140 and LOC_Os02g291160 *O. sativa* genes |
|  | LOC_Os02g29140 | ANK-TPR | OsTPR043 | 2 | 11 | 442 | 6.46 | 47806.97 | mito? | ORUFI02G18010 | *O. rufipogon* annotation covers the LOC_Os02g29130, LOC_Os02g29140 and LOC_Os02g291160 *O. sativa* genes |
|  | LOC_Os02g29160 | ANK-TPR | OsTPR045 | 2 | 11 | 447 | 6.34 | 48150.38 | mito?chloro? | ORUFI02G18010 | *O. rufipogon* annotation covers the LOC_Os02g29130, LOC_Os02g29140 and LOC_Os02g291160 *O. sativa* genes |
|  | LOC_Os02g29190 | ANK-TPR | OsTPR046 | 2 | 9 | 357 | 5.19 | 39109.55 | mito? | ORUFI02G18040 |  |
|  | LOC_Os02g29210 | ANK-TPR | OsTPR047 | 2 | 9 | 371 | 6.07 | 39783.22 | none | ORUFI02G18060 |  |
|  | LOC_Os03g42350 | ANK-TPR | OsTPR090 | 3 | 10 | 397 | 5.18 | 43497.66 | none | ORUFI03G27240 |  |
|  | *LOC_Os03g42620* | *ANK-TPR* | *OsTPR091* | *3* | *11* | *485* | *5.81* | *51318.13* | *mito?* |  | *Not in MSU7.0* |
|  | LOC_Os03g42650 | ANK-TPR | OsTPR092 | 3 | 11 | 472 | 7.9 | 50188.34 | mito? | ORUFI03G27450 |  |
|  | *LOC_Os03g42686* | *ANK-TPR* | *OsTPR093* | *3* | *13* | *541* | *6.04* | *59386.11* | *none* |  | *Not in MSU7.0* |
|  | *LOC_Os03g42720* | *ANK-TPR* | *OsTPR094* | *3* | *9* | *441* | *8.17* | *48311.17* | *none* |  | *Shorter protein in MSU7.0* |
|  | LOC_Os05g01310 | ANK-TPR | OsTPR116 | 5 | 12 | 460 | 5.37 | 49067.22 | none | ORUFI05G00250 |  |
|  | LOC_Os08g13640 | ANK-TPR | OsTPR161 | 8 | 11 | 384 | 4.84 | 41620.07 | mito?chloro? | ORUFI08G07860 | Problem of annotation in O. rufipogon? |
|  | LOC_Os09g03630 | ANK-TPR | OsTPR171 | 9 | 10 | 428 | 6.71 | 46741.54 | none | ORUFI09G01110 |  |
|  | LOC_Os09g03680 | ANK-TPR | OsTPR172 | 9 | 9 | 405 | 6.4 | 43707.14 | none | ORUFI09G01140 |  |
|  | LOC_Os09g03750 | ANK-TPR | OsTPR173 | 9 | 10 | 439 | 6.6 | 47728.93 | none | ORUFI09G01190 |  |
|  | LOC_Os12g40770 | ANK-TPR | OsTPR211 | 12 | 10 | 423 | 5.84 | 46223.14 | none | ORUFI12G19940 | Problem of annotation in O. rufipogon? |
|  | LOC_Os12g40780 | ANK-TPR | OsTPR212 | 12 | 11 | 387 | 6.64 | 42306.63 | none | ORUFI12G19950 |  |
|  | LOC_Os12g43940 | ANK-TPR | OsTPR215 | 12 | 11 | 429 | 5.46 | 47073.47 | none | ORUFI12G22290 |  |
|  | *LOC_Os12g43841* | *ANK-TPR* | *OsTPR216* | *12* | *11* | *439* | *5.43* | *48529.16* | *none* |  | *not in MSU7.0* |
|  |  |  |  |  |  |  |  |  |  |  |  |
| ***A. thaliana*** | At3g04710 | ANK-TPR | AtTPR071 | 3 | 14 | 680 | 5.9 | 74009.54 | mito? |  |  |
|  |  |  |  |  |  |  |  |  |  |  |  |
| ***Z. mays*** | GRMZM2g155314 | ANK-TPR | ZmTPR021 | 1 | 10 | 417 | 5.56 | 44843.61 | plastid?chloro? |  |  |
|  | GRMZM2g099366 | ANK-TPR | ZmTPR023 | 1 | 8 | 269 | 5.26 | 30492.78 | none |  |  |
|  | GRMZM2g098212 | ANK-TPR | ZmTPR180 | 8 | 12 | 456 | 5.04 | 48495.41 | plastid? |  |  |
|  | GRMZM2g536120 | ANK-TPR | ZmTPR201 | 10 | 8 | 376 | 5.11 | 40699.96 | plastid? Secretary pathway? |  |  |
