# Supplementary material for: A cluster of Ankyrin and Ankyrin-TPR repeat genes is associated with panicle branching diversity in rice
Source: PLoS Genet. 2021 Jun 7;17(6):e1009594. doi: 10.1371/journal.pgen.1009594 (PMC8211194; doi:10.1371/journal.pgen.1009594)
Supplement: S8 Fig — Schematic view of the exon-intron structure of the ANK genes. Blue box: coding exon; white box: non-coding exon; blue line: intron; black line: promoter region. The structure of the LOC_Os02g29210 gene was corrected according the in lab sequence. Polymorphic sites are indicated with a black vertical line for SNPs and triangles for INDELs. INDEL lengths are indicated in bp. SNPs causing non-synonymous mutations are indicated in red and corresponding variations at nucleic and amino acid levels are indicated. (PDF) [file pgen.1009594.s008.pdf]

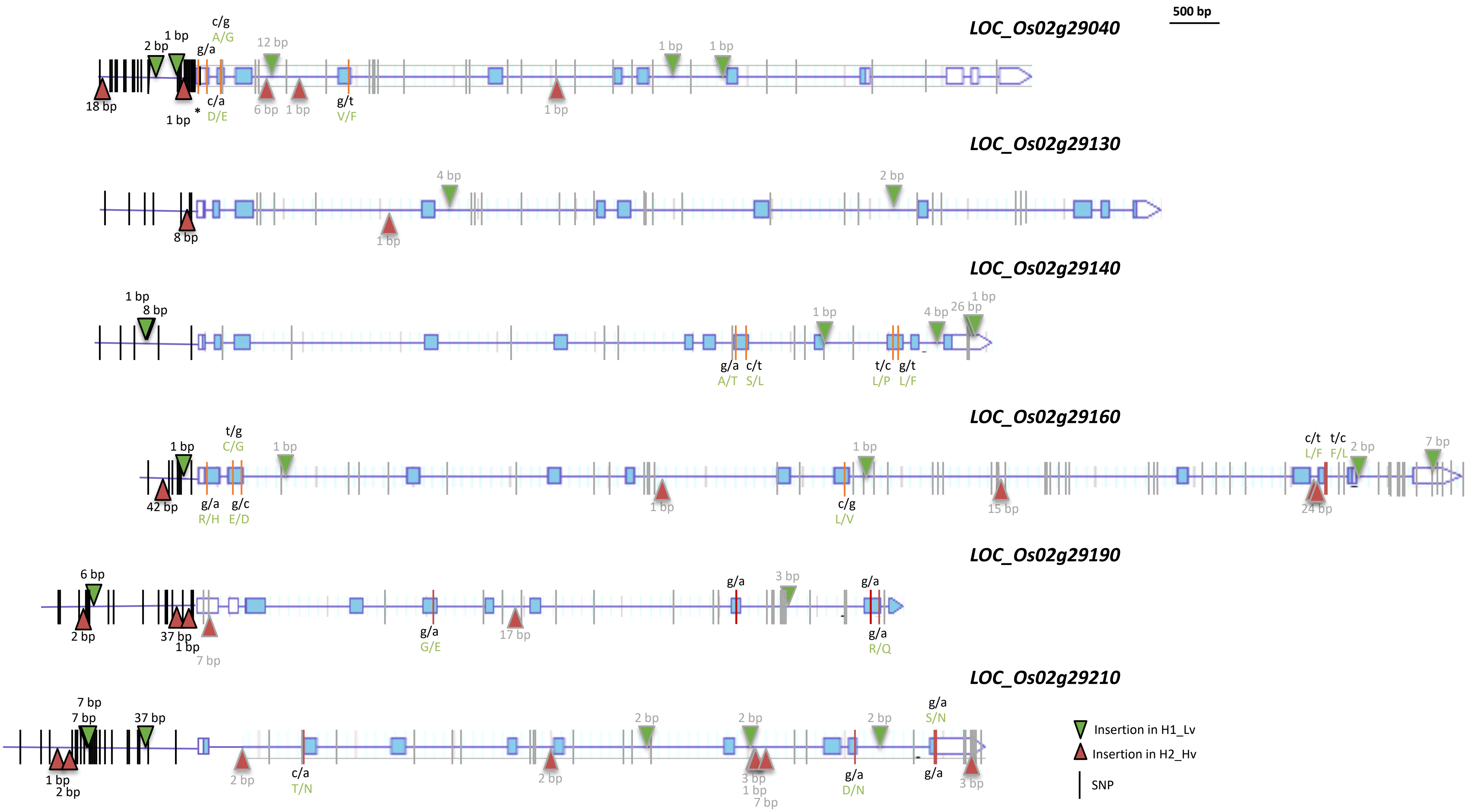

500 bp
LOC_Os02g29040
LOC_Os02g29130
LOC_Os02g29140
LOC_Os02g29160
LOC_Os02g29190
LOC_Os02g29210
c/g
A/G
g/a
2 bp
1 bp
12 bp
18 bp
1 bp
c/a
D/E
6 bp
1 bp
g/t
V/F
4 bp
2 bp
8 bp
1 bp
g/a
A/T
c/t
S/L
t/c
L/P
g/t
L/F
26 bp
t/g
C/G
g/a
R/H
g/c
E/D
42 bp
c/g
L/V
15 bp
c/t
L/F
t/c
F/L
24 bp
7 bp
6 bp
37 bp
17 bp
g/a
G/E
3 bp
R/Q
S/N
T/N
D/N
Insertion in H1_Lv
Insertion in H2_Hv
SNP
