# Supplementary material for: A cluster of Ankyrin and Ankyrin-TPR repeat genes is associated with panicle branching diversity in rice
Source: PLoS Genet. 2021 Jun 7;17(6):e1009594. doi: 10.1371/journal.pgen.1009594 (PMC8211194; doi:10.1371/journal.pgen.1009594)
Supplement: Attachment — Submitted filename: Response to reviewers_R1_final.doc [file pgen.1009594.s024.doc]

IRD, UMR DIADE

BP 64501, 911 avenue Agropolis

34394 Montpellier Cedex 5, France

Tel.: +33 4 67 41 62 41

Fax: +33 4 67 41 62 22

Prof. Sarah Hake (Associate Editor)

and Prof. Li-Jia Qu (Section Editor: Plant Genetics)

PLOS Genetics

27 April 2021

re : revision of manuscript "A cluster of Ankyrin and Ankyrin-TPR repeat genes is associated with panicle branching diversity in rice" (PGENETICS-D-21-00326) by Khong NG et al. for publication in PLOS Genetics

Dear Prof. Hake and Prof. Li-Jia Qu

Thank you for your e-mail of 1^st^ April 2021 relating to the abovementioned manuscript.

We read with interest the comments of the reviewers, along with those of the Handling Editor. We found all the comments to be very relevant and useful for the improvement of our manuscript. We were pleased to note that Prof. Li-Jia Qu considered the body of data presented our manuscript to be sufficient, subject to suitable revisions of the text and figures, for publication in PLOS Genetics without the need for additional experiments. Accordingly, we have made modifications in line with the recommendations and have pleasure in submitting a revised form of the manuscript which we believe addresses all the points raised. We are also pleased to provide additional clarifications in our responses below:

**1) Comments of Handling Editor (Professor Li-Jia Qu)**

*I don’t think you need to do more experiments, but please respond to the reviewer’s comments. I agree with the reviewers that the in situs are not helpful. I am glad you tried them, but they don’t tell us anything. You could put that into supplemental data. I don’t think you need figure 7. You don’t really have enough data to show this without the additional experiments suggested by reviewer 1.*

We agree with these two comments. Accordingly, panel C of Figure 6 is now Supplementary Figure S18 in the revised manuscript. Figure 7 has been deleted in the revised manuscript.

*Here are some minor comments. Make it clear that the ankyrin genes in S5, are different than the ones in figure 3*

The expression level plots shown in Figure S5 correspond to panicle-expressed genes located in QTL_9 which do not belong to the *ANK* or *ANK-TPR* gene families. In order to clarify this, the *ANK* and *ANK-TPR* genes from QTL_9 are now indicated by red asterisks in Figure S5. In Figure 3, only the expression patterns of *ANK* and *ANK-TPR* genes are shown. The expression patterns of the two other genes in the “ANK cluster” that are expressed during panicle development but which do not belong to the *ANK* or *ANK-TPR* gene families are now shown in Figure S5.

*The section on SNPs is tedious, especially since you don’t actually test any of these SNPs. I think you can try and summarize most of it in a figure rather than writing it out. (For example, you could eliminate lines 193-202). Lines 219-239 could also be shortened.*

Modifications have been carried out accordingly on the revised manuscript (lines 198-202 and 222-236 in the clean copy of the revised manuscript) as well as in Figure S9 in which the two non-synonymous polymorphic sites located in ANK domains have been highlighted.

*Figure 4A needs more information. The legend says it is just exon 1, but then what are the different parts? I like to look at a figure and understand it without reading the legend and I am still confused.*

Figure 4A has been modified (explanatory details have been added in the figure) and the corresponding legend has been altered to clarify the content of the tables of polymorphic sites for each promoter region.

*Figure 5. what is null sister? Non-CRISPR? Using the word null for non-mutant is confusing.*

The “null sister” term was used to describe a CRISPR-Cas9 transgenic line without any mutation at the targeted sites. We agree that this term is confusing. To clarify this, we now use the term “sister” in the box-plots in Figure 5 and in the annotation of the photos in Figure 5. The corresponding text in the Materials and Methods section has been modified.

*Lines 383-390 seem unnecessary in the discussion.*

This part of the discussion has now been edited in the revised manuscript (lines 381- 388)

**2) Comments of Reviewer #1:**

*1. There has no direct evidence confirming the correlation between H1, H2 haplotypes and their gene functions, it’s difficult to understand why the authors deduced that the H1 and H2 haplotypes, instead of the other 22 distinct haplotypes in the putative genomic region contribute to the SpN and SBN traits?*

The selected H1 and H2 haplotypes represent two of the three most common haplotypes in the collection (see Figure S2A), are represented in 22 and 27 accessions respectively (within the overall group of 159 accessions) and diverge in sequence by a large genetic distance. In terms of their association with phenotypic traits, a clear contrast could be seen between the haplotypes H1 and H2 in relation to SpN and SBN traits when the 24 distinct haplotypes were studied, as illustrated below in Figure A:

Figure A: Association between the 24 haplotypes for QTL_9 and the spikelet and secondary branch numbers (SpN and SBN respectively) for the field assay in 2015. The haplotypes H1 and H2 correspond to the groups III and IX respectively. The number of accessions per haplotype are indicated below

Even if more extreme haplotypes were characterized, in terms of association with SpN and SBN traits, the H1 and H2 haplotypes should represent most of the association evidenced by GWAS since they correspond to a larger number of accessions. Consequently, these two haplotypes were selected to maximize the chances of identifying by sequencing the common putative polymorphisms associated with phenotypic diversity. It should also be noted that these two haplotypes were mainly or totally constituted by *indica* accessions, thus limiting the possibility of false positive effects on GWAS of the strong genetic structuration of the panel used (*japonica* vs. *indica*).

To clarify this point, the corresponding section of the text has been modified in the revised manuscript (lines 121-122)

*And is there any evolution clues for H1 and H2 haplotype? Does the MUTE- or MITE- derived sequences in the promoter region help the H2 haplotype evolve from H1 Haplotype?*

Based on the full dataset from the 3K genome project, and considering the promoter regions from the 4 genes of interest, about 90% and 80% of the accessions in the present study sharing H1 and H2 haplotypes respectively are *indica.* The two haplotypes are shared by respectively 16% and 18% of *indica* accessions in the 3K panel. This information has been added to the Results section (lines 215-221). The presence of these two haplotypes in a few *japonica* and *aus*/*boro* accessions could be explained by admixture/hybrid accessions or mis-assigned genotypes. The deletions in the promoter regions of the 4 genes of interest in QTL_9, mainly observed in the H1 haplotype, were not present in the unique *O. rufipogon* genome considered here, suggesting an origin after domestication, as mentioned in the discussion (lines 336-339). Nevertheless, based on the available genomic data from the 3K genomes project, it is difficult to date or explain their origin. The H1 promoter regions have evolved at least in part by deletions which occurred within MITE or MUTE elements present in both *indica* and *japonica* accessions as well as in *O. rufipogon*. Therefore, the H2 haplotype does not seem to have evolved from the H1 haplotype. It is difficult to address the interesting question as to how MUTE and MITE sequences might have contributed to their evolution. The important point is that MUTE or MITE-derived sequences are known to provide new sequences in the vicinity of genes that could carry new regulatory information such as new TF binding sites that may be conserved during domestication and post-domestication diversification. More generally, such TF binding sites may be differentially conserved during evolution. This point is discussed in the manuscript (lines 340-342).

*Will the introduction of indica H1 haplotype into japonica background affect panicle development?*

Considering promoter deletion polymorphisms, the *japonica* background (Nipponbare and Kitaake) is more similar to the *indica* H2 haplotype with respect to *LOC_Os02g29040*, *LOC_Os02g29160* and *LOC_Os02g29190*, but to the H1 haplotype for *LOC_Os02g29210* (Figure 4); considering CDS non-synonymous polymorphisms, the *japonica* background is similar to H2 for *LOC_Os02g29040* but to neither H1 nor H2 for the other genes (Figure S9). In this light, it can be assumed that the introduction of the *indica* H1 haplotype into a *japonica* background will affect panicle development. An experiment would be to generate NILs with *indica* H1 haplotype in *japonica* background for the ANK/ANK-TPR cluster region. As an alternative to NILs, an interesting but still rather laborious experiment would be to induce promoter deletions using CRISPR-Cas9 in the *japonica* background, as seen in the *indica* H1 haplotype for *LOC_Os02g29040*, *LOC_Os02g29160* and *LOC_Os02g29190.* It would also be of interest to overexpress the *indica* allelic forms in a *japonica* background in order to demonstrate whether the CDS polymorphism by itself can have an impact on panicle structure. Nevertheless, our functional analysis of *LOC_Os02g29040* and *LOC_Os02g29210* in the *japonica* variety provides at least partial evidence that the *indica* H1 haplotype can affect panicle development in this genetic background. It can be assumed that the *japonica* *ank1* mutants produced by CRISPR-Cas9-induced deletions in *LOC_Os02g29040* could reflect in part the *indica* H1 haplotype effect in a *japonica* background, as illustrated by the alteration of SBN and SpN traits.

*In Fig 2, the panicles of F3_H1 and F3_H3 are smaller than their parents, does it mean there have other loci contribute to panicle development? To my opinion, we can’t make a definite conclusion without NIL lines analysis between H1 and H2 haplotypes.*

We agree that the panicles of F3_H1 and F3_H3 are smaller than their parents and that the difference between the two haplotypes is greater in the parents than between the F3 lines. The F3 population was selected only on the QTL_9 region. Therefore, we agree that the phenotypic results support the idea that several QTLs are involved in panicle architecture diversity between the two parents. However, these observations are also in agreement with the GWAS dataset of panicle morphological traits from the accession panel which showed that the diversity of these traits is associated with a large number of QTLs of low effect sizes (Ta et al., 2018). We agree that the definitive genetic proof for QTL_9 contribution to panicle diversity would be to analyse corresponding NILs; however the F3 population from this study does already give information on the contribution of QTL_9 to panicle diversity.

*2. Regarding to the biological functions of the 6 ANK and ANK-TPR genes, although expression pattern analysis showed that the LOC_Os02g29040, LOC_Os02g29160 and LOC_Os02g29210 have special expression pattern in early panicle development, the in situ hybridization data is not qualified enough to observe any difference between them (TAW1 and LHS1 are suggested to be used for system control). Furthermore, the expression pattern of LOC_Os02g29160 seems quite different to that of LOC_Os02g29210 in Fig.6B. I am afraid the higher expression level at different developmental stages couldn’t explain why the author classified them into two groups. I suggest the author validate their hypothesis by performing promoter swap experiment between LOC_Os02g29040 and LOC_Os02g29210.*

We agree with the comment from Reviewer 1 about the *in situ* hybridization data. The expression patterns are quite similar for these genes with expression detected in the various different meristem types. Since the *in situ* hybridization detection technique provides essentially qualitative information, it cannot be considered as suitable to demonstrate differential expression between distinct developmental stages. As suggested by the Handling Editor, the corresponding data has been deleted from Figure 6 and used as supplementary data (Figure S18).

Concerning the classification of the *ANK* and *ANK-TPR* genes according to their expression patterns, we believe that some clarification is necessary. Based on the expression profiling in *O. sativa* cv. Nipponbare, the *ANK* and *ANK-TPR* genes were classified in two distinct groups regarding their expression during panicle development (Fig. 6B). However, the genes were also classified in separate way by comparing *O. sativa indica* accessions of H1 and H2 haplotypes independently of developmental stage (Fig 3C). The expression patterns of the *ANK* and *ANK-TPR* genes during panicle development in *indica* accessions is consistent with the data from Nipponbare, with the exception of *LOC_Os02g29040* gene. In this context, *LOC_Os02g29160* and *LOC_Os02g29210* genes were classified into separate groups based on their expression patterns during panicle development in the Nipponbare background but in the same group for their expression level behaviour between the two *indica* haplotypes.

The promoter swap experiment suggested by Reviewer 1 between *LOC_Os02g29040* and *LOC_Os02g29210* will be of interest in order to determine if the function of these genes as positive or negative regulators of meristem fate transition might be related to their expression pattern during panicle development. A promoter swap experiment between the two *indica* haplotypes (or CRISPR-Cas9 induced deletions) would also be of great interest in order to evaluate the potential impact of promoter sequence polymorphisms and to determine the relative importance of the intragenic and promoter polymorphisms.

*3. Following the above question, how about the expression level of LOC_Os02g29210 in the over-expression line of LOC_Os02g29040, as well as in the ank1 mutants? Same to the LOC_Os02g29040, will the expression level of LOC_Os02g29040 change in the ank2 mutants? How about the expression level of other homologous genes in these transgenic lines? And how about the expression pattern of those marker genes, including TAW1, LHS1 and DES4 in these transgenic lines?*

The expression levels of the *ANK* and *ANK-TPR* expressed genes between H1 and H2 *indica* haplotypes as well as *TAW1* gene have been analysed using qRT-PCR in conjunction with panicle-derived cDNAs in the different transgenic lines from *japonica* background. Only one line per type of transgenic plant with the strongest phenotypic effect was considered for this analysis. Panicle-derived cDNAs correspond to “early branching” stage (Figure B below).

In OE-ANK1 and *ank2* lines, which are associated with a higher number of SBs and spikelets, the *TAW1* gene was overexpressed compared to WT plants. This feature is coherent with the function of *TAW1* acting as an inhibitor of the transition to spikelet identity, thereby favoring branching (Yoshida et al. 2013). Unfortunately, we failed to get data from *ank1* lines in order to observe a putative opposite behavior.

In all the tested lines (ANK1-OX, *ank1* and *ank2*), the *LOC_Os02g29210* gene was overexpressed independently of the line’s phenotype. In parallel, the *LOC_Os02g29040, LOC_Os02g29160* and *LOC_0s02g29190* genes were overexpressed in both *ank1* and *ank2* lines but not affected in ANK1-OX line, suggesting a common negative regulation through *LOC_Os02g29040* and *LOC_Os02g29210.* However, the absence of repression of these 3 genes in the ANK1_OX line would suggest that LOC_Os02g29040 is necessary but that other factor(s) may limit its action with regard to these genes except for *LOC_Os02g29210*. The latter two genes display distinct patterns in transgenic lines, indicating that they might be regulated through different pathways. The *LOC_Os02g29130* gene is down-regulated in all lines tested and *LOC_Os02g29140* is down-regulated in the *ank2* line but up-regulated in the ANK1_OX line.

Figure B: Expression profiling of *ANK*, *ANK-TPR* and *TAW1* genes at “early branching” stage in OE and CRISPR lines.

Based on the results that we obtained from two distinct biological replicates, it is difficult to conclude on the nature of the interactions between the aforementioned genes in terms of expression levels and how they could relate to the differential expression between *indica* accessions. We are aware of the weakness of this point with regard to our hypothesis of the association between the panicle branching complexity and the relative expression levels of these genes between H1 and H2 haplotypes in *indica* background. However, it has to be stressed that the situation in a *japonica* transgenic plant with a single mutated gene does not represent the complexity of interactions occurring between these genes in the different *indica* haplotypes.

*4. I am not quite sure about the phenotypes presented in the Fig.5, the wild type Kitaake has the lest number of secondary branches and spikelets than all the other lines? And the panicle of Kitaake seems much smaller than a normal inbreed lines. How many plants and how many panicles were collected for yield traits analysis?*

We agree that the Kitaake inbreed line was characterized by small panicles in our conditions in Vietnam. However, this does not devalue our phenotypic analyses as the comparison of the transgenic versus wild-type plants were conducted in the same conditions. The only exception is that the analysis of the OE lines with the WT Kitaakee were not conducted at the same time as the analysis of the CRISPR versus the sister lines. Consequently, the phenotypic values of wild-type plants and those of the sister lines are not directly comparable.

For each line, 18 panicles were collected from 6 distinct plants, using the 3 main panicles for each plant. For the other traits (tiller number, flowering time), 6 plants per line were considered. This point has been clarified in the “Materials and Methods” section of the revised manuscript (lines 509-511).

*5.There has no direct evidence for validating the regulatory role of LOC_Os02g29160 and LOC_Os02g29190 in panicle development. I don’t think list them in Fig. 7 is correct. Furthermore, in the current model, LOC_Os02g29040 may be a repressor during transition from branch meristems to spikelet meristems, while LOC_Os02g29210 functions as an activator, could the author explain why they have distinct roles in meristem activity.*

We agree with the comment from reviewer 1 concerning *in situ* hybridization experiment. As mentioned earlier and suggested by the Handling Editor, Figure 7 has been deleted.

Our current model is based on the fact that both the higher expression of *LOC_Os02g29040* and the lower expression of *LOC_Os02g29210* are associated with higher secondary branching in H2 *indica* accessions compared to H1 *indica* accessions. Consequently *LOC_Os02g29040* and *LOC_Os02g29210* might act as respectively an activator and a repressor of secondary branching. As mentioned in the discussion (lines 396-400), “panicle architecture diversity, notably in terms of spikelet number variation, can be explained at least in part by the fine-tuning of axillary meristem fate on primary branches, which affects the balance between secondary branch (indeterminate) and spikelet (determinate) meristems. In this way, a more highly branched panicle can be obtained by a delayed or lower rate of spikelet meristem fate acquisition”. In this context, an activator of secondary branching such as *LOC_Os02g29040* could be considered as a repressor of transition from indeterminate to determinate axillary meristems, favouring secondary branch establishment instead of axillary spikelets. Conversely, a repressor of secondary branching such as *LOC_Os02g29040* could be considered as an activator of transition from indeterminate to determinate axillary meristems.

To clarify this point, the corresponding text in the discussion has been modified in the revised version of the manuscript (lines 391 to 396).

*6. Which generation of ank1 and ank2 were used for phenotype analysis?*

For the *ank1* and *ank2* lines, T2 T-DNA free plants homozygous for the deletion were used for phenotypic analysis. This information has been added in the “Materials and Methods” section of the revised manuscript (line 509).

*7.No figure legends in Fig.7, and in Fig. S5, the icon size are different.*

Figure 7 has been deleted and the Figure S5 has been modified.

**3) Comments of Reviewer #2:**

*They make the claim that these genes mediate the transition from indeterminate to determinate meristems, although I feel that this is too strong an interpretation, and that RNAseq analysis of developing panicles will be needed to find co-regulated genes and to identify what pathways the ANK genes participate in.*

We agree with reviewer 2 that our interpretations need to be considered as a hypothesis, which is based on the fact that panicle architecture complexity is determined in part by the rate of transition from indeterminate to determinate fate in primary branch axillary meristems (see the answer to question 5 from reviewer 1). Our earlier work performing RNAseq analysis on laser-dissected panicle meristems in *O. sativa* Nipponbare (Harrop et al. 2016) provided a list of co-expressed genes of interest in the different meristem types. Some of the TF genes identified are listed in Supplementary File S13. As a complementary experiment and as suggested by reviewer 2, it will be of great interest to carry out RNAseq analysis on both OE and CRISPR lines with the aim of identifying in what pathways the ANK genes participate.

*1) the description of the sampling periods ('early' and 'late') for RNA analysis should be explained more fully in the results, as well as its significance. At the moment it is in the M&M, but that puts it well after the Results section.*

Panicle developmental staging was defined as by two categories: “early branching” (from inflorescence meristem stage to panicle with elongated primary and higher order branch development); and “late branching” (from panicle with elongated primary and secondary branches to young flowers with differentiated organs), the first being enriched in meristems of an indeterminate state and the second enriched in meristems at a determinate state. More explanation has been added to the “Results” section (lines 151 to 155) and “Materials and Methods” section (lines 584 to 585).

*2) Fig. 6C - it is not clear to me that these illustrate the high and low regulation of these genes, or are specific enough to be clear of their patterns of expression.*

The *in situ* hybridization experiment presented here confirmed that these genes are expressed within the meristems (but without any specific zonal patterning), with no signal detected in any other part of the panicle. We do therefore agree that this pattern is not specific enough to provide clear information about their differential functions. The *in situ* hybridization technique used is a qualitative method and is not sufficient in itself to infer differential expression during distinct developmental stages. As suggested by the Handling Editor, panel C of Figure 6 has been changed to Supplementary Figure S18.

In conclusion, we hope that our corrections and additions to the text, along with the above clarifications, address the various concerns of the reviewers and the Handling Editor. We believe that the various comments and criticisms have allowed us to produce a stronger manuscript and we hope very much that it will now be acceptable for publication in your journal.

We look forward to hearing from you further.

Yours sincerely,

Stefan Jouannic

(for the authors)
